# Effectiveness of Transdiagnostic Cognitive-Behavioral Psychotherapy Compared With Management as Usual for Youth With Common Mental Health Problems: A Randomized Clinical Trial

**DOI:** 10.1001/jamapsychiatry.2020.4045

**Published:** 2020-12-23

**Authors:** Pia Jeppesen, Rasmus Trap Wolf, Sabrina M. Nielsen, Robin Christensen, Kerstin Jessica Plessen, Niels Bilenberg, Per Hove Thomsen, Mikael Thastum, Simon-Peter Neumer, Louise Berg Puggaard, Mette Maria Agner Pedersen, Anne Katrine Pagsberg, Wendy K. Silverman, Christoph U. Correll

**Affiliations:** 1 Child and Adolescent Mental Health Centre, Mental Health Services–Capital Region of Denmark, Copenhagen, Denmark; 2 Department of Clinical Medicine, Faculty of Health and Medical Sciences, University of Copenhagen, Copenhagen, Denmark; 3 Danish Centre for Health Economics, Department of Public Health, University of Southern Denmark, Odense; 4 Musculoskeletal Statistics Unit, Parker Institute, Bispebjerg and Frederiksberg Hospital, University of Copenhagen, Copenhagen, Denmark; 5 Research Unit of Rheumatology, Department of Clinical Research, University of Southern Denmark, Odense University Hospital, Odense, Denmark; 6 Division of Child and Adolescent Psychiatry, Department of Psychiatry, Lausanne University Hospital, Lausanne, Switzerland; 7 Department for Child and Adolescent Psychiatry, Mental Health Services in the Region of Southern Denmark, Odense, Denmark; 8 Department of Clinical Research, University of Southern Denmark, Odense, Denmark; 9 Research Center at the Department for Child- and Adolescent Psychiatry, Aarhus University Hospital, Skejby, Denmark; 10 Institute of Clinical Medicine, Aarhus University, Aarhus, Denmark; 11 Centre for the Psychological Treatment of Children and Adolescents, Department of Psychology and Behavioural Sciences, Aarhus School of Business and Social Sciences, Aarhus University, Aarhus, Denmark; 12 Centre for Child and Adolescent Mental Health, Oslo, Norway; 13 Centre for Child and Youth Mental Health and Child Welfare, The Arctic University of Norway, North Norway (RKBU North), Tromsø, Denmark; 14 Anxiety and Mood Disorders Program, Yale Child Study Center, Yale University School of Medicine, New Haven, Connecticut; 15 Donald and Barbara Zucker School of Medicine at Hofstra/Northwell, Department of Psychiatry and Molecular Medicine, Hempstead, New York; 16 Department of Psychiatry, The Zucker Hillside Hospital, Glen Oaks, New York; 17 Center for Psychiatric Neuroscience, Feinstein Institute for Medical Research, Manhasset, New York; 18 Department of Child and Adolescent Psychiatry, Charité Universitätsmedizin, Berlin, Germany

## Abstract

**Importance:**

Behavioral therapy and cognitive-behavioral therapy (CBT) programs targeting a single class of problems have not been widely implemented. The population of youths with common mental health problems is markedly undertreated.

**Objective:**

To determine the effectiveness of a new transdiagnostic CBT program (Mind My Mind [MMM]) compared with management as usual (MAU) in youths with emotional and behavioral problems below the threshold for referral to mental health care.

**Design, Setting, and Participants:**

This pragmatic, multisite, randomized clinical trial of MMM vs MAU was conducted from September 7, 2017, to August 28, 2019, including 8 weeks of postintervention follow-up, in 4 municipalities in Denmark. Consecutive help-seeking youths were randomized (1:1) to the MMM or the MAU group. Main inclusion criteria were age 6 to 16 years and anxiety, depressive symptoms, and/or behavioral disturbances as a primary problem. Data were analyzed from August 12 to October 25, 2019.

**Interventions:**

The MMM intervention consisted of 9 to 13 weekly, individually adapted sessions of manualized CBT delivered by local psychologists. The MAU group received 2 care coordination visits to enhance usual care.

**Main Outcomes and Measures:**

The primary outcome was change in mental health problems reported by parents at week 18, using the Strengths and Difficulties Questionnaire (SDQ) Impact scale (range, 0-10 points, with higher scores indicating greater severity of distress and impairment). Primary and secondary outcomes were assessed in the intention-to-treat population at week 18. Maintenance effects were assessed at week 26.

**Results:**

A total of 396 youths (mean [SD] age, 10.3 [2.4] years; 206 [52.0%] boys) were randomized to MMM (n = 197) or MAU (n = 199), with primary outcome data available in 177 (89.8%) and 167 (83.9%), respectively, at 18 weeks. The SDQ Impact score decreased by 2.34 points with MMM and 1.23 with MAU, from initial scores of 4.12 and 4.21, respectively (between-group difference, 1.10 [95% CI, 0.75-1.45]; *P* < .001; Cohen *d* = 0.60). Number of responders (≥1-point reduction in SDQ Impact score) was greater with MMM than with MAU (144 of 197 [73.1%] vs 93 of 199 [46.7%]; number needed to treat, 4 [95% CI, 3-6]). Secondary outcomes indicated statistically significant benefits in parent-reported changes of anxiety, depressive symptoms, daily functioning, school attendance, and the principal problem. All benefits were maintained at week 26 except for school attendance.

**Conclusions and Relevance:**

In this randomized clinical trial, the scalable transdiagnostic cognitive-behavioral intervention MMM outperformed MAU in a community setting on multiple, clinically relevant domains in youth with emotional and behavioral problems.

**Trial Registration:**

ClinicalTrials.gov Identifier: NCT03535805

## Key Points

**Question:**

Can a transdiagnostic modular cognitive-behavioral therapy (CBT) program outperform management as usual for youth with emotional and behavioral problems?

**Findings:**

In this randomized clinical trial of 396 youths aged 6 to 16 years, the parent-reported functional impairment was significantly reduced for youth allocated to transdiagnostic modular CBT compared with management as usual. Key secondary outcomes also indicated a broad range of benefits.

**Meaning:**

This pragmatic study adds to the growing evidence that the wide-scale implementation of transdiagnostic modular CBT in nonspecialist care settings provides timely indicated prevention and quality care for help-seeking youth.

## Teaser

This randomized clinical trial assesses the effectiveness of a new transdiagnostic cognitive-behavioral therapy program compared with management as usual in youths with emotional and behavioral problems below the threshold for referral to mental health care.

## Introduction

Mental health disorders constitute the largest disease burden among children and adolescents (herein referred to as youths), and 50% of mental disorders begin before 14 years of age.^1,2^ Youths with mild to moderate symptoms of anxiety, depression, or behavioral disturbances have an increased risk of adverse adult outcomes.^3^ Evidence suggests that cognitive-behavioral therapies (CBTs) are effective for the indicated prevention and treatment of childhood anxiety,^4,5,6^ depression,^7,8,9^ and behavioral difficulties.^10,11^ Nevertheless, access to evidence-based prevention and treatment interventions is limited, because those interventions available are rarely used routinely in clinical practice.^12,13^ This knowledge-practice gap requires bridging by pragmatic trials testing the real-world implementation of scalable interventions for common mental health problems among youths.^12,14^ The transdiagnostic Modular Approach to Therapy for Children (MATCH) targets the most common mental health problems in youth.^15^ Two cluster-randomized clinical trials demonstrated the benefits of the MATCH manual (Child STEPs) compared with disorder-specific CBT and/or usual care on symptoms 1 and 2 years after enrollment,^15,16^ with no effects on functioning^16^ but reduced auxiliary service use in treatment completers.^17^ However, the only individual-level randomized clinical trial (RCT) of the Child STEPs found no superiority compared with usual care.^18^ More research is therefore needed to investigate the feasibility and effectiveness of transdiagnostic, modular interventions for large-scale implementation of evidence-based treatments for youth.

We developed a new transdiagnostic CBT program for indicated prevention and early treatment of youth problems below the threshold for psychiatric referral. Our program (Mind My Mind [MMM]) consisted of manualized CBT with modules, worksheets, play materials, case examples, and flowcharts guiding individually adapted treatment; standardized procedures for identifying/monitoring the target problem; and education/supervision of psychologists using video observation–based feedback. A feasibility RCT (152 participants, randomized 3:1) of MMM vs management as usual (MAU) in 4 municipalities in Denmark showed acceptability and feasibility and provided data for sample size estimation.^19^ The present definitive RCT evaluated the effectiveness of MMM vs MAU.

We hypothesized that the parent-reported impact of mental health problems would be significantly improved for youth receiving MMM vs MAU after the 17-week intervention. The secondary objectives were comparing the effectiveness of MMM vs MAU on parent-, self-, and teacher-reported outcomes of psychopathology, daily functioning, school attendance, health-related quality of life, and potential harms at weeks 18 (after the 17-week MMM intervention) and 26.

## Methods

### Study Design

The MMM study was a pragmatic, open-label, analyst-masked, parallel, 2-arm, randomized clinical superiority trial of MMM vs MAU for help-seeking youths with emotional and behavioral problems. The trial was conducted in the Educational-Psychological Advisory Services in 4 sociodemographically diverse municipalities in Denmark (Vordingborg, Næstved, Helsingør, and Holstebro). A copy of the trial protocol is available in Supplement 1. The trial conduct was overseen by a steering committee (also acting as the data and safety monitoring board) with representatives from Danish child and adolescent mental health services, municipalities, and the nongovernmental organization responsible for the program’s implementation (Supplement 1 and eMethods 1 in Supplement 2). The trial was approved by the scientific ethics committee, and the data management and protection complied with the European Union General Data Protection Regulation. Written informed consent signed by the legal guardians was provided for all the participants. There were no changes to trial methods after the trial commenced, and no data were analyzed before study completion and database lock. This study followed the Consolidated Standards of Reporting Trials (CONSORT) reporting guideline.

### Participants

Participants were recruited with help from local teachers, school health nurses, psychologists, general practitioners, and parents. Information was published online on school intranets and municipality websites and handed to the local professionals. Youth and parents contacted the Educational-Psychological Advisory Service to sign up for assessment of eligibility. No formal referral was required.

A 2-stage standardized screening of help-seeking youths was implemented in the municipalities to identify eligible study participants. The first stage used web-based questionnaires, including the Strengths and Difficulties Questionnaire (SDQ),^20^ and an algorithm combining parent-reported scores of emotional and behavioral problems and functional impairments to identify youth requiring care (inclusion criteria). In the second stage, youths underwent a semistructured, psychopathological interview to screen for developmental and mental disorders. Finally, the most important problem for which parents and youths wanted help was identified. This top problem was recorded using their own words,^21^ and the problem severity was scored from 1 to 10, with 10 indicating greatest severity.

Inclusion criteria consisted of (1) age of 6 to 16 years and in compulsory school; (2) anxiety symptoms, depressive symptoms, and/or behavioral disturbances as top problem; and (3) parent-reported SDQ Total Difficulties score of at least 14, Emotional Problems score of at least 5, and/or Conduct Problems score of at least 3, combined with an Impact score of at least 1 (ie, cutoff for 90th percentile of mental health problems in the general age-matched population).^22^ Exclusion criteria consisted of (1) prior clinical diagnosis of any developmental or mental disorder; (2) signs of intellectual disability or severe mental disorder, including autism-spectrum disorder, attention-deficit/hyperactivity disorder, psychotic disorder, eating disorder, obsessive-compulsive disorder, repeated self-harm, or alcohol or psychoactive drug abuse; (3) youth or parents unable to participate in weekly sessions; and (4) participation in the prior feasibility study (Supplement 1). Thus, we included help-seeking youths with emotional and behavioral problems that were above the 90th percentile in the general population of youth and still below the threshold for referral to specialized treatment in the child and adolescent mental health services.

### Randomization and Masking

The participants were randomized 1:1 to MMM vs MAU via centralized, computer-generated allocation sequences with permuted block sizes, stratified by geographical region (2 strata), age (6-10 or 11-16 years), and the top problem (anxiety, depressive symptoms, or behavioral problems). The Data and Documentation division in Corporate Quality–Central Denmark Region (Defactum) managed the online randomization and data-entry system and kept the person identification lists separated from the researchers. Defactum had no role in data analyses. Because of the nature of the interventions, only the evaluators and analysts were masked to group allocation.

### Interventions

The MMM interventions consisting of 9 to 13 weekly sessions were developed based on a systematic literature search (eMethods 2 and 3 in Supplement 2), from which we distilled 50 CBT methods/techniques from evidence-based programs targeting anxiety, depression, or behavioral disturbances as single disorders. The methods/techniques were organized into 35 problem-specific or generic modules. Flowcharts describe the sequencing and dosing of modules depending on the top problem and possibilities for flexible adaptations to the individual co-occurring problems (eFigures 1-3 in Supplement 2). In case of behavioral problems, the main course was parent management training (ages 6-13 years) or youth training of social, communicative, and adaptive skills. All parents were engaged as coagents of change. The MMM intervention was completed within 17 weeks, followed by a booster session after 4 weeks. The MMM training was provided by 24 psychologists (22 [91.7%] female; mean [SD] age, 39.5 [10.8] years) who were appointed by their local leader. Seventeen psychologists (70.8%) reported prior experience with nonmanualized CBT; 3 (12.5%) had a formal CBT education. The therapists received 1 week of training in the MMM treatment manual followed by weekly online individual (75%) or live group (25%) consultations with a local supervisor. The supervision processes had input from the video recordings of sessions and the online feedback from parents’ and youths’ top-problem scores. The therapists and their supervisors were offered three 1-day methodology courses and three 2-day booster trainings during the study period.

The youth and parents in the MAU group were offered 2 care visits to help coordinate usual care in the municipality (weeks 2 and 17). The MAU condition varied, because the youths could receive counseling, pedagogical advice, network meetings, educational support, or psychological treatment of various forms, either publicly or privately funded, or no further treatment (eMethods 4 in Supplement 2).

### Measures and Outcomes

Baseline assessments were performed using standardized online questionnaires to the youth, the parents, and the primary schoolteacher, including the Development and Well-being Assessment questionnaires and interviews.^23^ The Development and Well-being Assessment reports were rated by senior consultants to determine mental disorders according to the *DSM-IV* and *DSM-5*.

The primary outcome was the parent-reported change in the impact of mental problems at end of treatment (week 18), using the SDQ Impact scale from the Impact supplement. The 5-item SDQ Impact scale (range, 0-10)^20^ evaluates the impact of mental health problems on a child’s distress and functioning in home life, friendships, classroom learning, and leisure activities. The SDQ Impact scale has demonstrated good psychometric properties for capturing the functional impacts of emotional and behavioral problems in youths^20,24^ and for estimating the risk of concurrent and future disorder,^25^ service use,^24^ and impairments.^26^

The key secondary outcomes at week 18 were changes in (1) anxiety (Spence Children’s Anxiety Scale,^27^ parent-reported), (2) depressive symptoms (Mood and Feelings Questionnaire,^28^ parent-reported), (3) daily child functioning (Weiss Functional Impairment Rating Scale,^29^ parent-reported), (4) school attendance (proportion of school days attended within the last 4 weeks, parent-reported), (5) top-problem score (ideographic measure, parent-reported; range, 1-10),^21^ quality of life (KIDSCREEN-27),^30^ subscales for (6) physical well-being and (7) psychological well-being (child-reported), behavioral problems with the (8) Eyberg Child Behavior Inventory intensity score^31^ (parent-reported) and (9) Eyberg Child Behavior Inventory problem score^31^ (parent-reported) subscales, (10) total emotional and behavioral problems (SDQ Total Difficulties scale, parent-reported),^20^ (11) parent-reported response (SDQ Impact score reduction ≥1 point), and (12) parent-reported remission (SDQ algorithm scores below inclusion cutoff). We also explored changes in outcomes from baseline to week 26 (8 weeks after the MMM intervention’s cessation) with MMM vs MAU. The exploratory outcomes included the SDQ Impact and SDQ Total Difficulties scales scored by schoolteachers, child-reported outcomes (aged 11-16 years for SDQ, aged 8-16 years for Spence Children’s Anxiety Scale and Mood and Feelings Questionnaire), satisfaction, measured with the Experience of Service Questionnaire,^32^ and parental stress in role functioning, measured with the Parental Stress Scale.^33^ All outcomes were assessed at weeks 0, 18, and 26. Moreover, the SDQ Impact scale and top problem scale were scored biweekly until week 18. The parents reported all service use.

### Potential Harms

We assessed harms at weeks 18 and 26, defined by 2 binary composite scores (Supplement 1): (1) suicidality and negative cognitions (using 8 self-reported questions from the Mood and Feelings Questionnaire) and (2) poor quality of family relationships, free time, and friendships (using 9 self-reported questions from KIDSCREEN-27). For both composite scores, we applied the following rule: Harm was present if at least 1 question received a high score of 2 without an identical score at baseline. Professionals could report adverse events (without prespecified definitions) to the steering committee. Finally, all the children were followed up in Danish national registries for mortality.

### Statistical Analyses

Data were analyzed from August 12 to October 25, 2019. We designed the study with 90% power to detect a minimally relevant group difference of 1.0 point on the parent-reported SDQ Impact scale. With a 2-sided α = .05 and an SD of 2.7 (based on the feasibility trial^19^), follow-up data on at least 308 youths were required. We included 396 youths to allow for as much as 22% attrition, based on experiences from the feasibility trial. The primary and key secondary continuous outcomes were analyzed according to the intention-to-treat population using repeated-measures linear mixed models (ie, missing data were handled implicitly), including a factor for treatment group (2 levels) and time (9 levels), the interaction between both, and adjustments for baseline value and stratification factors.^34^ The least-squares means with 95% CIs were estimated and subsequently converted into Cohen effect sizes for interpretation. The responder indices were analyzed using logistic regression models, including a factor for group and adjustment for stratification factors, and conservatively assuming missing data to be from nonresponders. Odds ratios with 95% CIs were estimated and converted into approximate risk ratios, and numbers needed to treat (NNT) were calculated.

To explore the results’ robustness for the primary and key secondary outcomes, we performed sensitivity analyses on the per-protocol population (full data set at week 18) and on the population enrolled in the trial before and after the final trial registration was approved (ie, midway), as well as with missing data handled using multiple imputation. To explore whether the secondary outcomes were highly correlated and somewhat redundant with the primary outcome, we calculated the Spearman correlation coefficients for all change scores pairwise, in each intervention group separately.

For the exploratory outcomes, unadjusted differences in mean changes with 95% CIs were estimated for the continuous outcomes, and unadjusted risk ratios with 95% CIs were estimated for the dichotomous outcomes. All the statistical tests were performed 2-sided with α = .05. The key secondary outcomes were analyzed in a hierarchical fixed sequence with reporting of *P* values and claims of statistical significance until 1 of the analyses failed.^35^ All of the analyses were performed in SAS Studio (SAS Institute, Inc) and R, version 3.6.1 (R Project for Statistical Computing), with the nlme and emmeans packages.

## Results

### Trial Population

**Figure 1.**
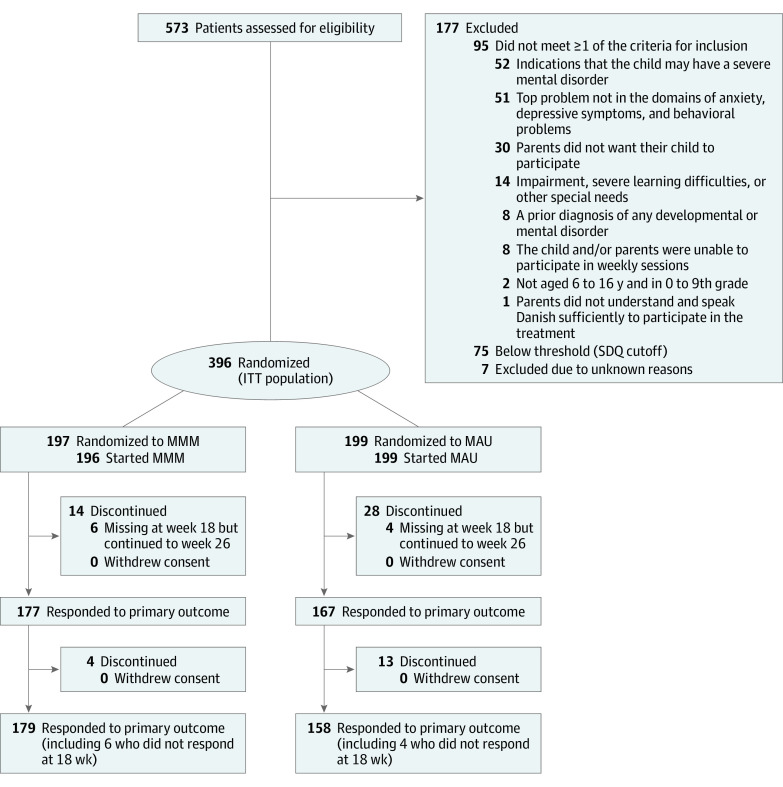
CONSORT Diagram of Participant Flow Through the Trial Flow includes screening, randomization, and 18- and 26-week follow-up.

As illustrated in Figure 1, from September 7, 2017, to December 18, 2018, 573 youths were screened for eligibility, and 396 were randomly assigned to MMM (n = 197) or MAU (n = 199) in the Educational-Psychological Advisory Services of 4 Danish municipalities. The baseline group characteristics were similar (Table 1). The youths’ mean (SD) age was 10.3 (2.4) years; 190 (48.0%) were girls and 206 (52.0%) were boys. Anxiety was the most common top problem (231 [58.3%]), followed by behavioral problems (101 [25.5%]) and depression (64 [16.2%]). The mean (SD) parent-reported SDQ Impact score was 4.16 (2.39), corresponding to moderate impact in 4 of 5 domains. Three hundred seventeen youths (80.1%) fulfilled the diagnostic criteria for at least 1 *DSM-IV* or *DSM-5* mental disorder, and 102 (25.8%) had comorbid disorders across domains (eg, anxiety and depression).

**Table 1.** Baseline Characteristics of the Participants in the ITT Population

Characteristic	Study group^a^		
	MMM (n = 197)	MAU (n = 199)	All (N = 396)
Demographic			
Girls	90 (45.7)	100 (50.3)	190 (48.0)
Age, mean (SD), y	10.3 (2.43)	10.3 (2.32)	10.3 (2.37)
Age group, y			
6-10	109 (55.3)	105 (52.8)	214 (54.0)
11-16	88 (44.7)	94 (47.2)	182 (46.0)
Region			
Holstebro-Helsingør	95 (48.2)	96 (48.2)	191 (48.2)
Vordingborg-Næstved	102 (51.8)	103 (51.8)	205 (51.8)
Principal domain of problems			
Anxiety	114 (57.9)	117 (58.8)	231 (58.3)
Depressive symptoms	31 (15.7)	33 (16.6)	64 (16.2)
Behavioral problems	52 (26.4)	49 (24.6)	101 (25.5)
Developmental delays			
Language	30 (15.2)	26 (13.1)	56 (14.1)
Any other^b^	62 (31.5)	48 (24.1)	110 (27.8)
School absenteeism >4 wk last year	33 (16.8)	39 (19.6)	72 (18.2)
No. of *DSM-IV/DSM-5* mental disorders based on DAWBA			
Anxiety disorder	102 (51.8)	118 (59.3)	220 (55.6)
Depressive disorder	25 (12.7)	33 (16.6)	58 (14.6)
Behavioral disorder	50 (25.4)	47 (23.6)	97 (24.5)
Neurodevelopmental disorder	25 (12.7)	32 (16.1)	57 (14.4)
Any disorder	153 (77.7)	164 (82.4)	317 (80.1)
Comorbidity, ≥2 disorders	45 (22.8)	57 (28.6)	102 (25.8)
Physical illness (asthma, diabetes, eczema, epilepsy, other)	34 (17.3)	37 (18.6)	71 (17.9)
Living arrangement			
Both parents	126 (64.0)	122 (61.3)	248 (62.6)
Single parent	32 (16.2)	41 (20.6)	73 (18.4)
Other/reconstituted family	39 (19.8)	36 (18.1)	75 (18.9)
Parent registered as informant			
Mother	169 (85.8)	173 (86.9)	342 (86.4)
Father	28 (14.2)	26 (13.1)	54 (13.6)
Mother’s highest educational level			
Elementary school (9-10 y)	11 (5.6)	15 (7.5)	26 (6.6)
High school/vocational (11-14 y)	49 (24.9)	63 (31.7)	112 (28.3)
Bachelor and above (15-17 y)	116 (58.9)	100 (50.3)	216 (54.5)
Higher education (≥17 y)	21 (10.7)	21 (10.6)	42 (10.6)
Immigration history of parents^c^			
2 Born in Denmark	182 (92.4)	176 (88.9)	358 (90.6)
1 Not born in Denmark	14 (7.1)	17 (8.6)	31 (7.8)
2 Not born in Denmark	1 (0.5)	5 (2.5)	6 (1.5)
No. of children in the household			
Index child only	23 (11.7)	23 (11.6)	46 (11.6)
2	11 (5.6)	119 (59.8)	230 (58.1)
≥3	63 (32.0)	57 (28.6)	120 (30.3)
Mother’s self-reported mental health problems			
Anxiety	10 (5.1)	27 (13.6)	37 (9.3)
Depression	111 (56)	31 (15.6)	42 (10.6)
Other	15 (7.6)	26 (13.1)	41 (10.4)
Both parents had mental health problems	5 (2.5)	11 (5.5)	16 (4.0)
Primary outcome measure, mean (SD)			
SDQ Impact scale score (parent-reported)^c^^,^^d^	4.12 (2.34)	4.21 (2.43)	4.16 (2.39)
Key secondary outcome measures, mean (SD)			
Anxiety (SCAS score [parent-reported])^e^	26.69 (15.65)	30.01 (15.35)	28.36 (15.57)
Depressive symptoms (MFQ score [parent-reported])^f^	16.22 (11.46)	17.34 (11.23)	16.78 (11.34)
Level of daily functioning (WFIRS score [parent-reported])^g^	31.13 (15.48)	31.19 (13.89)	31.16 (14.69)
School attendance (parent-reported)^h^	0.87 (0.22)	0.86 (0.22)	0.86 (0.22)
Top-problem score (parent-reported)	7.22 (1.78)	7.41 (1.74)	7.32 (1.76)
KIDSCREEN-27 score (self-reported), mean (SD), *t* value^i^			
Physical Well-being scale	45.58 (9.98)	43.22 (10.31)	44.40 (1.20)
Psychological Well-being scale	44.94 (10.64)	42.96 (8.40)	43.95 (9.62)
Behavioral problems (ECBI [parent-reported]), mean (SD)			
Intensity score^j^	107.33 (30.87)	107.70 (29.72)	107.52 (30.26)
Problem score^k^	11.01 (7.46)	10.97 (7.26)	10.99 (7.35)
Emotional and behavioral problems (SDQ Total Difficulties score [parent-reported])^l^	15.99 (5.25)	16.11 (5.49)^c^	16.06 (5.36)

Abbreviations: DAWBA, Development and Well-being Assessment; ECBI, Eyberg Child Behavior Inventory; ITT, intention-to-treat; MAU, management as usual; MFQ, Mood and Feelings Questionnaire; MMM, Mind My Mind; SCAS, Spence Children’s Anxiety Scale; SDQ, Strengths and Difficulties Questionnaire; WFIRS, Weiss Functional Impairment Rating Scale.

^a^ Unless otherwise indicated, data are expressed as number (percentage) of patients. Percentages have been rounded and may not total 100.

^b^ Includes motor, social communication, and learning difficulties.

^c^ One participant in the MAU group had missing data.

^d^ Scores range from 0 to 10, with higher scores indicating greater severity of distress and impairment.

^e^ Scores range from 0 to 114, with higher scores indicating greater severity of anxiety.

^f^ Scores range from 0 to 68, with higher scores indicating greater severity of depressive symptoms.

^g^ Scores range from 0 to 150, with higher scores indicating more functional impairment.

^h^ Indicates percentage of school days in the last 4 weeks (range, 0-100).

^i^ Determined using the health-related quality of life, with 5 dimensions, of which we used the Physical Well-being and Psychological Well-being scales.

^j^ Scores range from 36 to 252, with higher scores indicating greater intensity of behavioral problems.

^k^ Scores range from 0 to 36, with higher scores indicating more behavioral problems.

^l^ Scores range from 0 to 40, with higher scores indicating greater severity of general psychopathology.

Follow-up data for all outcomes were available by August 28, 2019. The 197 youths allocated to MMM received a mean (SD) 11.0 (2.6) therapy sessions (range, 0-13), and 175 (88.8%) received a full dose of 9 to 13 sessions. There were no significant differences in the proportion of youths who received 9 to 13 sessions among the 3 top-problem groups (104 [91.2%] of 114 with anxiety, 27 [87.1%] of 31 with depressive symptoms, and 44 [84.6%] of 52 with behavioral problems; *P* = .43) and among the 4 municipalities (34 [91.9%] of 37 in Helsingør, 50 [86.2%] of 58 in Holstebro, 51 [87.9%] of 58 in Næstved, and 40 [90.9%] of 44 in Vordingborg; *P* = .80). The therapy was provided at school or an office nearby. A post hoc examination of the correlation between the number of sessions and the primary outcome showed a significant correlation (Spearman correlation coefficient, −0.151; *P* = .045 [n = 177]), suggesting an association between number of sessions and reduction of SDQ Impact scores (eFigure 4 in Supplement 2). The 199 youths allocated to MAU received a mean (SD) of 1.6 (0.6) coordinating visits (range, 0-2; 198 received ≥1). By week 18, 35 MAU participants (17.6%) received individual therapy, group therapy, and/or parental training in the municipalities. None of the 396 trial participants withdrew consent; 344 participants (177 [89.8%] in MMM vs 167 [83.9%] in MAU; *P* = .08) completed the primary outcome assessment at week 18.

### Outcomes

#### Primary Outcome

**Figure 2.**
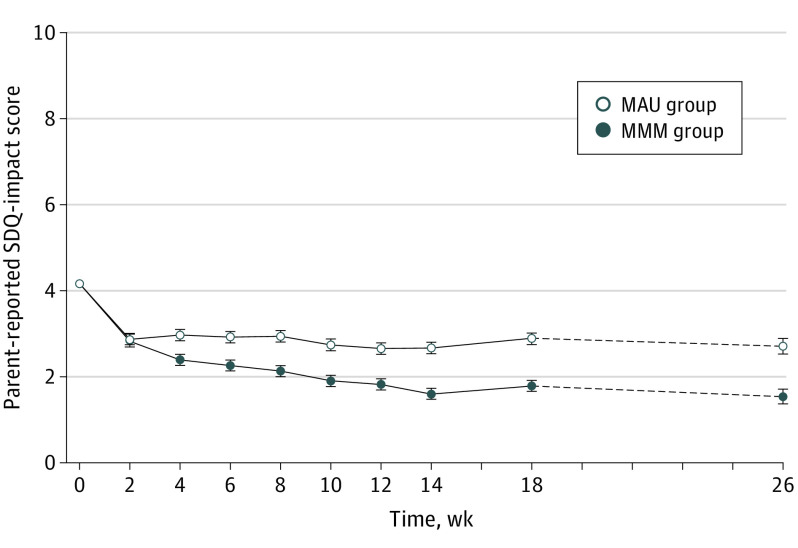
Change From Baseline in Impact of Child’s Mental Health Problems Trajectory of least-squares mean scores over time for the impact of the child’s mental health problems reported by the parent (Strengths and Difficulties Questionnaire [SDQ] Impact scale score) from baseline to week 18, plus the extended follow-up to week 26. Least-squares mean estimates were calculated from repeated mixed-measure models for data at 0 to 18 weeks and from analysis of covariance models for data at 26 weeks. The analyses were based on data from the intention-to-treat population. For each group, the error bars indicate the standard errors. MAU indicates management as usual; MMM, Mind My Mind intervention.

The impact of problems, measured by the parent-reported SDQ Impact scale, improved by −2.34 from 4.12 points for MMM vs −1.23 from 4.21 points for MAU (difference between groups, 1.10; 95% CI, 0.75-1.45; *P* < .001; Cohen *d* = 0.60) (Figure 2 and Table 2). Exploratory subgroup analyses showed no moderating effects of age or geographical region on the primary outcome (eTable 10 in Supplement 2).

**Table 2.** Change From Baseline in Primary and Key Secondary Outcomes at 18 Weeks (ITT Population)^a^

Outcome measure	MMM group		MAU group		Difference	*P* value	Effect size
	No. of participants	Measure	No. of participants	Measure			
Primary							
SDQ Impact scale score (parent-reported)^b^	197	–2.34 (0.13)	198	–1.23 (0.13)	–1.10 (–1.45 to –0.75)	<.001	–0.60
Key secondary							
Anxiety, SCAS score (parent-reported)^c^	197	–6.24 (0.66)	199	–1.34 (0.67)	–4.90 (–6.68 to –3.12)	<.001	–0.52
Depressive symptoms, MFQ score (parent-reported)^d^	197	–5.82 (0.48)	199	–2.72 (0.49)	–3.10 (–4.40 to –1.81)	<.001	–0.45
Level of daily functioning, WFIRS score (parent-reported)^e^	197	–7.56 (0.62)	199	–2.78 (0.64)	–4.78 (–6.47 to –3.10)	<.001	–0.54
School attendance (parent-reported)^f^	197	0.03 (0.01)	199	0.00 (0.01)	0.03 (0.01 to 0.06)	.009	0.26
Top-problem score (parent-reported)	197	–3.08 (0.14)	199	–1.37 (0.14)	–1.71 (–2.08 to –1.33)	<.001	–0.87
KIDSCREEN-27 score, mean (SD), *t* value (self-reported)^g^							
Physical Well-being scale	197	3.08 (0.54)	199	2.56 (0.59)	0.52 (–1.01 to 2.04)	.51	0.06
Psychological Well-being scale	197	2.74 (0.49)	199	1.03 (0.54)	1.71 (0.33 to 3.09)	NC	0.24
Behavioral problems, ECBI (parent-reported)							
Intensity score^h^	197	–13.68 (0.97)	199	–6.47 (1.00)	–7.20 (–9.84 to –4.56)	NC	–0.52
Problem score^i^	197	–3.62 (0.27)	199	–2.30 (0.28)	–1.32 (–2.05 to –0.59)	NC	–0.34
Emotional and behavioral problems, SDQ Total Difficulties score (parent-reported)^j^	197	–4.07 (0.24)	198	–1.93 (0.25)	–2.14 (–2.79 to –1.48)	NC	–0.62
Responder indices, No. (%) (parent-reported)							
SDQ Impact scale score ≥1-point reduction from baseline	197	144 (73.1)	199	93 (46.7)	3.15 (2.06 to 4.81)	NC	1.58
SDQ scores below inclusion cutoff^k^	197	98 (49.7)	199	56 (28.1)	2.59 (1.70 to 3.95)	NC	1.80

Abbreviations: ECBI, Eyberg Child Behavior Inventory; ITT, intention-to-treat; MAU, management as usual; MFQ, Mood and Feelings Questionnaire; MMM, Mind My Mind; NC, not calculated; SCAS, Spence Children’s Anxiety Scale; SDQ, Strengths and Difficulties Questionnaire; WFIRS, Weiss Functional Impairment Rating Scale.

^a^ Data presented as least-squares means (with standard error) unless otherwise stated. The differences between groups are the difference in least-squares means (95% CI) for the continuous outcomes and odds ratios (95% CI) for the dichotomous outcomes. The effect sizes are standardized mean differences for continuous outcomes and risk ratios for dichotomous outcomes.

^b^ Scores range from 0 to 10, with higher scores indicating greater severity of distress and impairment.

^c^ Scores range from 0 to 114, with higher scores indicating greater severity of anxiety.

^d^ Scores range from 0 to 68, with higher scores indicating greater severity of depressive symptoms.

^e^ Scores range from 0 to 150, with higher scores indicating more functional impairment.

^f^ Indicates percentage of school days in the last 4 weeks (range, 0-100).

^g^ Determined using health-related quality of life with 5 dimensions, of which we used the Physical Well-being and Psychological Well-being scales.

^h^ Scores range from 36 to 252, with higher scores indicating greater intensity of behavioral problems.

^i^ Scores range from 0 to 36, with higher scores indicating more behavioral problems.

^j^ Scores range from 0 to 40, with higher scores indicating greater severity of general psychopathology.

^k^ Indicates parent-reported SDQ Total Difficulties score of at least 14, Emotional Problems score of at least 5, and/or Conduct Problems score of at least 3, combined with an SDQ Impact score of at least 1.

#### Secondary Outcomes

The MMM intervention was evidently superior to MAU for anxiety, depressive symptoms, daily functioning, school attendance, and the top-problem score (Table 2). Child-reported physical well-being showed no significant difference between groups (0.52; 95% CI, −1.01 to 2.04; *P* = .51), and owing to the gatekeeping rule (hierarchical testing), the remaining key secondary outcomes are reported without claiming inferential significance. All effects of MMM vs MAU were maintained at week 26 except for school attendance (effect size, 0.22; *P* = .05) (Table 3). The primary and the secondary outcomes covaried weakly or moderately (range, −0.030 to 0.585 in MMM and −0.096 to 0.581 in MAU), except for strong correlations between the 2 measures of behavioral problems (0.719 in MMM and 0.634 in MAU), indicating that the secondary outcomes added value to the primary outcome (eTables 8 and 9 in Supplement 2). Responder and remitter status were significantly higher for MMM vs MAU, including parent-reported responses (144 of 197 [73.1%] vs 93 of 199 [46.7%]; NNT, 4; 95% CI, 3-6) and parent-reported remissions (98 of 197 [49.7%] vs 56 of 199 [28.1%]; NNT, 5; 95% CI, 3-9).

**Table 3.** Exploratory Outcomes: Change From Baseline at 26 Weeks (ITT Population)^a^

Outcome measure	MMM group		MAU group		Difference	Effect size
	No. of participants	Measure	No. of participants	Measure		
Primary						
SDQ Impact scale score (parent-reported)^b^	179	–2.54 (2.31)	157	–1.34 (2.69)	–1.20 (–1.74 to –0.66)	−0.48
Key secondary						
Anxiety, SCAS score (parent-reported)^c^	178	–8.19 (13.43)	157	–1.37 (12.62)	–6.82 (–9.63 to –4.01)	−0.52
Depressive symptoms, MFQ score (parent-reported)^d^	178	–8.01 (10.03)	156	–3.58 (10.67)	–4.43 (–6.66 to –2.20)	−0.43
Level of daily functioning, WFIRS score (parent-reported)^e^	178	–9.12 (13.78)	155	–4.08 (12.56)	–5.04 (–7.90 to –2.18)	−0.38
School attendance (parent-reported)^f^	170	0.06 (0.23)	148	0.01 (0.18)	0.04 (–0.00 to 0.09)	0.22
Top-problem score (parent-reported)	179	–3.30 (2.49)	158	–1.80 (2.39)	–1.50 (–2.03 to –0.98)	−0.62
KIDSCREEN-27 score, mean (SD), *t* value (self-reported)^g^						
Physical Well-being scale	172	4.45 (10.49)	140	3.52 (10.17)	0.92 (–1.39 to 3.24)	0.09
Psychological Well-being scale	173	4.96 (9.64)	140	2.55 (10.37)	2.41 (0.18 to 4.64)	0.24
Behavioral problems, ECBI (parent-reported)						
Intensity score^h^	178	–18.04 (22.78)	155	–9.37 (19.53)	–8.67 (–13.28 to –4.06)	−0.41
Problem score^i^	178	–4.95 (6.11)	155	–2.86 (4.98)	–2.09 (–3.29 to –0.89)	−0.37
Emotional and behavioral problems, SDQ Total Difficulties score (parent-reported)^j^	179	–5.20 (5.23)	157	–2.73 (5.27)	–2.47 (–3.60 to –1.34)	−0.47
Responder indices, No. (%) (parent-reported)						
SDQ Impact scale score ≥1-point reduction from baseline	197	147 (75)	199	97 (49)	1.53 (1.30 to 1.80)	1.53
SDQ scores below inclusion cutoff^k^	197	115 (58)	199	62 (31)	1.87 (1.48 to 2.38)	1.87

Abbreviations: ECBI, Eyberg Child Behavior Inventory; ITT, intention-to-treat; MAU, management as usual; MFQ, Mood and Feelings Questionnaire; MMM, Mind My Mind; SCAS, Spence Children’s Anxiety Scale; SDQ, Strengths and Difficulties Questionnaire; WFIRS, Weiss Functional Impairment Rating Scale.

^a^ Data are presented as mean (SD) unless otherwise stated. Differences between groups are unadjusted differences in mean changes with corresponding 95% CI for continuous outcomes; for dichotomous outcomes, differences are estimated as unadjusted risk ratios with corresponding 95% CI. Missing data are conservatively assumed to be nonresponders. Effect sizes are calculated as Cohen standardized mean difference for continuous outcomes and risk ratios for dichotomous outcomes. The group difference and effect size are identical for dichotomous outcomes (ie, both reported as risk ratios).

^b^ Scores range from 0 to 10, with higher scores indicating greater severity of distress and impairment.

^c^ Scores range from 0 to 114, with higher scores indicating greater severity of anxiety.

^d^ Scores range from 0 to 68, with higher scores indicating greater severity of depressive symptoms.

^e^ Scores range from 0 to 150, with higher scores indicating greater functional impairment.

^f^ Indicates percentage of school days in the last 4 weeks (range, 0-100).

^g^ Determined using health-related quality of life with 5 dimensions, of which we used the Physical Well-being and Psychological Well-being scales

^h^ Scores range from 36 to 252, with higher scores indicating greater intensity of behavioral problems.

^i^ Scores range from 0 to 36, with higher scores indicating more behavioral problems.

^j^ Scores range from 0 to 40, with higher scores indicating greater severity of general psychopathology.

^k^ Indicates parent-reported SDQ Total Difficulties score of at least 14, Emotional Problems score of at least 5, and/or Conduct Problems score of at least 3, combined with an Impact scale score of at least 1.

### Sensitivity Analyses

The inferences from all primary and key secondary outcomes of MMM vs MAU were robust, and the estimates were largely unchanged when multiple imputation was used at weeks 18 (eTable 3 in Supplement 2) and 26, except for child-reported psychological well-being (eTable 4 in Supplement 2). Likewise, all the results were robust when analyzing only participants with complete data (eTable 5 in Supplement 2) or when the trial population was split by date of trial registration (eTables 6 and 7 in Supplement 2).

### Harms

The proportion of participants with increased suicidal ideations or negative cognitions was similar in the MMM vs MAU groups by week 18 (20 of 151 [13.2%] vs 22 of 121 [18%.2]; *P* = .26) but lower by week 26 (7 of 150 [4.7%] vs 20 of 120 [16.7%]; *P* = .003). A small proportion of youth (20 of 173 [11.6%] vs 12 of 142 [8.5%] at week 18; 17 of 173 [9.8%] vs 14 of 140 [10.0%] at week 26) reported deterioration in the quality of their family relationships, free time, and friendships, apparently without group differences (eTable 1 in Supplement 2). No clinician reports of adverse effects were received during the trial, and all participants were alive beyond August 31, 2019.

### Other Outcomes

The results of the exploratory outcomes supported the primary and secondary analyses, suggesting a broad range of benefits of MMM vs MAU, including reduced impact of problems in school (teacher-reported SDQ, range, 0-6), reduced parental stress in role functioning (Parental Stress Scale), and improved Experience of Service Questionnaire scores (eTable 2 in Supplement 2). The exploratory, age-restricted self-reported outcomes showed beneficial changes in the same direction as in the parent-reported outcomes, except for quality of family relations, free time, and friendships at week 18 (data overlapping with measures of potential harm), indicating nonsignificant intervention group differences in the opposite direction.

## Discussion

Timely and effective interventions are urgently needed to meet the global burden of mental health disorders among youth.^1,36^ Despite existence of effective treatments, most youth with emotional/behavioral problems do not receive them.^13^ Barriers include lack of scalable programs and infrastructures for successful implementation of quality care.^37^

Transdiagnostic approaches may facilitate the large-scale dissemination of evidence-based treatments for youth mental health problems.^38^ In our pragmatic RCT, the transdiagnostic, modular MMM was significantly superior to MAU in the primary outcome of parent-reported impact of problems at the end of treatment (week 18), with a clinically meaningful between-group difference^22^ and a medium Cohen effect size of 0.60. Results were robust and consistent across several prespecified sensitivity analyses. Furthermore, most secondary and exploratory outcomes, including improvement in parent-reported youth anxieties, depressive symptoms, daily and social functioning, school attendance, and top-problem scores, showed similar superiority of MMM, as did teacher-reported impact of problems. Response (NNT, 4) and remission (NNT, 5) were significantly more likely with MMM, and most of the favorable results were sustained until week 26. Finally, levels of harms were low and nondifferential by the end of treatment but were significantly lower with MMM vs MAU by week 26. The lack of effect on self-reported physical health suggests specificity of benefits for psychological mental health.

The NNT of 4 for response compares well with the estimated NNT of 3 for anxiety-specific CBT vs a wait list control condition. The weak or moderate correlations of the primary and secondary outcomes confirmed that these measures mapped into distinct yet partly overlapping outcome domains.

Our results indicate real-life benefits of the implementation of a transdiagnostic and modular CBT in a nonspecialist setting under the hallmark conditions of a pragmatic trial. Of note is an important treatment-development difference between MMM and MATCH, which has shown benefits in the Child STEPs program.^15,18^ The MMM intervention was developed for use in nonspecialized, school-based care settings and MATCH, for use in community mental health care settings. Moreover, the psychologists in MMM had limited prior experience with CBT and manualized psychotherapy, and yet the help-seeking individuals presented with anxiety, depressive symptoms, and/or behavioral problems above the diagnostic threshold in most of the cases.

The beneficial effects of the transdiagnostic approach in MMM may depend on the implementation model, which involved an infrastructure for managing self-referrals, visitation, monitoring of activities and outcomes, user feedback for personalized treatment, video-recorded sessions, and online supervision. Shared infrastructure has been identified as a key to coordinating community-based youth services.^39^ Our results contrasted the null effects in a recent pragmatic RCT of transdiagnostic, modular CBT vs usual care^18^ using low-intensity supervision.

### Strengths and Limitations

Our study has several strengths, including a large sample size; standardized assessments; low attrition; parent-, teacher-, and child-reported outcomes; blinded evaluators; and intention-to-treat analyses. Limitations include nonvalidated measures of harms, age restrictions for child-reported outcomes, and no blinding of participants and therapists. The parents reported limited treatment activity in the MAU group, reflecting real-world treatment conditions.

## Conclusions

This pragmatic RCT demonstrated superiority of MMM vs MAU in the community, supporting the large-scale dissemination of flexible, modular CBT programs in nonspecialist settings for youth with common emotional and/or behavioral problems. Future research should establish medium- and long-term benefits and costs of the MMM model and include clinician observations.

## Notes

Supplement 1. **Trial Protocol**

Supplement 2. **eMethods 1.** Steering Committee **eMethods 2.** Literature Overview **eMethods 3.** Interventions in Mind My Mind (MMM) **eMethods 4.** Management as Usual (MAU) **eFigure 1.** The Prototypical Program for Anxiety in MMM **eFigure 2.** The Prototypical Program for Depressive Symptoms in MMM **eFigure 3.** The Prototypical Program for Parent Training in MMM **eFigure 4.** Scatterplot of the Correlation Between the Number of Sessions and the Primary Outcome Measure at 18 weeks, in the MMM Intervention Group (Observed Values) **eTable 1.** Potential Harms and Negative Outcomes at 18 Weeks and at 26 Weeks (the ITT Population) **eTable 2.** Other Exploratory Outcomes: Change from Baseline at 18 Weeks and 26 Weeks (the ITT Population) **eTable 3.** Change From Baseline in Primary and Secondary Outcomes at 18 Weeks in the ITT Population: Missing Data Handled Using Multiple Imputation **eTable 4.** Exploratory Outcomes: Change from Baseline at 26 Weeks in The ITT Population: Missing Data Handled Using Multiple Imputation **eTable 5.** Change From Baseline in Primary and Secondary Outcomes at 18 Weeks: Per Protocol Population **eTable 6.** Change From Baseline in Primary and Secondary Outcomes at 18 Weeks: Before Trial Registration **eTable 7.** Change From Baseline in Primary and Secondary Outcomes at 18 Weeks: After Trial Registration **eTable 8.** Correlation Matrix Exploring the Intercorrelations of the Primary and Key Secondary Outcomes (Change Scores) in the MMM Group **eTable 9.** Correlation Matrix Exploring the Intercorrelations of the Primary and Key Secondary Outcomes (Change Scores) in the MAU Group **eTable 10.** Subgroup Analysis for the Observed Parent-Reported SDQ Impact Scores (n = 343) **eReferences.**

Supplement 3. **Data Sharing Statement**
