# Supplementary material for: Effectiveness of Transdiagnostic Cognitive-Behavioral Psychotherapy Compared With Management as Usual for Youth With Common Mental Health Problems: A Randomized Clinical Trial
Source: JAMA Psychiatry. 2020 Dec 23;78(3):1–12. doi: 10.1001/jamapsychiatry.2020.4045 (PMC7758821; doi:10.1001/jamapsychiatry.2020.4045)
Supplement: Supplement 1. — Trial Protocol [file jamapsychiatry-e204045-s001.pdf]

Pia Jeppesen

Protocol version 1.2, 2017-18-04

PROTOCOL FOR EFFECTIVENESS RCT

Randomized trial of a transdiagnostic, cognitive and behavioral intervention versus treatment as usual in school-aged children with emotional and behavioral disturbances:

The Mind My Mind study

Dansk navn for forsøget til brug for deltagere og samarbejdspartnere:

Afprøvning af psykologisk hjælp til børn og unge i skolealderen med tegn på angst, depression og/eller adfærdsvanskeligheder:

Mind My Mind-forsøget

CONTENT

Protocol for effectiveness RCT 1
The Mind My Mind investigators and sponsors 4
Principal investigator 4
Sponsors 4
Trial sites 4
The research group 5
Advisory board 5
Researchers 5
Authors and expert supervisors of the Mind My Mind manual 6
Introduction to the Mind My Mind study 7
Background and rationale 7
Objectives of the Mind My Mind project 8
The present study of effectiveness 8
The associated sub-studies 10
The Mind My Mind pilot phase 10
Lessons learned from the pilot RCT 11
Review of The evidence 13
Search strategy 13
Anxiety 13
Depression 14
Behavioral disturbances 16
Transdiagnostic programs 17
Summary of the evidence 17
Methods, materials and research plan 18
Design 18
Population and trial sites 18
Inclusion and exclusion criteria 19
Recruitment and visitation procedures 20
Inclusion and baseline asessment 23
Randomized allocation to intervention 23
Experimental intervention 24
Problem-specific module sequences 25
Caseload 27

Supervision of the Mind My Mind trainers 27
Education of trainers and supervisors 28
Ongoing video-based supervision 28
Program fidelity and therapist competencies 29
Treatment as usual 29
Deterioration during the trial 30
Assessments of outcome 30
Flow diagram 31
Trainer's Records of each session 34
Standardized psychometric interviews and questionnaires 34
Measurement of potential harms 37
The primary measure of potential harms 38
The secondary measures of potential harms 39
Plan for the Statistical analyses 39
The primary hypothesis 40
The secondary hypotheses and outcomes 40
The hypotheses of potential harms 41
Missing values 42
Other statistical analyses 42
Sample size estimation 42
Potential harmful effects, risks and inconveniences 43
User involvement 43
Participants' integrity and privacy 44
Notifying The Danish Data Protection Agency of the Databases 44
Databases and data security 44
Financial support 45
Financial compensation for participation in research assessments 45
Informed consent 45
Participants' Access to information on the trial 46
Publication of study results 47
Ethical considerations 47
Clinical and scientific perspectives 47
Participants' Insurance 48

THE MIND MY MIND INVESTIGATORS AND SPONSORS

PRINCIPAL INVESTIGATOR

Pia Jeppesen, Ph.D., Associate Professor, Institute for Clinical Medicine, Faculty of Health and Medical Sciences, University of Copenhagen, Denmark; Specialist in Child and Adolescent Psychiatry and Senior Researcher, Child and Adolescent Mental Health Centre, Mental Health Services of the Capital Region of Denmark.

Address: Nordre Ringvej 69, DK-2600 Glostrup, Copenhagen Denmark.
E-mail: Pia.jeppesen@regionh.dk.

Psykiatrifonden and the Child and Adolescent Mental Health Centre have signed a contract that establishes the principal investigator's (PI's) role in relation to the project and independence from the project's owner and sponsor.

SPONSORS

This private health science research project is sponsored by Psykiatrifonden (Hejrevej 43, 2400 Copenhagen NV, business registration number [CVR]: 19174883) and TrygFonden (Hummeltoftevej 49, 2830 Virum, CVR: 10430410).

TrygFonden funded the project through a grant for Psykiatrifonden.

The Project manager is Birthe Wielandt Houe for Psykiatrifonden.

TRIAL SITES

The experimental intervention will be tested in two municipalities in the Zealand Region (Vordingborg and Næstved) and one municipality in the Central Denmark Region (Holstebro).

The trial is implemented in the context of Denmark's Educational Psychological Counseling (PPR) services, which are organized as part of the Danish education sector and is available in every municipality. PPR's counseling includes advice to teachers, pedagogues and parents concerning child development and special education for use in nurseries, kindergartens, schools and other educational settings. Educational psychologists from PPR also work with individual children who have special needs. This sometimes includes clinical assessments intended to ensure that the environment meets children's needs. Some municipalities also offer free and open interventions on psychological matters for groups or individuals.

THE RESEARCH GROUP

ADVISORY BOARD

Kerstin Plessen, Ph.D., Professor and Head of Research, Child and Adolescent Mental Health Centre, Mental Health Services of the Capital Region of Denmark; Professor, Institute for Clinical Medicine, Faculty of Health and Medical Sciences, University of Copenhagen, Denmark.

Niels Bilenberg, Ph.D., Professor and Head of Research, Department for Child and Adolescent Psychiatry, Psychiatry in Southern Denmark and University of Southern Denmark.

Per Hove Thomsen, Dr. Med., Professor and Director of the Research Center at the Psychiatric Hospital for Children and Adolescents, Aarhus University Hospital, Denmark.

Mikael Thastum, Ph.D., Professor, Department of Psychology, Aarhus University, Denmark. He is the PI for several studies of the efficacy and implementation of CBT programs for children with anxiety.

Simon-Peter Neumer, Ph.D., Senior Researcher, Centre for Child and Adolescent Mental Health, Oslo, Norway. He is the principal investigator for several large intervention projects aiming to promote Children's Mental Health in Norway and is trained in psychology, psychiatric epidemiology and public health.

Wendy Silverman, Ph.D., Alfred A. Messer Professor of Child Psychiatry, Professor of Psychology and Director, Yale Child Study Center Program for Anxiety Disorders, USA. She has published five books and many scientific papers and chapters in the area of child and adolescent anxiety disorders. As the PI for National Institute of Mental Health research studies, she has sought for the past two decades to develop and evaluate treatments for anxiety disorders in children and adolescents.

Christoph U. Correll, Professor of Psychiatry and Molecular Medicine, Hofstra Northwell School of Medicine; Medical Director, Recognition and Prevention program, Zucker Hillside Hospital, New York, USA. He is a specialist in general psychiatry and in child and adolescent psychiatry. His research and clinical work focus on the early identification, characterization and treatment of psychiatric disorders.

RESEARCHERS

Rasmus Trap Wolf, M.Sc., Health Economist and Project Manager, Danish Institute for Local and Regional Government Research; applicant for the doctoral position (Ph.D. student) affiliated with the MMM study.

Professor Dorte Gyrd-Hansen, University of Southern Denmark, Department of Business and Economics & Department of Public Health, will be the faculty supervisor for Rasmus Trap Wolf.

Furthermore, the research team will include:

1) A postdoctoral research position for an experienced psychologist/psychiatrist with extensive CBT experience. This postdoctoral researcher will be responsible for the associated sub-study on the development and evaluation of treatment fidelity measures.
2) A postdoctoral statistician to be affiliated with the Research Unit at the Child and Adolescent Mental Health Center in the Capital Region of Denmark and the biostatistical department at Copenhagen University. This researcher will perform the statistical analyses planned for the

Mind My Mind RCT and associated sub-studies under supervision from the biostatistical department (Professor Claus Thorn Ekstrøm).

3) A psychologist/medical doctor will be employed as research assistant for two years.

All senior researchers (Pia Jeppesen, the statistician, and the postdoctoral psychologist/psychiatrist) will be located in the Research Unit at Child and Adolescent Mental Health Center, Mental Health Services of the Capital Region of Denmark. Ramus Wolf Trap will be affiliated with the research unit as well as the Danish Institute for Local and Regional Government Research and the University of Southern Denmark.

The research unit at the Child and Adolescent Mental Health Center is headed by Professor Kerstin Plessen, Faculty of Health and Medical Sciences, University of Copenhagen, Denmark. Research in child and adolescent mental health is a focus area in the Mental Health Services of the Capital Region of Denmark. The Research Unit of the Child and Adolescent Mental Health Center conducts epidemiological, clinical and translational research in child psychiatry with the overall aim to promote the best clinical and preventive practice. Several prospective longitudinal intervention studies investigate the effects of interventions and the risk and resilience processes driving the development of mental disorders in children and adolescents and influencing the response to treatment. The research Unit currently has 30 employees and multiple collaborations with other national and international research institutes.

The Mind My Mind research team will be headed by the PI. The Mind My Mind researchers will work together as a group in the research unit in Glostrup. The department provides assistance from technical administrative personal and access to a kitchen, a meeting room, IT support etc. Each of the researchers will get their own office next door or very close to the PI. There will be daily collaboration and weekly meetings to share and address scientific challenges, and help each other with data collection and other logistical tasks.

The PI (Pia Jeppesen) has extensive experience in clinical and epidemiological psychiatric research, including complex trials. Her publication list includes four papers as first author and six papers as last author (including two shared last authorships) in high-ranking psychiatric journals. H-index = 17.

The project manager (Birthe Wielandt Houe) successfully headed the Mind My Mind pilot study and is an experienced leader who has held leading positions in the regional health care sector as head of planning division and head of administration in regional hospitals. These positions include areas, such as collaborations with municipalities and GPs on various aspects.

AUTHORS AND EXPERT SUPERVISORS OF THE MIND MY MIND MANUAL

Lisbeth Jørgensen, M.Sc., Authorized Psychologist and Specialist in Psychotherapy; coauthor of the Mind My Mind manual, responsible for the sections on depression and general competencies; coauthor of the Mind My Mind supervision manual; clinical psychologist in private practice; Consultant, Anxiety Disorder Clinic for Children and Adolescents, and Lecturer, Department of Psychology, Aarhus University, Denmark.

Christina Magni Kjerholt, M.Sc., Psychologist; coauthor of the Mind My Mind manual, responsible for the sections on behavioral problems; coauthor of the Mind My Mind supervision manual; clinical psychologist in private practice; Parent Management Training – Oregon model (PMTO) mentor;

coauthor of the Danish PMTO manuals; leading trainer of PMTO therapists in Denmark; supervisor in PMTO and Mind My Mind.

Kristian Bech Arendt, Ph.D., M.Sc., Psychologist; coauthor of the Mind My Mind manual, responsible for the sections on anxiety and trauma; Clinical Psychologist and Researcher, Anxiety Disorder Clinic for Children and Adolescents, Aarhus University, Denmark.

INTRODUCTION TO THE MIND MY MIND STUDY

BACKGROUND AND RATIONALE

The systematic review of general population-based studies (1) shows that mental health disorders are common in childhood and that incidence rates increase during adolescence. The evidence suggests that 1 out of 5 individuals are affected by a mental health disorder before the age of 20.

A recent meta-analysis (2) of the prevalence of mental disorders in children and adolescents, based on 41 studies conducted in 27 countries, estimated the pooled prevalence of mental disorders at 13.4% (95%CI: 11.3–15.9%). The most common disorders were anxiety (6.5%), depressive disorders (2.6%), disruptive disorders (5.7%) and attention-deficit/hyperactivity disorder (3.4%).

Emotional and behavioral disturbances in childhood and adolescence are associated with a threefold to fourfold increased risk of mental disorders later in life (3-5); increased risk of psychiatric comorbidity (6), poor physical health, social problems, learning difficulties and parental stress (7); and higher costs of education and health and social care (8). Youth mental health problems and disorders are furthermore associated with a fourfold to fivefold increased risk of attempted suicide (9).

Levels of anxiety (10), depression (11) and behavioral problems (12) below the diagnostic threshold have also been associated with increased risk of subsequent mental disorders and of poor prognosis (11;12).

There is growing evidence regarding the beneficial effects of psychological interventions that use cognitive and behavioral therapeutic methods (cognitive behavioral therapy, CBT) to treat anxiety, depression and behavioral disturbances (see “Overview of Evidence” below), but these evidence-based psychological interventions are usually not accessible, as they are not part of the usual care for children and youths (13).

The well-documented negative consequences of untreated early-life mental health problems point toward the urgent need for the development and implementation of effective programs for early identification and treatment of youths’ emotional and behavioral problems—before the emergence of a manifest mental disorder. The aims are twofold: 1) to treat the problems at hand, and 2) to prevent progression into severe mental health disorders and complex social problems (3;11;14) later in life.

OBJECTIVES OF THE MIND MY MIND PROJECT

Mind My Mind is a large-scale, private and noncommercial health science research project; it is initiated and funded by the Danish foundation TrygFonden, and it is headed by Psykiatrifonden in partnership with TrygFonden. The project is carried out with close collaboration between the project managers and owners, the participating municipalities, and the regional child and adolescent mental health centers.

The overarching goals are to develop, implement, evaluate and disseminate a program for the prevention and treatment of children's emotional and behavioral disturbances at the lowest effective level.

The study has high levels of user involvement in that the participating children and their parents determine the problems that are targeted and provide regular feedback on the child's progress.

In the first stage (2015–16), the Mind My Mind project group developed:

1) a new transdiagnostic modular cognitive and behavioral treatment manual (in Danish) describing the Mind My Mind training;

2) an IT system consisting of Web-based questionnaires for assessing and monitoring children; and

3) an organizational structure, educational content, and the training and ongoing weekly supervision of the PPR psychologists to secure their adherence to and competence in the delivery of the Mind My Mind training.

The ongoing feasibility randomized controlled trial (RCT) demonstrates that the program can be implemented and evaluated using a randomized study design in Danish municipalities.

The aims of the next stage (2017–19) are to implement and evaluate the Mind My Mind program in three municipalities in Denmark.

THE PRESENT STUDY OF EFFECTIVENESS

The Mind My Mind intervention is an indicated prevention and treatment for school-aged children who have clinically significant levels of emotional and/or behavioral problems that impact their daily lives and threaten to disrupt their development but who do not qualify for treatment in the specialized child and adolescent mental health services.

The next crucial step in the evaluation of the Mind My Mind project is to conduct a large RCT that compares the manualized Mind My Mind training (MMM) with treatment as usual (TAU) in children aged 6–16 years who have problems within the domains of anxiety, depressive symptoms and/or behavioral disturbances. Co-occurring trauma-related problems are also targeted in the Mind My Mind training.

The RCT is a pragmatic study of the effectiveness of MMM, as provided by Educational Psychological Counselling (Pædagogisk psykologisk rådgivning, PPR) under "real world conditions." It is a *superiority trial* designed to demonstrate that the MMM is a more effective intervention than TAU in terms of improving the primary and secondary outcome measures.

Anxiety, depressive symptoms and behavioral problems are common in children and adolescents (2); many such youths have comorbid mental disorders (6) and difficulties with friendship, family life and school.

The transdiagnostic and modular MMM aims to improve participants' functioning in daily life by reducing the severity of their anxiety, depressive symptoms and/or behavioral problems, thus *helping solve the impact of problems in the domains of home, school and friendships*. The heterogeneity of the mental health problems in the target group is a huge challenge in terms of outcome measurement, as psychometric scales are either too narrow (e.g., measuring only one dimension of psychopathology) or too broad (e.g., including psychopathologies other than those targeted in Mind My Mind). The psychometric equivalence of problems across the various domains of psychopathology also raises concern. We addressed these challenges by choosing to measure the impact of problems (i.e. the impact of psychopathology) using the impact scale of the Strengths and Difficulties Questionnaire (SDQ) as the primary outcome measure. The SDQ's impact scale measures distress and interference of mental health problems with home life, friendships, classroom learning and leisure activities; it uses multiple informants: the parents, the child (if age 11 or older) and the primary teacher (who is only asked about the child's learning and friendships in school).

The RCT tests the primary hypothesis that the group of children allocated to MMM will experience a significantly greater reduction in the impact of mental health problems when compared with the TAU group, using *the parent-reported SDQ-impact scale as the primary outcome measure*.

Furthermore, we will test hierarchical hypotheses in a fixed sequence (see the statistical analyses plan) to explore group differences in *key secondary outcomes:*

a. Anxiety (SCAS, parent-reported)

b. Depressive symptoms (MFQ, parent reported)

c. Level of daily functioning of the child (WFIRS, parent reported)

d. School attendance (proportion of school-days where the child is present as recorded by the school)

e. Top-problem scores (parent reported)

f. Quality of life (KIDSCREEN, two subscales on physical well-being and psychological well-being, child reported)

g. Behavioral problems (ECBI, parent reported)

h. Emotional and behavioural problems (SDQ, two subscales, parent reported)

The primary hypothesis and the key secondary hypotheses test the group differences at the end of the intervention period (week 18). We will explore all primary and secondary outcomes across all informants (parents, teachers and children) and at both time points (week 18 and 26) (see statistical analyses plan).

The follow-up period

The present RCT will study the costs and effects of MMM relative to those of TAU in the first 26 weeks after allocation to treatment. This is obviously a very restricted observation period when considering the importance of the continued psychological and social development during school age and beyond. If the MMM proves to be superior in the first 26 weeks, a long-term follow-up study will be initiated to answer the question of whether the beneficial effects are maintained after completion of the controlled intervention. Hence, we plan a long-term follow-up of the intervention groups (e.g., two and five years after allocation to treatment). A long-term follow-up study in Denmark is very feasible because we are

able to track the study participants using unique their personal identification numbers and the civil registration. Thus, the participants of the present RCT can be invited by letter or telephone to take part in the follow-up assessments. Furthermore, the unique personal identification numbers enable a linkage between the Danish Psychiatric Central Research Register and other nationwide registers to get information on health and social service use, diagnoses of physical and mental health disorders, prescriptions of medication, education, employment, marital status, household income, and criminality.

When parents give informed consent for their child to participate in the present RCT, they also consent to being contacted for up to two years after inclusion so that they and their child can be informed of the follow-up study. Their active participation in the long-term follow-up study will require a new instance of informed consent.

THE ASSOCIATED SUB-STUDIES

1. Development and evaluation of treatment fidelity measures for modularized cognitive and behavioral intervention for school-aged children with emotional and behavioral disturbances. The specific objectives of this doctoral study are to
   a. conceptualize, define and operationalize the Mind My Mind fidelity measures;
   b. evaluate the psychometric properties of the Mind My Mind fidelity measures;
   c. delineate criteria for the future certification of Mind My Mind therapists;
2. Cost-effectiveness of the Mind My Mind program. This doctoral study aims to
   a. describe the association between SDQ-total difficulties and impact scores for all schoolchildren in one or two municipalities and their individual health care costs, school performance and parents’ labor market situation;
   b. validate the Child Health Utility 9D against the SDQ and the KIDSCREEN (a measure of health-related quality of life). The results will be used to discuss whether CHU9D is suitable for use in cost-utility analysis (CUA) of interventions aiming to improve mental health in children;
   c. conduct an economic evaluation of the Mind My Mind intervention compared to TAU. The specific cost of the Mind My Mind intervention and the TAU in the participating municipalities will be outlined. A cost-effectiveness analysis (CEA) and a CUA comparing the effects of Mind My Mind training versus TAU with the cost of health services, social services and parental cost during the follow up in the Mind My Mind RCT.

THE MIND MY MIND PILOT PHASE

During the pilot phase (2015–17) of the Mind My Mind project, the following tasks were accomplished:

1. Development, pilot testing and revision of the transdiagnostic and modular CBT manual for the treatment of anxiety, depressive symptoms and/or behavioral problems in children aged 6–16 years, including a module for trauma-related problems.

2. Education and training of 4–5 PPR psychologists in each of four municipalities in how to use the MMM manual.

3. Development and implementation of a manual and an organizational structure for the supervision of the local PPR psychologists by experienced psychologists from the regional child and adolescent mental health centers (CAPs). Two supervisors from each CAP were trained in using the manual and supervised by the authors so that they, in turn, could supervise the local PPR psychologist. Each supervisor was responsible for the weekly supervision of 4–5 PPR psychologists conducting the MMM training (one supervisor took care of one municipality).

4. Development of software and platform for the trainer to upload video recordings of all therapy sessions for supervision. Selected videos will be saved and used in research to develop fidelity measures (i.e., measures of therapist adherence and competence in delivery of treatment).

5. Development and implementation of standardized procedures for the assessment of mental health problems in children and youth by use of psychometric questionnaires and interviews with children and parents in each PPR.

6. Development and implementation of a sustainable IT infrastructure for the collection of data in "real time" to monitor the course of each child during visitation and intervention. The IT system automatically sends e-mails and text messages with links to questionnaires (followed by reminders) asking the PPR trainer, child, parent and teacher to report data whenever needed.

7. Inclusion and random allocation (3:1) of 150 children. A total of 113 children were allocated to MMM and 37 children were allocated to TAU. So far, we have follow-up data on 88 (78%) of the 113 children allocated to MMM and 23 (62%) of the 37 children allocated to TAU, but the follow-up is still ongoing and the ultimate drop-out rate will be lower. See appendix 1.

8. The mean number of sessions in the first 91 MMM training courses was 11, and the mean duration of the training course was 13 weeks, as intended.

The success of the ongoing feasibility study demonstrates that this research group is able to conduct a RCT of MMM versus TAU in the context of the PPR in Danish municipalities. The preliminary analyses of MMM and TAU outcomes delivered useful data as inputs for the power calculations in the present study (see section on power calculations). Also, the data on the use of modules, methods and worksheets in the MMM training sessions of the pilot study were used to revise the manual instructions on how to tailor the treatment to the individual child.

LESSONS LEARNED FROM THE PILOT RCT

Based on the successes and failures of the feasibility study, we have improved the methodology in a number of ways:

a) The screening and visitation of children for inclusion in the RCT will now include an extra meeting between the PPR psychologist, the child and the parents in order for them to go over all of the available information, including the standardized assessments, and a brief, highly structured psychopathological interview, to reach a common understanding of the child's problems.

b) The child and parents determine the top problem with help from the PPR psychologist during the visitation.

c) The PPR psychologist classifies the top problem as a principal problem within the domains of anxiety, depressive symptoms, behavioral problems or other type of problem.

d) The PPR psychologist evaluates if the child fulfills each of the inclusion criteria and none of the exclusion criteria for the MMM study. When in doubt, the psychologist must seek advice from the research team before including a child.

e) Children with a prior diagnosis of any developmental or mental disorder (diagnosis based on assessment in the regional child and adolescent psychiatry) will be excluded, regardless of present status or treatment.

f) TAU interventions that are available in each municipality will be listed, and all children/ families who are allocated to TAU will be offered two meetings with a PPR psychologist (or other professional), the first one within two weeks after randomization and the other no later than 17 weeks after randomization. The TAU coordinating PPR psychologist will receive the standardized assessments from visitation and, guided by this information, will recommend the family to seek relevant treatment in the municipality and inform them of how to access such treatment. The TAU comprises the usual care and support in the municipality including educational support by PPR.

g) A thorough documentation of TAU treatments will be added during the course of treatment and follow-up, including both the listed elements and any private treatments.

h) A small financial incentive will be offered to the children for the completion of all study measures at pre, post, and follow-up in accordance with ethical guidelines. The aim is to decrease dropout from assessments, particularly in the TAU group.

i) The schedules for the supervision of trainers are expanded and now include more group-based activities and educational workshops.

j) Therapist competencies and program adherence will be measured based on observations and scorings of video-recorded sessions.

k) The child and parent's ideographic determination and scoring of the top problem will be used as a key secondary outcome to ensure user involvement and relevance of treatment focus.

l) The primary outcome measure will be a standardized and validated measure of the impact of child mental health problems in daily life.

m) The assessments of outcomes are scheduled at fixed time points after the inclusion of the participant, regardless of the intervention to which the individual is allocated and the individual's course during the intervention.

n) The MMM manual is clearly defined as a modular manual with problem-specific modules and suggested sequences of modules. However, the choice, the dose and the sequence may be adapted according to the present needs of child and parents.

REVIEW OF THE EVIDENCE

SEARCH STRATEGY

We conducted a systematic literature search in the databases Ovid MEDLINE, EMBASE and Psych Info with help from a research librarian. The search included RCTs and systematic reviews and meta-analyses of CBT studies of children with either anxiety, depressive symptoms or behavioral disturbances and transdiagnostic CBT studies of children with anxiety and/or depressive symptoms and disorders. At first, we included all studies from the databases up to October 2014. The search was subsequently updated for the period from October 2014 to May 2016.

Because the search identified several thousand publications that had already been included in systematic reviews and meta-analyses, we decided to focus on these reviews and meta-analyses. We used the "revised Amstar checklist" (15;16) to assess the methodological quality of the studies. Most of the reviews in this area have included trials of different cognitive and behavioral intervention programs in different samples and settings. This means that there is a great deal of heterogeneity, and risks of random error cannot be excluded. The following overview of reviews included only systematic reviews and meta-analyses with a sufficient quality score (>25 in Amstar rating).

ANXIETY

The effect of CBT for children and adolescents with anxiety is well documented in several systematic reviews (17-19) and meta-analyses (20-22). Studies show effects of CBT compared to a wait list control, which was measured as the remission of an anxiety diagnosis and symptom reduction after an average of 13 weeks (13 sessions). The latest update of the Cochrane review (22) found significant beneficial effects for CBT versus wait list controls with OR=7.9 (CI 5.3–11.6, P<0.0001) and the number needed to treat to benefit (NNTB) was 6.0 (CI 7.5-4.6) for the primary outcome of the remission of an anxiety diagnosis.

The same meta-analysis (22) found limited and inconclusive evidence regarding the question of whether CBT is more effective than active controls, including TAU and medication. Furthermore, there is no clear evidence indicating that one way of providing CBT is more effective than another (e.g., group format, individually and/or with parents involved) (22). There are indications that CBT programs with more than 9 sessions are more effective (20). The effect does not depend on the child's gender or age (23), and the positive effects of CBT have been found among children as young as 4 years (22).

Comorbidity of depression and conduct disorder is frequent (6) and predicts an overall worse illness course during CBT treatment compared to no comorbidity (24;25), all though CBT does not have lesser effect on anxiety symptoms in children with comorbidity. Some studies have found that both anxiety and depressive symptoms are reduced during CBT for anxiety (25).

Many parents become part of the maladaptive pattern of avoidance behavior (26) when trying to protect their child from unpleasant situations. In doing so, parents may accidentally help to sustain their child's anxiety (26). Despite thorough investigations of the effect of involving parents in their child's CBT treatment (18;27;28), there is no clear evidence that this has significant additive effects.

A meta-analytic study of different types of parental involvement (27) found that the active training of parents in contingency management and the transfer of control to parents were associated with better long-term effects (27). In addition, some individual CBT programs have demonstrated a superior effect

for children of anxious parents when treatment included help for parents to deal with their own anxiety (18). Thus, in spite of the strong rationale for the involvement of parents in their child's CBT, the evidence for an additional effect is still lacking (19), and some authors have speculated that the involvement of parents may dilute the therapy and complicate the therapeutic alliance with the child (28), particularly if parents also present psychopathologies (28). It is possible that certain children and families may benefit from one particular type of parent involvement program, whereas other families may benefit from another type or from no involvement at all. Also, and with few exceptions (29), the question of the direction of influence between child and parent has been overlooked in the treatment studies. Recent review studies have confirmed earlier findings that higher levels of anxiety and comorbidity in children and higher levels of caregiver stress predict poorer outcome (19;30). However, there is inconsistent support for the negative influence of parental psychopathology (31).

There is also limited and inconclusive evidence for the long-term effects of CBT for children and adolescents with anxiety beyond the six-months follow-up (20;22). A recent review (19) demonstrated substantial support for CBT as an effective treatment for child and adolescent anxiety. Most studies have focused on symptom reduction as an outcome, and CBT was the only treatment supported by high-level evidence of an effect on functional impairments (19).

One comparison of studies of CBT programs with and without booster sessions in youths with anxiety and/or depression (32) found that CBT programs with booster sessions were associated with significantly better effects *before and after* the booster sessions, indicating additional effects of scheduling booster sessions.

The most well-known and used CBT programs for the treatment of anxiety disorders in children and adolescents is "Coping Cat" (33), —later developed into a short version (34) named "Friends"—and "Cool Kids" (35). The effect of Cool Kids has been tested in Denmark and demonstrated significant positive effects of group treatment compared to a wait list control (36).

DEPRESSION

The effect of CBT for the prevention and treatment of subclinical and clinical levels of depressive disorders in youths is well documented in several systematic reviews (18;37) and meta-analyses of RCTs and cluster-randomized trials (21;38-41).

The latest Cochrane review and meta-analysis of preventive psychological interventions (38) included 83 studied, of which the majorities were carried out in school-based settings (67 studies) and in targeted populations (53 studies). The psychological interventions included CBT, interpersonal therapy (IPT) and third-wave CBT and were compared with no intervention or with an attention placebo, when available. Most interventions were delivered in a group format. For the primary outcomes of self-rated depressive symptoms and of depression diagnoses at post-intervention and up to 12 months, the preventive interventions had significant positive effects, with NNTB=11, which compares well with other public health interventions (38).

However, as the authors noticed (38), the evidence was of moderate to low quality with a great deal of heterogeneity, and attention placebo controls were lacking, particularly in the comparison of targeted programs. Furthermore, the universal programs that were compared with attention placebo controls showed no effects on depression diagnosis (38). The targeted intervention programs, and particularly those selecting children with depressive symptoms (i.e., indicated prevention), showed overall larger effect sizes. The authors concluded that there was insufficient evidence to support the implementation

of depression prevention programs as standard. More research is needed, and future studies should focus on indicated prevention programs and compare them with a credible control group (38).

The preventive psychological interventions were mainly based on CBT and IPT (38;40), which are therapies with more robust evidence for beneficial effects in the treatment of clinical depression. An exploratory meta-analysis (42) of studies included in the 2011 Cochrane review (40) found interventions of more than eight sessions to be more effective than those of shorter duration. There was insufficient evidence to determine the active components of the CBT programs (42), but those programs with consistent beneficial effects seemed to have a strong focus on cognitive restructuring skills and problem solving (42).

There was evidence of moderate effects (effect size around 0.34) of CBT and other psychological treatments for depressive disorders in youths (18;41) when compared with wait list controls (most studies), active placebos or TAU. Comorbidity of anxiety and depression is frequent and associated with overall poorer outcomes (25). The psychological treatments for depression have demonstrated effects on both depression and anxiety symptoms (25;41), but not on externalizing problems (41). The meta-analysis found consistent beneficial effects for youth' self-reported symptoms, whereas results were mixed and largely non-significant for parents' reported outcome measures (41). The CBT programs fared no better than IPT and other non-cognitive approaches.

Subgroup analyses were applied to identify the active ingredients of treatments (41) and pointed toward greater effects for CBT programs with a stronger focus on behavioral methods relative to the focus on cognitive restructuring. However, CBT programs focusing primarily on cognitive restructuring are far more widespread and better studied. One study (43) found evidence indicating that behavioral activation might be an important ingredient in the treatment of depression in adolescents.

The Cochrane review and meta-analysis (44) of psychological treatment versus antidepressant medication demonstrated a significant reduction in the incidence and severity of suicidal ideation with psychological treatment (mainly CBT) compared to drug treatment (44).

Very few studies have investigated the effect of CBT for children with depressive symptoms below age 13 years, and the interventions under study are mostly school-based group treatments (37). A recent meta-analysis of RCTs of CBT for depressive symptoms in children with a mean age of ≤12 years (45) found 10 very small studies including a total of 267 participants in the intervention group and 256 participants in the comparison group (wait list control or active placebo). The meta-analysis found a significant moderate effect (Cohen's d=0.66) of CBT, and the effects were larger in older studies, among older children and with higher numbers of sessions. Publication bias could not be ruled out. A newer meta-analysis (46) of psychological treatments for depression in the same age group included only seven studies (three of CBT) and, due to lack of RCTs, found inconclusive evidence for the effectiveness of all psychological treatments of depression in pre-adolescent children.

Evidence-based CBT programs for children and adolescents with depression include the program used in the "Treatment of Adolescents with Depression Study" (TADS) (47), the "Primary and Secondary Control Enhancement Therapy" (PASCET) (48;49), group cognitive intervention for preventing depression in adolescent offspring of depressed parents (50;51) and the "Penn Resiliency Program" (52). There are ongoing studies of trans-diagnostic CBT programs for anxiety and/or depression in Norway: The Coping Kids (ClinicalTrials.gov ID NCT02340637) and SMART (ClinicalTrials.gov ID NCT02150265) for children aged 8–12 years. The Norwegian programs (called SMART and Coping Kids) comprise methods from the Coping Cat (33;34) and the ACTION (53) programs. The SMART manual describes 8 group sessions over 8 weeks, and the Coping Kids manual describes 20 group

sessions over 10 weeks in addition to 7 group sessions for parents and 2 individual meetings for children and parents with the therapist.

BEHAVIORAL DISTURBANCES

The evidence-based programs for behavioral problems including violence, aggression and disruptive behavior in children and adolescents can be divided into two: 1) parent training programs aiming to improve parenting practices, communication and contact with children and to train parents in the use of behavioral therapeutic methods in order for them to reinforce the desired behavior in their child; and 2) school-based prevention programs for children and young people, individually or in groups, with and without parental involvement.

Group-based parent training programs for parents of children aged 3–12 years with problem behaviors or conduct disorders (ODD, CD) are the best studied. The Cochrane review and meta-analysis (12) found significant beneficial effects of child behavioral problems (as reported by parents) as well as improvements in parenting practices (measured by an independent observer) (12). There were also small but significant effects on parents' own mental health. The parent training group was compared with wait list; there was no treatment or standard treatment control.

The severity of the child's behavioral problems and the family's socioeconomic status did not moderate the treatment effects (12).

A recent meta-analysis (54) of CBT for externalizing symptoms in children with externalizing disorders (Attention Deficit Hyperactivity Disorder [ADHD] and Oppositional Defiant Disorder [ODD]) included 21 RCTs of CBT programs mostly comprising both parent training and child therapy. The comparison groups were mostly wait list controls or groups that received self-help. The mean age of the children was 7 (SD=2.7) years. The results indicated moderate effects on externalizing symptoms including ADHD, ODD and inattention problems as well as social competence and parental distress. Small reductions of internalizing problems, aggressive behaviors and maternal depressive symptoms were also seen (54). This evidence suggested that CBT for externalizing problems may have beneficial effects on a broad spectrum of symptoms and functioning in both children and parents.

The evidence for school-based prevention programs for children and young people, individually or in groups, with and without the involvement of parents, is also well documented in several reviews and meta-analyses (55-57). The studies showed significant positive effects on behavioral problems as well as on social skills, adaptation and social cognition (55;56). The studies of programs with a strong focus on training in social skills seemed to find greater effects than studies of programs with a strong focus on anger management (57). Moreover, the lengthy and more complex programs showed lesser effects then the brief and more focused programs (55). Some authors found that individual treatment had a greater impact than group treatment (57), whereas others did not (56). It seems to be important that both parents and teachers agree on the need for intervention, as outcomes were significantly worse if only the teacher recognized the child's behavioral problems.

The beneficial effects of parent training programs and programs targeting young people with behavioral problems are well documented (58-60), but age moderated the effects. Parent training is not developmentally appropriate as the primary intervention for adolescents, and the effects of parent training have only been documented for children up to age 13 years (12;60). The effects of programs targeting youths increased with age, suggesting that a certain cognitive maturity helps the child to benefit from training of new behaviors in CBT programs (59).

The most commonly used evidence-based programs for parent training are the Incredible Years (IY) (61), the Oregon Model of Parent Management Training (PMT-O) (62;63) and the Triple-P (64). The evidence-based programs that are directed at children and young people of school age include Problem Solving Skills Training (64-66), Coping Power (67-71) and Alles Kidzzz (72;73). Both IY and PMT-O are used in some of the Danish municipalities but have not been evaluated in an RCT in Denmark.

TRANSDIAGNOSTIC PROGRAMS

The Modular Approach to Therapy for Children with Anxiety, Depression, Trauma or Conduct Disorder (MATCH-ADTC) (74) comprises 33 CBT methods and techniques organized in modules for anxiety, depression, behavioral problems and/or trauma-related problems in children aged 7–13 years. The MATCH manual (75) guides the therapist to choose modules to match the child's current most prominent problem. A 3-armed RCT (76) demonstrated the efficacy of MATCH versus standard problem-specific CBT or TAU. The MATCH group experienced a significantly steeper decline in the severity of problems and symptoms compared with children in the control groups after treatment (76), and the effects were largely maintained after 3 years of follow-ups (77;78). However, there were no significant differences in functioning or in the use of mental health services during the three years of follow-ups. There may be risk of overestimating the effects of MATCH due to use of quasi-randomization, the limited power of a 3-arm comparison and the lack of intention to treat analysis (ITT). There are no other efficacy studies of a similar transdiagnostic, modular CBT program for children and adolescents.

The Unified Protocol for Transdiagnostic Treatment of Emotional Disorders (79;80) has been modified for the individual treatment of youths (81) and for the group treatment of children (82;83) with anxiety and depression, and case studies and open studies have indicated preliminary positive effects. The unified approach involves therapy that focuses on the key underlying cognitive and behavioral processes. This approach tries to improve the child's cognitive flexibility and emotional regulation and to help the child to develop new behaviors instead of maladaptive avoidant behavior.

There is a lack of randomized controlled comparative studies of such unified or generic CBT methods. In addition, the unified manuals have so far not included interventions for behavioral problems.

SUMMARY OF THE EVIDENCE

There is meta-analytical evidence of the beneficial effects of CBT for the prevention and treatment of anxiety (20;22), depression (38;41) and behavioral problems (12;55-57) in children and adolescents. This evidence is based on RCTs in which the experimental intervention was directed against one of these three problem areas. The evidence generalizes to children and youths of both sexes (except that evidence is lacking for the effect of CBT in pre-adolescent children with depression). Though the majority of the studies were conducted in university clinics, the newest and largest studies (22;38) have shown effects for children with sub-threshold problems in the indicated preventive programs delivered at school or in the municipality. The majority of studies are based on self- and parent-reported outcome measures.

There is a lack of data to elucidate the effects of CBT on children's daily functioning, quality of life and satisfaction with treatment. Moreover, we still lack controlled studies of the long-term effects of CBT.

There is clear evidence that booster sessions enhance the effects. The more focused programs with an average of 12–13 sessions are better than long-lasting and complex programs in terms of the prevention and early treatment of anxiety and depression. Individual treatment may be associated with better effects, but the group format is by far the most studied, particularly for younger children. The vast majority of CBT programs involve parents to varying extents, and the fact that everyone gets something perhaps explains why it has been difficult to demonstrate significant effects of parental involvement.

There are two fundamentally different approaches to the development of transdiagnostic CBT programs for children and adolescents: 1) A modular approach comprising problem-specific modules and a flowchart to guide the therapist in how to tailor treatment to the individual child by choosing the right modules; and 2) a unified approach targeting key cognitive and behavioral processes with the aim of improving cognitive flexibility and emotional regulation as well as to reduce avoidance and other maladaptive behaviors.

In both cases, there are more similarities than differences in the fundamental cognitive and behavioral strategies and methods used in the treatment of the three categories of common mental health problems and disorders among children and adolescents.

METHODS, MATERIALS AND RESEARCH PLAN

DESIGN

The Mind My Mind study is a randomized controlled superiority trial (RCT) that compares a modular transdiagnostic cognitive and behavioral treatment program (MMM) with treatment as usual (TAU) in children aged 6–16 years who have emotional and/or behavioral disturbances.

POPULATION AND TRIAL SITES

Children and adolescents aged 6–16 years with emotional and/or behavioral difficulties will be identified in the following study municipalities in Denmark: Vordingborg and Næstved in Region Zealand; and Holstebro in the Central Region of Demark. These three municipalities have agreed to participate in the effectiveness RCT.

The study will include eligible children consecutively. We will begin inclusion on 1. September 2017. As the intervention period should not be interrupted by a long summer holyday, the intake will pause in the months of May, June and July in 2018. Each municipality will sign a contract in which they undertake to include children according to the schedule listed in Table 1.

Table 1: Schedule for inclusion of eligible children in the MMM RCT

	N of municipalities (trial sites)	N of children included at each site	N of children included across Denmark
Sep. - Dec. 2017	3	36	108
Jan - June 2018	3	60	180
Aug. - Oct. 2018	3	40-42	124
**Total**			**412**

The municipalities should be able to reach the required number of included participants before the 15th of October 2018.

The project leaders will collaborate with the local PPR leaders to make sure that educational and health professionals receive written and oral information about the study. The information will be repeated if/when needed in order to identify children with indicated problems.

The PPR psychologists will be educated on use of the MMM manual in week 33 (2017). If the number of trained PPR psychologists drops below 3–4 in any municipality (due to illness, maternity leave or job termination), new PPR psychologists will be offered education in the MMM manual in week 6 (2018).

The number of children included in each municipality will be monitored by the project leaders during the trial. If the intake of children slows down, the project leaders will spread information about the study among parents in the area in order to reach out to help-seeking individuals (e.g., through announcements on local school websites). If the PPR psychologists lack resources to include the required number of children in a given municipality, the project leaders will ask the other participating municipalities to extend their contracts and include more children. The goal is to include a total number of 412 children before 15 of October 2018. That way, the last child included in the study will complete follow-up assessment by the end of April 2019.

INCLUSION AND EXCLUSION CRITERIA

Inclusion criteria

1) Aged 6–16 years and in 0-9th grade (excluding the second semester of the 9th grade).
2) SDQ scores reported by the parent are above the lower cutoff: a total difficulties score of ≥14 and/or emotional problems ≥5, and/or conduct score ≥3; combined with a functional impairment score of ≥1. Scores above this cutoff place the child's difficulties within the top 10 percent of mental health problems in the general age-matched population in Denmark.
3) The child and parents determine one top problem that has to fall within the domains of anxiety, depressive symptoms or behavioral problems, according to the classification by the PPR psychologist.

4) The child and at least one of the two parents understand and speak Danish sufficiently to participate in the treatment.

5) Written informed consent from the holders of the parental rights and responsibilities (usually both parents).

Exclusion criteria

1) Indications that the child may have a severe mental disorder like autism spectrum disorder, ADHD, schizophrenia-like psychosis, an eating disorder, severe obsessive-compulsive disorder, repeated self-harm, abuse or dependence of alcohol or psychoactive drugs or other mental disorder requiring referral to a more intensive assessment or treatment in child and adolescent mental health services (after systematic assessment and according to the usual recommendations and guidelines).

2) Indications of intellectual functional impairment, severe learning difficulties or other special needs that would interfere negatively with the MMM training. The judgment is made as a best estimate by the PPR psychologist on the basis of the available information. A formal intelligence test is not required.

3) A prior diagnosis of any developmental or mental disorder after assessment by the regional child and adolescent psychiatry, regardless of present status or treatment. A prior examination that did not result in a diagnosis of any specific mental health disorder will not exclude the child. The PPR psychologist must consult the PI who decides whether there is sufficient information to exclude the child because of a prior psychiatric history.

4) Prior participation in the MMM pilot or current study.

5) The child and/or parents are unable to participate in weekly sessions throughout the next 13–18 weeks.

The children are not diagnosed before inclusion. After inclusion in the research project, the development and well-being assessment will be administered to the parents and the children (from age 11 and above). A specialist in child and adolescent psychiatry will review the computerized output and the free text answers, and will then set the diagnosis (or diagnoses) of mental health disorders according to the *International Classification of Diseases* (ICD-10) and the *Diagnostic and Statistical Manual of Mental Disorder* (DSM-5). The aim is to be able to compare the sample in the present study with samples included in other studies of CBT for children and adolescents. We expect that the majority of the included children will fulfill the criteria for at least one mental health disorder, although they are below threshold for referral to the regional child and adolescent Psychiatry (CAP).

RECRUITMENT AND VISITATION PROCEDURES

The overall goal of the MMM project is to develop and test an early preventive psychological treatment for school-aged children with common mental health problems. To achieve this goal, it is essential *firstly* to inform the local educational and health professionals about the study and educate them to identify children with early signs and symptoms of emotional and behavioral disturbances, and *secondly* to create a quick and easy pathway to assessment and treatment for children with emotional and behavioral difficulties.

Since the MMM intervention is designed to be open and freely offered to school-aged children and their parents, the participants are by definition self-referred whether or not they are informed by an educational or health professional or motivated by one-self to seek help. Thus, general practitioners and other health professionals cannot formally refer children to MMM, and MMM certainly cannot be used as a "forced measure" for a family. In the case that a child seeks help through MMM, the parents will be notified about taking part in the visitation procedures.

PATHWAYS INTO MIND MY MIND

1) The child and parents read about MMM (e.g., on the school intranet) and seek help at PPR on their own initiative and hence sign up for visitation to take part in the study.
2) The school health care nurse/teacher/educational psychologist/pedagogue or any other professional identifies a child with emotional and/or behavioral difficulties and directs the family to seek help *either* directly at the PPR *or* by contacting their general practitioner.
3) The general practitioner identifies a child with emotional and/or behavioral difficulties and directs the family to seek help at the PPR where they sign up for visitation to take part in the study.

WEB-BASED INITIAL ASSESSMENTS

The visitation for the MMM study will follow a standardized procedure in which PPR psychologists use web-based questionnaires and a clinical interview with the child and parents to assess whether the child has emotional and behavioral difficulties that impact his or her daily life.

The initial screening will be easily accessible for all children aged 6–16 years attending school in 0–9th grade (excluding the second semester of the 9th grade). The first step is simply to contact the local PPR secretary, who will ask the parents to give their contact information, including name, e-mail address and telephone number. Then the parents will receive verbal and written information on the use of web-based questionnaires to be answered by the child and the parents. If the parents return written informed consent to take part in this digitized assessment, their contact information, including their 10-digit personal identification number, are entered into the MMM clinical database. Each case is assigned a unique MMM ID in order to protect participants' personal data and keep them anonymous for researchers (if they are later included in the research study). Once the contact information, including the e-mail addresses and telephone numbers of the parents and the child, are entered into the database, they automatically receive an e-mail and an SMS (short text services on the mobile phone) with a link to the web-based screening questionnaires:

1) SDQ (parent's report, and also a self-report for children aged ≥11)
2) Spence Children's Anxiety Scale (parent's report, and also a self-report for children aged ≥8)
3) Mood and Feelings Questionnaire (parent's report, and also a self-report for children aged ≥8)

The web-based administration of these standardized and validated psychometric questionnaires for the assessment and visitation of children in the PPR is established through cooperation between each trial site and the Mental Health Foundation (Psykiatrifonden). The trial sites will use the web-based questionnaires and the clinical database as a standard procedure for the initial assessment of all help-

seeking children aged 6–16 years whenever they show symptoms or signs of emotional and/or behavioral difficulties. However, children with an obvious need for specialized treatment should be referred to child and adolescent mental health services in accordance with the usual guidelines and with no delay caused by this new procedure.

The municipality is the owner of the local clinical database, and the Mental Health Foundation (Psykiatrifonden) is the data manager of the clinical databases across all trial sites (see the section on database and data security). Each municipality will seek authorization to establish the clinical database by reporting it to the Danish Data Protection Agency according to the Danish law (the law on the processing of personal data). The municipality is also responsible for obtaining informed consent from the parents in order to use the database in the standard visitation procedures, including for the administration of the web-based questionnaires and for administrative purposes.

CLINICAL INTERVIEW TO ASSESS ELIGIBILITY

The child and one or both parents will be invited to this first meeting after having responded to the standardized psychometric instruments. The PPR psychologist will ask structured questions in order to get an understanding of the child's development, family and social situation, school attendance, learning problems, symptoms and functioning in daily life.

The PPR psychologist will then integrate and summarize all of the available information, including the child and parents' reports on the standardized questionnaires, and check with the family to ensure that important information is not missing. The psychologist will also conduct a brief, highly structured psychopathological interview together with the child and parents to reach a common understanding of the child's problems and needs. The PPR psychologist will then guide the child and the parents to collaborate in determining a "top problem" that they would like to address. The psychologist will enter the child's and the parents' ideas about the top problem directly into the clinical database and make sure that the text contains no name or other information that could be used to identify them.

The psychologist will then decide how to classify the top problem within the domains of anxiety, depressive symptoms or behavioral problems. If the top problem falls outside of these domains, it will be classified as "another type of problem," in which case the child will not satisfy the inclusion criteria. The classification of the principal problem is recorded by the PPR psychologist in the database during the meeting. This is important, because the classification is also used as a stratification variable in order to secure balance in the randomly allocated interventions within each subgroup of problems.

The psychologist must finally consider each of the inclusion and exclusion criteria for participation in the MMM study, and, when in doubt, seek advice from the research team. The child is eligible only when the psychologist is able to say "yes" to all inclusion criteria and "no" to all exclusion criteria.

If the child is eligible for inclusion in MMM, the holders of the parental rights and responsibilities (usually both parents) are invited to come for another meeting where they will receive oral and written information about the trial. Young people aged 15–16 will also be invited to the meeting and receive verbal and written information adapted for this age group.

All data collected during the visitation for the MMM trial belongs to the municipality and is recorded in the local clinical database (see the section on database and data security). Parents who consent to participate in the MMM trial also consent to allow all data from the visitation to be used for research.

INCLUSION AND BASELINE ASESSMENT

When the holders of the parental rights (usually both parents) have given informed consent for their child's participation in the trial, the PPR psychologist will tick "yes" or "no" to each of the inclusion and exclusion criteria and then scan and upload the signed consent form as a PDF file to the database.

Then, the database will automatically send an e-mail to notify the research leader that the child awaits approval for inclusion. The research team will check that the psychologist answered "yes" to all of the inclusion criteria and "no" to all of the exclusion criteria and that the consent form is properly signed and dated by the parents (the holders of the parental rights) and the psychologist. If the child satisfies the inclusion criteria and the consent form is properly signed, the research leader will enroll the child in the RCT of MMM versus TAU. Alternatively, the research team will ask the psychologist to correct the data or exclude the child.

Once the child is enrolled, the database will automatically send an e-mail and SMS to both the child and the parents with a link to the web-based baseline questionnaires (see flow diagram) to collect the self- and parent-reported data necessary for research diagnoses and for determining the nature and severity of the child's psychopathology, quality of life and daily functioning in school and in the home. The parents also fill in a questionnaire about their experience of stress as parents (see the list of questionnaires, Table 3). Furthermore, the database will automatically send an e-mail and SMS to the teacher, once the name and contact information is entered by the PPR psychologist/the research team.

The first baseline questionnaire after inclusion and before randomization will be the Development and Well-being Assessment (DAWBA). The child (age 11 years and above) and the parent will automatically receive an e-mail and SMS with the web address, personal user ID and password in order for each of them to log into DAWBA.net that is hosted in the UK.

DAWBA is a package of interviews, questionnaires and rating techniques designed to generate ICD-10 and DSM-5 psychiatric diagnoses for 5–17 year olds. The DAWBA covers the common emotional, behavioral and hyperactivity disorders. The researchers will monitor and prompt the responses on DAWBA. When the parent (and child aged 11 years and above) has responded to the SDQ+DAWBA, the research leader will activate the next step in the data collection. The family will then receive an e-mail and SMS with links to the Mind My Mind web-based questionnaires.

The database will automatically approve the child for randomization when sufficient baseline data have been collected. All standardized questionnaires must be completed by each of the respondents, but the IT system will allow the respondent to skip a maximum of five questions, if the questionnaire has more than 25 items (this general rule does not apply for the SDQ).

RANDOMIZED ALLOCATION TO INTERVENTION

Randomization for MMM training versus the standard treatment and support in the municipality (TAU) is done through centralized randomization, which is provided by Data & Documentation department named DEFACTUM, Social and Health Services and Labour Market, Central Denmark Region. A computer-generated allocation sequence with variable block size will be employed. The principal investigator, the project manager and all researchers and therapists will be blinded to the sequence used. To optimize comparability between the two treatment groups, randomization will be stratified across three factors:

1) The regions (Zealand or Central Denmark)
2) Child's age (6–10 years or 11–16 years)
3) The principal problem (the classification of the Top-problem as 1=anxiety, 2=depressive symptoms or 3=behavioral problems; the mandatory classification is done by the psychologist during the visitation).

Allocation sequence lists will be prepared by DEFACTUM in accordance with the stratification variables. The included children will be randomly allocated 1:1 for MMM training versus TAU. The analyses of the effects of MMM versus TAU will be carried out by members of the research team, who will be blinded to the treatment allocation.

EXPERIMENTAL INTERVENTION

The MMM training is delivered to the individual child by an educational psychologist from the local PPR, with parents being involved to support the child. In the case of a child with behavioral problems, the parents receive training instead of the child and thus become co-agents of changing their child's behavior. The PPR psychologists will receive one week of intensive training and ongoing weekly supervision.

The transdiagnostic MMM manual comprises evidence-based cognitive and behavioral therapy (CBT) methods and techniques, which are organized into unified and disorder-specific modules to target subclinical or clinical levels of anxiety, depression, behavioral disturbances and trauma-related problems. The CBT methods and techniques are well known, as they are adapted from evidence-based programs targeting each of the specific domains of problems in children and adolescents.

The MMM manual describes the typical course and content of training for each of the three problem areas (anxiety, depressive symptoms and behavioral disturbances). Each typical training path is built up as a sequence of modules. A module consists of a collection of consistent methods and techniques. A module usually progresses over several sessions, perhaps in conjunction with other modules. The manual contains flow charts describing the sequencing and dosing of modules within each problem area. This way, the trainer is instructed step by step in aims, methods, techniques and materials while still having the flexibility to tailor the training to the individual child.

Four main paths are described with regard to the content and sequencing of modules:

1. Training of children and adolescents aged 6–16 with anxiety
2. Training of children and adolescents aged 6–16 with depressive symptoms
3. Training of parents of children aged 6–12 with behavioral problems
4. Training of children and adolescents aged 9–16 with behavioral problems (usually combined with parent training)

The problem-specific sequences of modules may be supplemented by modules from other problem areas in case of co-occurring problems and disorders. Furthermore, the module targeting trauma-related problems may be employed at any time if needed.

There are considerable similarities in CBT methods across the domains of psychopathology (anxiety, depressive symptoms and behavioral disturbances), and these shared (unified) methods are therefore described almost identically across the problem-specific training paths.

The problem-specific and unified modules are sequenced so that basic skills are trained first as a foundation for training in more advanced skills. The trainer will repeat and expand the module in two or more sessions depending on how fast the child or parents learn new skills. The trainer may also choose to move on to the next module if lack of progress indicates that the child might benefit more from another approach.

During visitation to take part in MMM, the child and the parents determined a principal problem (the top problem) that they want to address. Upon inclusion they will score the severity of this top problem on a 10-point Likert scale every two weeks. The main goal for the first session is to create motivation for the training by bridging the child's and parents' ideas about the top problem and to help them to set SMART goals for the training (i.e., goals that are specific, measurable, attractive, realistic and timed).

PROBLEM-SPECIFIC MODULE SEQUENCES

1. Training of children and adolescents aged 6–16 with anxiety:
    a. Psychoeducation: awareness and scaling of emotions, introduction of the cognitive circle model.
    b. Cognitive flexibility and restructuring: "detective thinking," probability of the feared event and/or consequences, substituting negative thoughts with helpful thoughts, coping with worry.
    c. Parent session: how to support the child, plan homework and rewards.
    d. Exposures: build a hierarchy of feared situations and plan rewards at each step, plan exposures and prevent avoidance, interceptive and in vivo exposures, work with identification and regulation of emotions.
    e. Problem-solving skills: used only if needed (e.g., to overcome barriers for carrying out the exposures).
    f. Social skills and social cognition: used only if needed; basic skills, taking different perspectives, how to be a good friend, how to manage group pressure.
    g. Review of acquired skills: how to maintain progress and set new goals.

2. Training of children and adolescents aged 6–16 with depressive symptoms:
    a. Psychoeducation: awareness and scaling of emotions, introduction to the cognitive circle model.
    b. Parent session: how to support the child, plan homework and rewards. The parent session is scheduled early in order to help the parents lower expectations and focus on stress reduction and scheduling pleasant activities for their child.
    c. Problem-solving skills: used only if needed (e.g., to overcome barriers for carrying out the behavioral activation).
    d. Behavioral activation: connect feelings and activities, register nourishing and demanding activities, schedule pleasant and nourishing activities, work with thought traps.
    e. Cognitive flexibility and restructuring: "detective thinking," ambiguous situations, substituting negative thoughts with helpful thoughts, how to stop ruminating.

f. Relaxation techniques: optional.
g. Social skills and social cognition: used only if needed; basic skills, taking different perspectives, how to be a good friend, how to manage group pressure.
h. Review of acquired skills: how to maintain progress and set new goals.

3. Training of parents of children aged 6–12 with behavioral problems:
   a. Supporting positive behaviors: how to set clear directions; house rules; use praise, encouragement and incentive systems for contingency management.
   b. Setting limits for the child: set healthy limits, be involved, and monitor child.
   c. Positive family relationships: problem-solving skills, positive communication.
   d. Supporting the parents: boost parents' energy.
   e. Review of acquired skills: how to maintain progress and set new goals.

4. Training of children and adolescents aged 9–16 with behavioral problems (often combined with parent training).
   a. The child's self-image.
   b. Social skills and social cognition: train basic social skills, taking different perspectives, how to be a good friend, how to manage group pressure.
   c. Recognition and regulation of emotions: scaling of emotions, body signals, controlling anger, progressive relaxation.

The manual includes 75 different worksheets to choose from. The trainer hands out the worksheets to be used in the session together with the child and/or the parents. The worksheets are compiled in the participant's private workbook. The workbook will thus contain records of all of the training activities, including homework, rewards, exposures, behavioral activation, social skills training and so on. The trainer will keep a copy of the individual workbook for use in next session, in case the child forgets to bring the workbook.

When relevant, the child's primary teacher will be informed about the child's training by the psychologist or the parents. The teacher may be enrolled in the training by giving support and creating opportunities for the child to try out new skills in the classroom. The teacher also takes part in the evaluation of the child's outcome (see Table 3).

The typical training course will consist of 13 sessions. The last 1–2 sessions should be used to recognize the efforts and the progress made by the participants, to discuss how they can maintain the progress and, if relevant, to set new goals in order to continue the positive development.

The number of training sessions should not exceed 13, and the entire training path should be completed no later than week 17, before the follow-up assessment in week 18. One booster session is offered, and this should be scheduled 6–8 weeks after the end of treatment and no later than week 25; as it should happen before the follow-up assessment in week 26. In cases of children who have minor problems and who respond quickly to training, the MMM training can be limited to 9–13 sessions. If fewer than 9 training sessions are completed, this will be regarded as a protocol violation (lack of compliance).

If the problems being addressed progress during the training, the psychologist must evaluate the problems again and, in collaboration with the family, consider referring the child for more specialized treatment in the municipality or in the CAP.

CASELOAD

The plan is to educate and supervise six PPR psychologists as MMM trainers in each of three municipalities in Denmark, and each psychologist should reach a caseload of 3-5 children per semester.

Table 2: Schedule for training of the children who are randomly allocated to MMM, and the mean caseload for the municipality and the trainers

	N of municipalities (trial sites)	N of MMM trainers in each municipality	Mean caseload per MMM trainer	N of children in MMM training in each municipality	N of children in MMM training across all trial sites
Sep. - Dec. 2017	3	6	3	18	54
Jan. - June 2018	3	6	5	30	90
Aug. - Oct. 2018	3	6	3-4	20-21	62
**Total**					**206**

The schedule leaves room for flexibility across time, meaning that the individual sites may include various numbers of children in each semester depending of the availability of eligible children and trained psychologists. The project leaders will continuously monitor the caseload and the performance at each site and help the municipalities to regulate the flow of children into the study (e.g., by timing and scaling the local initiatives to inform professionals and parents about the study).

It is likely that some of the MMM trainers will leave their job during the trial period due to job changes, maternal leave and so on, in which case the other MMM trainers at the trial site will have to increase their caseload and/or new psychologists will have to be enrolled and trained in order for the trial site to include the number of children specified by its contract.

SUPERVISION OF THE MIND MY MIND TRAINERS

The organization and supervision of PPR psychologists is thoroughly described in manual called "Supervision in the Mind My Mind Project." The manual was written by two of the authors of the MMM training manual.

The supervision focuses on four key points:

1. The case
2. The psychologist's personal style

3. The CBT methodology
4. Fidelity to the MMM program, including how to tailor the training to the individual child (flexibility within fidelity)

Supervision is typically conducted by a more experienced psychologist. In the MMM study, the supervisors are psychologists employed in the regional child and adolescent mental health services (CAP). The recruitment and financial compensation for their work is based on a contract between the head of the CAP and the MMM program. The CAP has a natural interest in providing supervision, as it aims to improve the quality of treatment in PPR by securing therapists' competent adherence to the MMM manual. If the PPR psychologist cannot help the child, the child will be referred for assessment and treatment at a more specialized level (i.e., in the CAP). This way, the supervision model will act as a catalyst for extended collaboration between the PPR and the CAP.

EDUCATION OF TRAINERS AND SUPERVISORS

The supervisors recruited from the CAP are expected to be authorized as clinical psychologists and should be trained in supervision. In addition, the supervisors must have extended theoretical and clinical knowledge of CBT with children and young people, and broad clinical experience working with families affected by mental illness.

Before engagement in the MMM study, the trainers and the supervisors receive 40 hours of education in how to use the MMM manual, including 1 day on general therapeutic competencies, the motivational function of symptoms, how to engage participants and set goals, case formulation and treatment plans; 2 days on modules for anxiety, depression and trauma-related problems; and 2 days on modules for behavioral difficulties. The CBT experts and authors of the MMM manual teach this one-week course for trainers and supervisors. The course will be repeated once every half year in order to include new psychologists as trainers and supervisors if needed during the trial period.

Once the trainers and supervisors have gained some experience with the MMM manual, they will be offered a 2-day booster workshop to discuss the practices and get feedback from the group of trainers, supervisors and authors/teachers of the MMM manual. Furthermore, the project management and researcher leader will be present at the educational courses and workshops to ensure that the program stays in line with the existing evidence and the research aims.

The PPR psychologists are only allowed to use the MMM manual for children allocated to the MMM training group, and only if they participate in the education and the ongoing supervision. To limit the risk of spreading the MMM methods during the study, a numbered manual will be handed out to each trainer and supervisor. This manual is personal and should not be copied or passed on to others by any other method.

ONGOING VIDEO-BASED SUPERVISION

The trainers will receive ongoing supervision in a variety of ways. First and foremost, the trainers must take part in individual weekly one-hour video conferences with the supervisor from the regional CAP. Once every month, the individual supervision is replaced by a two-hour group session for all of the local trainers and their supervisor, who meet face to face at the PPR' office.

According to the contract, the trainers must video record and upload all sessions performed with children and parents to the MMM video database and platform. The supervisor will prepare for the supervision by observing particularly difficult or rewarding sections of the video recordings.

The trainer will mark specific sequences in the video and ask the supervisor for feedback on those sequences for the next supervision. The project management will save the recordings according to an algorithm (see protocol for the sub-study about “Development and evaluation of treatment fidelity measures”).

PROGRAM FIDELITY AND THERAPIST COMPETENCIES

The observation and scoring of video recordings of therapy sessions is the gold standard for measuring the fidelity and implementation of new psychological treatments (84;85). During the RCT study, a representative selection of video recordings of the sessions will be analyzed in order to develop a manual for observer-based ratings of trainer competencies in and fidelity to the MMM manual. The starting point for the development of this rating system will be manuals and scales used for the rating of therapist competencies and program fidelity in comparable programs (86-88).

TREATMENT AS USUAL

The control group will receive the usual support and treatments available in the municipality, depending on the child’s age and specific problems and needs. This so-called “treatment as usual” (TAU) varies considerably between and within the municipalities. TAU may include supportive conversation with a psychologist or social worker, pedagogical advice to the child’s teacher, network meetings with parents, teachers and pedagogues, and individually adapted support in the school setting to increase learning and social functioning of the child. A few municipalities will also offer group-based CBT-like programs for children with anxiety or for parents of children with behavioral problems, but the access to these group interventions are usually restricted due to limited capacity. Furthermore, all of the municipalities offer open and anonymous counselling.

In order to reduce the risk of attrition from the TAU group, we decided to “enhance” the TAU treatment in the following ways:

The child and parents are invited to meet twice with a PPR psychologist (or other professional) who will help the family to seek the relevant treatment in the municipality. The professional cannot be a PPR psychologist who is trained to deliver the MMM intervention. The first meeting must be held no later than 13 days after randomization (i.e., parallel with the first session of MMM training). The aim of the first meeting is to review the problems as identified during the visitation to the MMM study, to suggest relevant treatment offers in the municipality and, finally, to help coordinate the initiatives. The second meeting must be held no later than week 17 (i.e., parallel to the end of the MMM training). The aim of the second meeting is to evaluate the child’s current problems and needs.

The Ph.D. student who is responsible for the associated study on cost will, a priori, map all of the available services in each of the four municipalities and calculate the cost per unit of service. At each follow-up (weeks 18 and 26) the parents will answer web-based questions about all the various kinds of support and treatment that they and their child have received since the previous follow-up. The questionnaire include a comprehensive list of locally available services, asking the parent to say “yes” or “no” to each type of support or treatment, and if “yes,” they must also estimate the intensity/frequency of the intervention received during the period of interest.

DETERIORATION DURING THE TRIAL

In both intervention groups, overt signs of deterioration of the child's condition will trigger the PPR psychologist to re-evaluate the problems in collaboration with the family and to consider referral to a more specialized treatment in the municipality or to the CAP.

ASSESSMENTS OF OUTCOME

The impact of problems, the severity of psychopathology, school attendance problems, daily functioning and quality of life, and parents' perceived stress will be measured by a number of standardized web-based questionnaires completed as a baseline, after the end of the intervention period (week 18) and at the follow-up (week 26). Also, a smaller package of questionnaires is administered every two weeks (see Table 3).

All questionnaires are administered electronically through the MMM database, managed by DEFACTUM, Social and Health Services and Labour Market, Central Denmark Region. The database automatically sends e-mails and SMSs to the participants, linking them to the MMM web-based questionnaires to be filled out during the course of the trial. All participants will log in using a personal username followed by a two-factor login (i.e., a password and a one-time code sent by e-mail and SMS). The informants complete the questionnaires when or where they want to by use of a computer, a tablet or a smart phone. The few participants with no private access to any of these devices will be guided to use public computers (e.g., at the school or the public library). When data are not reported within a predefined time, reminders will be sent out to parents that they and/or their child have questionnaires waiting to be completed. Teachers will also get automatic reminders. If the informants still do not respond after the automatic reminders, the research team will be notified and make contact with the family or the teacher by phone (or by personal mail), asking them to reply and also offering assistance in the case that they have problems accessing the database.

All of the measurements of the outcome are self-, parent- and teacher-reported except for the number of days the child was absent from school during the intervention and follow-up period, which will be drawn from the school registers in the municipality.

Discontinuation of data collection will occur only if parents withdraw informed consent for their child to participate in the trial.

FLOW DIAGRAM

Figure 1

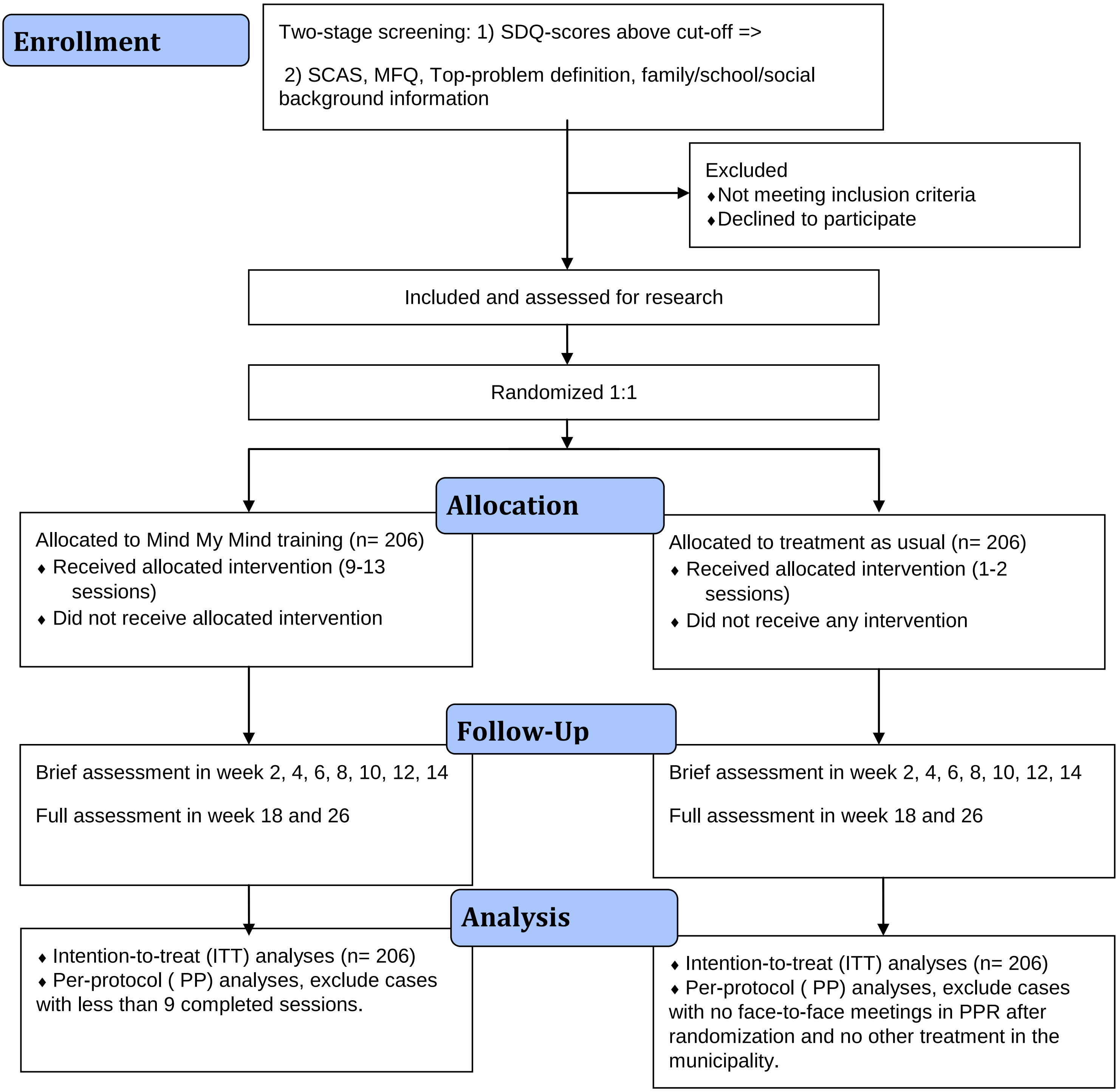

Table 3: Schedule of questionnaires and interviews during visitation, at baseline and during follow-up after randomization to MMM versus TAU

**Time point**	**Psychometric instrument (number of items)**	**Informant**			
**Screening for eligibility**		PPR	Child*	Parent	Teacher
Stage 1					
	Strengths and Difficulties Questionnaire (SDQ+impact) (25+8), asking about the last 6 months		X≥11y	X	
Stage 2	Only if parent's SDQ scores are above the cutoff				
	Spence Children's Anxiety Scale (SCAS) (44)		X≥8y	X	
	Mood and Feelings Questionnaire (MFQ) (34)		X≥8y	X	
	Family/school/social background characteristics			X	
	Top problem as determined by child and parents and recorded by the PPR psychologist (1)	X			
	Informed consent uploaded	X		X	
Inclusion					
DAWBA net					
	Development and Wellbeing Assessment (DAWBA) for ICD-10 and DSM-5 research diagnoses. Includes SDQ+impact		X≥11y	X	
Baseline assessment					
	Strengths and Difficulties Questionnaire (SDQ+impact),		X≥11y	X	X
	Top problem score (1 item, range 1–10)		X	X	
	Spence Children's Anxiety Scale (SCAS) (44)		X≥8y	X	
	Mood and Feelings Questionnaire (MFQ) (34)		X≥8y	X	
	Eyberg Child Behaviour Inventory (ECBI) (36)			X	
	Parental Stress Scale (PSS) (18)			X	
	KIDSCREEN-27 (27)		X	X	
	CHU-9D (9)		X	X	
	Weiss Functional Impairment Rating Scale (WFIRS)(50)			X	
Randomization to MMM vs TAU					
Follow-up at weeks 2, 4, 6, 8, 10, 12 and 14					
	Top problem score (1 item, range 1–10)		X	X	
	Session-by-session (SxS) (7)		X≥11y	X	
At week 2 only	The credibility/expectancy questionnaire (6)			X	
Extended follow-up at week 18 and 26					
	Top problem score (1 item, range 1–10)		X	X	
	Strengths and Difficulties Questionnaire (SDQ+impact), asking about last month		X≥11y	X	X
	Spence Children's Anxiety Scale (SCAS) (44)		x ≥8y	X	

	Mood and Feelings Questionnaire (MFQ) (34)		X ≥8y	X	
	Eyberg Child Behaviour Inventory (ECBI) (36)			X	
	Parental Stress Scale (PSS) (18)			X	
	KIDSCREEN-27 (27)		X	X	
	CHU-9D (9)		X	X	
	Weiss Functional Impairment Rating Scale (WFIRS) (50)			X	
	The Experience of Service Questionnaire (ESQ)		X	X	
	Questions on school absence, parental absence from work, treatment and support received by child and parents via social and health sectors and privately			X	
Scores after each MMM session					
	Records of each session by the trainer: the SMART goals, participants, modules, worksheets, and how the training ended (ending as planned, drop out, referral to support/treatment outside the manual)	X			
	Trainer evaluates the quality of homework done by child and/or parents after each session	X			

The blue questionnaires are administered via the local databases in the municipality after informed consent from the parents. The local PPR psychologists will get access to the information for cases assigned to them. The case records can be printed and stored in the PPR file. The MMM project leader and the research leader will have access to the local data in all sites and hence be able to supervise the local psychologists in the processes of visitation and inclusion of study participants. The local data will be made available for research (see section on databases and data security) through the transfer of pseudo-anonymous data files to the MMM research database. The files are pseudo-anonymous because the data managers will keep a separate key file linking the MMM ID to the name and personal identification number of each child.

The red questionnaires are administered via the MMM research database and by telephone after the parents have given informed consent for their child's participation in the MMM research project. These data are only used for research. The red questionnaires include the repeated measurements of the sense of improvement (SxS, item 1), the impact of problems (SxS, item 2-6) and the hope for the near future (SxS, item 7). See also the presentation of SxS below.

The green questionnaires include the trainers' records of the Mind My Mind training courses and the repeated measurements of the severity of the principal problem (Top problem) as scored by the children and the parents in both intervention groups. The green data are collected via the research database and shared with the municipality. The children and the parents in both intervention groups will get immediate graphical feedback after scoring the Top problem, and the Mind My Mind trainers will use the feedback to monitor the progress over time.

Only children aged 8 and above will be asked to respond to the standardized questionnaires (SCAS, MFQ and CAPS), and in the cases of SDQ, SxS and DAWBA, only children aged 11 and above will be asked to respond. Children of all ages will be asked to score their own Top problem on a scale from 1 to 10, and asked to complete the KIDSCREEN and CHU9D.

TRAINER'S RECORDS OF EACH SESSION

After each scheduled session, the trainer will record whether the session was conducted as planned or cancelled, and if cancelled, by whom and why it was cancelled. The trainer will also record the SMART goals, modules and work sheets that were used in the session (by simple clicking on the number or name of the modules and worksheets). The SMART goals from the first session are automatically repeated in the form, but can be changed if new goals were set during the session. The trainer also reports whether child, parent or both were present, and whether the training primarily targeted the child, parent or both. If the training course ends with this session record, the trainer reports whether the ending was planned, premature or due to drop out because of three missed appointments without cancellation. The trainer will also evaluate the quantity and the quality of the homework done by the participating child and parent (89). The evaluation will help the trainer to plan the next homework assignment while considering how to overcome potential obstacles and prepare home-tasks even better for this particular child and family. Furthermore, the quality of the homework done by the child and/or parents is studied as a putative mediator of treatment effects (90) (see the associated sub study on predictors, moderators and mediators of effect of MMM).

STANDARDIZED PSYCHOMETRIC INTERVIEWS AND QUESTIONNAIRES

The Strengths and Difficulties Questionnaire (SDQ): The SDQ (91-93)is a well-validated, highly structured and commonly used broad, multi-informant assessment and screening instrument for child and adolescent psychopathology. Brevity and many positively worded items characterize the SDQ and make it quick and easy to complete for parent, child and teacher. The psychometric properties of the SDQ have been described extensively (selected studies presented at the SDQ web http://www.sdqinfo.org/py/sdqinfo/f0.py). It consists of 25 questions of positive or negative attributes of the child, each scored on a 3-point Likert scale (0, 1, 2). The 25 items are divided into five sub-scales of five items each, measuring emotional problems, behavioral problems, hyperactivity, peer problems and pro-social behavior. The total difficulties scale (range 0-40) sums up the difficulties across the four problem areas (not including lack of pro-social behavior). The extended version of the SDQ also ask questions about child distress and interference of problems with home-life, friendships, classroom learning and leisure activities, each scored on a 4-point Likert scale (no=0, a little=1, a medium amount=2, a great deal=3). The impact scale sums up the distress and the interference of problems counting only the moderate and severe levels (0=0, 1=0, 2=1, 3=2) into an impact scale (range 0-1). Thus, a one-point reduction on the impact scale corresponds to a change from severe to moderate, or from moderate to little or no impact in one of five important aspects of child's life.

For screening purposes, the SDQ total difficulties, impact and subscale-scores can be used to split a population into high-risk and low-risk groups by defining cut-off for the top 10th percentile of the population. The cut-offs on the parent-reported SDQ used in the MMM study were approximated, based on the distribution of SDQ-scores in the general population-based, birth cohort studies of children aged 5-7 years (94) and aged 11-12 years (95) in Denmark.

The session-by-session (SxS): The SxS is a newly developed measurement for session-by-session monitoring of children's outcomes throughout the course of intervention in mental health services (96). It comprises 1) an initial question of whether difficulties have gotten worse or better since the last visit, 2) the SDQ impact scale; and 3) the hope of improvement. An initial evaluation of the SxS suggested that both clinicians and families found the feedback reports helpful for tracking progress, facilitating communication and engagement, and as a point of reflection (96).

Thus, the impact scale is administered as part of the SDQ or as part of the SxS in both intervention groups at week 0, 2, 4, 6, 8, 10, 12, 14, 18 and 26. The repeated measurements of the impact of problems using the SDQ impact scale enable the study of response trajectories.

The parent reported SDQ impact-scale-score at week 18 is the primary outcome measure. This choice for the primary outcome measure is supported by the psychometric properties of the scale (97). See also the related discussion on page 9.

The Credibility/Expectancy Questionnaire (98) adapted for use in parents who participate in treatment for their child's mental health problems (CEQ—P) (99). The CEQ—P is a brief measure of parents' belief in the treatment credibility (3 items) and their expectancies (feelings) that the treatment will help to improve the child's problems (3 items). The parents' scores on the CEQ—P has been found to correlate with their motivation and to predict adherence to treatment over and above other child and parent characteristics (99). The questionnaire is administered to parents in both intervention groups at week 2. The purposes are: 1) to quantify the group differences in treatment credibility and expectancy, 2) to explore to what degree such differences might explain treatment effects within and between intervention groups, and 3) to describe the correlations between the parent's hope for the near future measured with the SxS and their expectancies for improvement (measured with the CEQ—P). The lack of hope for the near future may be a clinically very important and malleable signal of low expectancies and high risk of drop out.

The Development and well-being assessment (DAWBA): The DAWBA is a package of interviews, questionnaires and rating techniques designed to generate ICD and DSM psychiatric diagnoses in children of age 5–17 years, covering the common emotional, behavioral and hyperactivity disorders (100;101). The child and parent answer highly structured questions independently of each other, and when scoring positive on screening questions the interview opens for additional questions covering all the operationalized diagnostic criteria for a diagnosis within the relevant spectrum of DSM-5 and ICD-10 diagnoses (the Danish version is presently being revised for the DSM-5 instead of DSM-IV diagnoses). Schoolteachers may complete a shorter questionnaire; however, in the present RCT, the schoolteachers are not included as informants for the DAWBA-based research diagnoses - for practical and logistic reasons. The open-ended questions capture the respondent's own description of problems. Experienced child and adolescent psychiatrists will review all the available information, including the computerized integration and presentation of the child and parent's descriptions of the problems, and the algorithm-based estimation of the probability of any specific mental disorder. The final diagnoses are decided by the DAWBA-trained child and adolescent psychiatrists, and when in doubt, a consensus will be reached among two or more raters. Comorbidity will be allowed according to the DSM-5 diagnostic criteria.

The Mood and Feelings Questionnaire (MFQ) (102). The MFQ comprises parent- and youth-reported questionnaires with 34 and 33 items, respectively, each rated on a three-point scale (0-1-2). The English version of MFQ has well-established predictive validity for detection of depressive states, including major depressive episodes and suicidality in epidemiological and clinical samples aged 8–17 years (103). The self-reported MFQ demonstrated a unidimensional structure in a community sample of youths, while the parent-reported MFQ demonstrated a five-factor structure in a clinical sample (103). The five factors were core mood, vegetative, suicidality, cognitive and agitated distress symptom subscales. The agitated distress factor contained symptoms of restlessness, indecision, difficulty thinking, and insomnia, which might reflect general distress or increased levels of arousal. The vegetative symptoms factor captures the down-regulation of biological activity that may be unique to depression (e.g., hypersomnia, psychomotor retardation). There is data supporting that the parent-reported MFQ is sensitive for change with treatment (104). The Danish version will be validated in a

parallel study using a clinical sample from the sample included in the MMM pilot study (study is done in collaboration with postdoctoral researcher Rikke Wesselhoeft).

The Spence Children's Anxiety Scale (SCAS) (105). The SCAS comprises parent- and youth-reported questionnaires with 38 and 44 items, respectively. Each is rated on a four-point scale (0-1-2-3). The self-reported version is longer because it includes six positive filler items. The SCAS consists of six subscales reflecting symptoms specifically related to social phobia (SoP; six items), panic disorder and agoraphobia (Panic; nine items), generalized anxiety disorder (GAD; six items), obsessive-compulsive disorder (OCD; six items), separation anxiety disorder (SAD; six items) and fear of physical injury (Fear; five items). Each subscale is scored separately and added together for a total score reflecting overall anxiety symptoms. A validation of the Danish version of the SCAS in a community sample and a clinical sample of children aged 7–17 years found good internal consistency for the total scale and all subscales (with exception of the subscale for fear of physical injury), satisfactory retest stability, good ability to discriminate between the clinical and community sample, and convergent and divergent validity when compared with relevant questions. Finally, the confirmatory factor analyses supported the original model with six sub-scales. Danish and English versions of the SCAS can be freely obtained from http://www.scaswebsite.com/.

*The Eyberg Child Behavior Inventory (ECBI)(58) .*The ECBI is a brief, parent-reported behavioral rating scale of externalizing problems in children of age 2-16 years. It consists of 36 items that assess typical problem behavior reported by parents of children with oppositional and conduct disorders. Each item is rated on a 7-point Intensity Scale that indicates how often the behaviors currently occur (0=Never, 4=sometimes, 7=always); and a Problem scale that identifies whether the child's behavior is problematic or not for the parent (1=yes, 0=no). Hence the ECBI is scored on two scales, and the sum of scores on the Intensity Scale measures the overall intensity with which the child displays the behaviors, while the sum of scores on the Problem scale measures the number of problematic behaviors. The ECBI is currently used in Denmark in the monitoring of children in the Incredible Years program. The scales are validated for both girls and boys and the normative samples from the US included children with a range of ethnic and socio-demographical backgrounds; however, the Danish norms have not been established.

The KIDSCREEN-27 (106). This is a Health-Related Quality of Life (HRQOL) Questionnaire for Children and Adolescents aged from 8 to 18 years. A total of 13 European countries were included in the cross-cultural harmonization and development of the measures (107). The KIDSCREEN-27 version was developed from the original 52-item version with minimal loss of information. It comprises parent- and youth-reported questionnaires with 27 items, each rated on a five point scale (1-2-3-4-5). It has five dimensions (subscales): Physical Well-Being, Psychological Well-Being, Autonomy & Parents, Peers & Social Support and School Environment. The construct validity of the instrument is satisfying with trends in the expected direction: females tend to show lower HRQOL in the physical and psychological well-being dimensions; older adolescents show lower HRQOL on all dimensions than younger children; children and adolescents with special health care needs display a lower HRQOL in all dimensions in comparison to healthy children and adolescents; and children and adolescents who have spent more than two nights in a hospital in the last year display a lower HRQOL in all dimensions in comparison to healthy children and adolescents.

The Weiss Functional Impairment Rating Scale-Parent Report (WFIRS) (108). The WFIRS was designed to evaluate to what degree a child's behavior or emotional problems impacted various domains of daily functioning. It comprises parent- and youth-reported questionnaires with 50 items, each rated on a four-point scale (0-1-2-3). Although it has only been validated (and found very sensitive for change) in clinical samples of children aged 6–17 years with ADHD, it focuses on the impact of emotional and behavioral problems, and hence shows good face validity as a measurement of daily

functioning in a much broader group of children and youths with emotional and behavioral problems. The WFIRS offers an advantage over use of the Children's Global Assessment Scale (CGAS) by covering a greater range of problems. WFIRS measures six dimensions of functioning (subscales): Family and home life, School and learning, Life skills, Self-Concept, Social activities, and Risk behaviors. Only the parent-reported version is used in the present study.

The Parental Stress Scale (PSS) (109). The PSS comprises a parent-reported questionnaire with 18 items, each rated on a five point scale (0-1-2-3-4). It measures the stress a parent experiences with being a parent of children aged 0–18. The PSS has not yet been validated in Denmark. Data from the pilot sample and from families recruited in the first half of 2017 will be used in a larger validation study in collaboration with the Danish National Centre for Social Research and the Department for Psychology, University of Copenhagen.

The Experience of Service Questionnaire (ESQ) (110). The ESQ comprises parent- and youth-reported questionnaires with 7 items, each rated on a three-point scale (0-1-2). The Danish version was adapted from a longer English questionnaire asking young people and their parents how they felt about the psychiatric service they received. The Danish version proved useful in a recent study of the Cool Kids program (36).

MEASUREMENT OF POTENTIAL HARMS

Information about risks as well as benefits should be made available for service users to make an informed choice of whether or not to engage in treatment. Psychological treatments are often considered to be safe, however, very little is known about the potential harms that may occur during psychotherapy (111-113), particularly for children (113). A review (113) of current practice in the reporting of randomized controlled trials (RCTs) found that only one out of five RCTs of psychological interventions for mental and behavioural disorders reported harm-related information, and usually no information was provided about the applied methods for monitoring harms (113). In spite of poor reporting, evidence converges to suggest that psychological interventions do have the potential to harm certain individuals under certain circumstances. The potential harms include deterioration of target symptoms, development of new symptoms, treatment failure and drop out, and rare and unexpected adverse events (111-113).

There are no standardized methods for measuring the adverse effects of psychological interventions (111), and several methodological challenges have limited research on this issue (111). Firstly, the psychotherapist is the "producer" of treatment and therefore possibly biased towards positive rather than negative results. Secondly, there is no consensus on how to differentiate between positive and negative effects of psychological interventions. Psychological interventions, like the Mind My Mind training, aim to improve mental health by means of strategies that aim to change the cognitions, emotions, and behaviors of the participant in accordance with an explicit psychological theory. According to most theories, meaningful changes may be perceived as unpleasant and may thus cause anxiety and stress. Thus, what is called 'negative' depends on the eye of the beholder. Thirdly, malpractice or poor therapist competence and adherence to the treatment manual may also cause unwanted events, i.e., while good treatment can cause side effects, bad treatment may causes malpractice effects. Finally, there is a lack of differentiation between side effects, deterioration of symptoms, treatment failure, adverse reactions to treatment, and adverse events occurring in parallel to or in the context of the treatment (111).

Recently, Rozental et al. (112) developed a self-reported questionnaire for adults, based on a literature review and a consensus statement on defining and measuring negative effects of internet interventions (112). This 32-item "Negative Effects Questionnaire" (NEQ) demonstrated a six-factor structure: "symptoms" (worries, stress and anxiety), "quality" (difficulties understanding the treatment content, no confidence in treatment or therapist etc.), "dependency" (becoming overly reliant on the treatment or the therapist), "stigma" (fear of being perceived negatively by others), "hopelessness" (lack of hope that things could get better), "failure" (low self-esteem, lost faith in myself). Each of the items are rated "yes"/"no", and if "yes" the informant is further asked about the impact and the causal relationship to treatment. The questionnaire has not been validated in children or adolescents. In the explorative study with adults (112), common negative effects included unpleasant memories, sadness and stress, and the highest impact was linked to increased or novel symptoms, as well as lack of quality in the treatment and therapeutic relationship, suggesting an important link between the treatment quality and fidelity on one hand and the risk of negative effects of treatment on the other hand.

Self-report questionnaires probing for negative effects pose an obvious risk of inducing adverse events by making the participant more aware of the various types of possible adverse events and – at the same time – recognizing these events as negative effects of treatment, rather than effects, which must be expected and which might even signal a healthy development. Furthermore, there is a risk of recall bias and of biases related to the cultural and social beliefs and expectations of the participants, and related to the satisfaction with treatment. If, for instance, the participant is displeased with the treatment or the therapist, this person may exaggerate the adverse events and their impact, and be more likely to explain these negative effects as a result of the treatment.

Studies of trauma-focused therapies have generally been more likely to include harm-related data (113); and one study of trauma-focused CBT for children (114) used a brief checklist including items for suicidality, worsening of target symptom, appearance of new symptoms, etc.

THE PRIMARY MEASURE OF POTENTIAL HARMS

As there is no generally accepted method for the measurement of potential harms of psychological interventions in children and adolescents, we derived two composite scores based on selected items from youth-reported questionnaires already in use in the Mind My Mind Study (Moods and Feelings Questionnaire (MFQ), and the questionnaire used for measuring quality of life (KIDSCREEN)). This way, we can avoid overloading the children with additional questionnaires and also minimize the risk of biases that may result from repeated probing of potential negative effects.

For youths with emotional problems, the most unwanted and potentially dangerous outcome would be suicidality and severe hopelessness and negative self-evaluation, particularly when expressed at the end of the treatment period. Based on the factor structure of the MFQ-items , we selected all items of suicidality ("I thought that life wasn't worth living", "thought about death or dying", "I thought my family would be better off without me", "I thought about killing myself"), and four items of cognitions covering guilt, hopelessness and negative self-evaluation ("I blamed myself for things that weren't my fault", "I thought there was nothing good for me in the future", "I hated myself", "I felt I was a bad person"). Each of the two dimensions, suicidality and negative cognitions, are scored present (”yes”=1, "no"=0) when at least one of the four items are scored "true" (the most severe response) at follow-up (week 18 or week 26) but not at entry. The composite score is derived by addition and dichotomization of scores into 0=”no" and score ≥ 1 = "yes".

THE SECONDARY MEASURES OF POTENTIAL HARMS

Based on our experience from the pilot study, we know that the majority of the children do not enter treatment on their own initiative; rather, it is the parents who seek help on their behalf. Thus, the children may experience strains and limitations in the relationships with the family and their friends, and in their free time. We derived a composite score based on selected items from KIDSCREEN (106) covering a) “family and free time” and b) “friends”. Based on inspection of the content, we selected five items from the first subscale (“Have you had enough time for yourself?”, “Have you been able to do the things that you want to do in your free time?”, “Have your parent(s) had enough time for you?”, “Have your parent(s) treated you fairly?”, “Have you been able talk to your parent(s) when you wanted to?”), and all four items from the second subscale (“Have you spent time with your friends?”, “Have you had fun with your friends?”, “Have you and your friends helped each other?”, “Have you been able to rely on your friends?”). The measures of poor quality in a) family and free time, and b) friends are scored present (”yes”=1, “no”=0) when at least one of the five or four items, respectively, are scored “always” (the most severe response) at follow-up (week 18 or week 26) but not at entry. The composite score of poor quality of life in relation to family, free time and friends is derived by addition and dichotomization of scores into 0=”no” and score ≥ 1 = “yes”.

The correlations between adverse events and benefits of treatment will be explored to inform the field on whether some perceived adverse events may be related to improvements on the primary and key secondary outcomes, for some groups of children. The predictors of adverse events will also be explored, including the credibility/expectancy of treatment.

Furthermore, the data drawn from the national registers covering use of outpatient services, inpatient services, and emergency room visits will be used to measure group differences in service use related to acute crisis, self-harm and suicide attempts. We expect these register-based events to be very rare.

PLAN FOR THE STATISTICAL ANALYSES

All analyses of the primary and secondary outcomes will be intention-to-treat (ITT) with two-sided significance tests.

We will use mixed models with repeated measures for continuous quantities with repeated measures (the SDQ impact-scores and the Top-problem-scores). For binary and non-normally distributed outcomes the generalized linear mixed model will be used, as it provides a more flexible approach for analyzing non-normal data when random effects are present.

In the mixed models with repeated measures, the outcomes will be described as the difference between the mean square estimated means in the two intervention groups at week 18 and at week 26. The mixed models with repeated measures of the SDQ impact-scores and the Top-problem-scores (measured at week 0, 2, 4, 6, 8, 10, 12, 14, 18 and 26) will include as covariates the intervention group indicator (I), time (t) and the interaction between time and the intervention group indicator (t x I) to estimate the effect of treatment. The primary effect of Mind My Mind versus TAU on impact of problems measured with the SDQ-impact-scale at week 18 will be analyzed as the effect of the group indicator (t x I), interpreted according to a significance level of $P<0.05$.

Both linear and generalized linear mixed models allow for ITT-analyses, using maximum-likelihood-based approaches to account for missing data. We will use unstructured covariance matrix, but in cases where this does not converge, the choice of covariance matrix will then rely on comparison of Bayes Information Criteria of models fitted with different covariance matrices.

Continuous outcomes with only one or two post-baseline-assessments will be analyzed using a general linear model (ANCOVA), with multiple imputations with chained equations to handle missing data in accordance with the ITT-principle. A priori, we expect that 100 imputations will be sufficient, but this will be evaluated by the largest fraction of missing information, under the rule-of-thumb that one should have at least 100 times as many imputations as this value.

Dichotomous outcomes will be analyzed using binary logistic regression, using the same multiple-imputation approach as described above.

The models will be adjusted for the stratification variables: 1) region (Zealand or Central Denmark), 2) child age (6–10 years or 11–16 years), and 3) the principal problem (the classification of the Top-problem as 1=anxiety, 2=depressive symptoms or 3=behavioral problems prior to inclusion).

As an exploratory analysis of the primary outcome, the interaction between the intervention group and each of the prognostic variables will be conducted. If $P<0.05$, the corresponding exploratory subgroup analyses will be conducted for the primary outcome. As the principal problem at entry (anxiety, depressive symptoms or behavioral problems) may have a strong influence on the treatment course and outcome, we will explore the effects of Mind My Mind versus TAU on the primary and key secondary outcomes in each of these subgroups.

Furthermore we will explore the outcomes of specific problems of relevance in the corresponding subgroups treated for anxiety, depressive symptoms and/or behavioural problems, including SCAS-scores in the group treated for anxiety, MFQ-scores in the group treated for depressive symptoms, and the ECBI-scores in the group treated for behavioural problems.

THE PRIMARY HYPOTHESIS

We test the *primary research hypothesis* that the intervention-by-time group will show a significant difference in impact of any mental health problems in favor of the Mind My Mind training as compared with TAU at the end of the intervention period (week 18).

This *primary outcome* is measured with the parent-reported SDQ impact-scale (range 0-10) and analyzed in a mixed model with repeated measures (week 0, 2, 4, 6, 8, 10, 12, 14 and 18) adjusting for the stratification variables (see above).

The minimum relevant difference in impact of mental health problems was set at 1.0 on the parent-reported SDQ-impact-scale (range 0-10) corresponding to a change from severe to moderate, or from moderate to little or no impact in one important aspect of child's life (distress, home-life, friendships, classroom learning and leisure activities).

We will use a two-sided test (α= 0.05) to decide if we can reject the null hypothesis that there is no significant difference in impact of problems between the two interventions after 18 weeks.

THE SECONDARY HYPOTHESES AND OUTCOMES

The *first secondary hypothesis* is that the intervention-by-time group will show a significant difference in impact of any mental health problems in favor of the Mind My Mind training as compared with TAU at the end of the follow-up period (week 26).

This *outcome* is also measured with the parent-reported SDQ impact-scale (range 0-10) and analyzed in a mixed model with repeated measures (week 0, 2, 4, 6, 8, 10, 12, 14, 18 and 26) adjusting for the stratification variables (see above). This model tests the sustainability of the primary outcome.

The *key secondary hypotheses* are that the intervention group will show significant differences in endpoint scores of psychopathology (SCAS, MFQ and ECBI sum-scores), daily functioning (WFIRS sum-scores), quality of life (KIDSCREEN, two first sub-scales) and school attendance in favor of the Mind My Mind training as compared with TAU at week 18.

The key secondary outcomes measured at week 18:

a. Anxiety (SCAS, parent-reported)

b. Depressive symptoms (MFQ, parent reported)

c. Level of daily functioning of child (WFIRS, parent reported)

d. School attendance (proportion of school-days where the child is present as recorded by the school)

e. Top-problem scores (parent reported)

f. Quality of life (KIDSCREEN, two subscales on physical well-being and psychological well-being, child reported)

g. Behavioral problems (ECBI, parent reported)

h. Emotional and behavioural problems (SDQ, two subscales, parent reported)

The testing of multiple outcomes requires adjustment of the significance level to control the risk of Type I error. However, many psychological outcomes are partly overlapping or interrelated meaning that the outcome measures are positively correlated. In this case, a simple Bonferroni correction of the overall significance level would be too conservative.

We will use the strategy of hierarchical testing allowing us to preserve the level of significance, $\alpha = 0.05$, as long as the null hypotheses are rejected. All hypotheses will be tested in a fixed sequence starting with the primary hypothesis, the first secondary hypothesis and finally the key secondary hypotheses in the order a, b, c, d, e, f, g, h (see list of key secondary outcomes). However, if a test fails to reach significance ($p<0.05$), we will "fall back" and adjust for multiple testing using the Bonferroni correction in the following tests. Hence the number (n) of hypotheses to be tested after a failure to reject the null hypothesis defines the level of significance ($\alpha = 0.05/n$) for the remaining tests on the list.

Finally, we will explore the same primary and secondary outcomes across all informants (parents, teachers and children) at week 18 and 26, and for the children treated per protocol (see flowchart).

THE HYPOTHESES OF POTENTIAL HARMS

The *primary hypothesis* for group difference in the primary measure of the potentially negative outcome is that the binary logistic regression will show a significantly lower proportion of children

with suicidality and/or negative cognitions in the Mind My Mind group compared with the TAU group at week 18.

The secondary *hypothesis* for group difference in the secondary measure of the negative outcome is that the binary logistic regression will show a significantly lower proportion of children with poor quality of life in the domains of family and free time and/or friends in the Mind My Mind group compared with the TAU group at week 18.

The same hypotheses will be explored at week 26, and with regard to predictors and mediators, including treatment fidelity measures, the parent's and child's experience with treatment, and the parent-reported treatment credibility/expectancy.

Furthermore, group-differences in service use related to acute crisis, self-harm and suicide attempts will be explored, but since these events are rare, these analyses will be considered exploratory, as the trial will not be adequately powered to tests for group differences during the follow-up period of 26 weeks.

MISSING VALUES

There will be no missing baseline values for the primary outcome measure and the stratification variables. The extent to which other variables have missing values will be assessed individually for each of the analyses. Multiple imputations of the missing baseline values and follow-up values will be conducted. However, if only outcome values are missing and there are no good auxiliary variables ($|r|>0.40$), the best approach will be a complete case analysis.

OTHER STATISTICAL ANALYSES

Descriptive statistics will be used to characterize the groups of study participants with regard to sex, age, socio-demographic and family-related background factors, the type and severity of psychopathology, co-occurring problems and functional impairment at inclusion. Descriptive statistics will also be used to compare the group of included and excluded children. Finally, descriptive statistics will be used for the attrition analyses comparing participants per protocol with participants with missing data. Categorical variables will be analyzed using the chi-squared test; and continuous, normally distributed variables are analyzed using the t-test.

SAMPLE SIZE ESTIMATION

The minimal clinical relevant difference in impact of mental health problems between the two randomly allocated intervention groups (MMM versus TAU) measured by the SDQ-impact-scale at week 18 (and at week 26) was set at 1.0 on the parent-reported SDQ-impact-scale (range 0-10) corresponding to a change from severe to moderate, or from moderate to little or no impact in one important aspect of child's life (distress, home-life, friendships, classroom learning and leisure activities).

We decided to use a two-sided α = type-1-error = 0.05, type-2-error = β = 0.10, corresponding to a power of 90%.

Based on the preliminary results (see appendix 1) of analyses of the ongoing feasibility RCT, we estimated a standard deviation of SD=2.5 for the endpoint scores on the SDQ-impact scale in the TAU group. This estimate is very uncertain because it is based on few participants, and in order not to underestimate the SD, we used a slightly broader SD=2.7 in the sample size calculations.

Hence the required sample size necessary to detect or reject a difference of at least 1.0 point was estimated:

N = $2 \times (SD^2 + SD^2) \times (z_{\alpha/2} + z_{\beta}) / MIREDIF^2 = 2 \times 154 = 308$ (z = fractiles in normal distribution).

When allowing for 25% dropout, we need to include and randomize 308/0.75 = 412 children.

Given a power of 90%, a two-sided α of 0.05, and 308 (75%) children assessed at follow-up, we used the standard deviations obtained in the pilot trial to estimate the minimum detectable differences on selected measures of psychopathology, functioning and quality of life, please see appendix 1.

For the testing of secondary outcomes, we selected measures with statistically detectable minimum differences that are also within the range of clinically meaningful effects.

Given a two-sided level of significance set at α = 0.05, a power of 90% and a sample size = 308 (75% of 412) children assessed after end of treatment, the smallest detectable differences between the Mind My Mind and the TAU group in proportions of negative outcomes will be 7.5% versus 16%, or 18% versus 29%; i.e. around 8.5-11.0% difference in proportions, depending on the frequency of the adverse events. If the adverse events occur less frequent and/or the group differences are smaller, the results must be interpreted with caution, as the power is insufficient to rule out type 2 error as well as type 1 error (random error).

The analyses of the negative effects will be intention-to-treat using multiple imputations of the missing follow-up data.

POTENTIAL HARMFUL EFFECTS, RISKS AND INCONVENIENCES

Participants may experience some temporary discomfort as they learn new behaviors. Furthermore, some discomfort may be associated with answering questionnaires on psychopathology.

However, due to the lack of evidence on potential harms related to psychological interventions in children and adolescents, the suicidality, negative cognitions, and poor quality of life in the domains of family, free time and friendships will be monitored.

Furthermore, data on outpatient services, inpatient services, and emergency room visits will be used to measure group-differences in service use related to acute crisis, self-harm and suicide attempts.

USER INVOLVEMENT

The MMM study was initiated with the overarching aim of improving access to high-quality evidence-based treatments in a non-stigmatizing local setting. The children and adolescents recruited for the

pilot study answered questionnaires of user satisfaction after each session and after the end of MMM training, and satisfaction scores were generally very high.

The skewed attrition rate in the on-going pilot study (currently 22% in MMM and 38% in TAU) suggests that although the research methods including the use of self- and parent-reported questionnaires are acceptable for the participants, we need to take steps to improve retention and participation. We therefore carefully enhanced the TAU without compromising the ecologic validity of the study, and we limited the number of research questionnaires and decided to offer a small gift as reward for answering the questionnaires throughout the study.

During the first year of the effectiveness RCT, we will invite study participants to take part in a number of focus group interviews and discussions of questions such as "what worked for you?" and "what was the burden of this intervention?" We will invite children and parents from the MMM group and from the TAU group to separate focus group interviews.

PARTICIPANTS' INTEGRITY AND PRIVACY

NOTIFYING THE DANISH DATA PROTECTION AGENCY OF THE DATABASES

The local databases will be reported to the Data Protection Agency by each of the participating municipalities, who are responsible for the data. The research project and the research database, including the video database, are part of a private health research project.

Private health research projects must be approved by the scientific ethical committee system but should not be reported to the Data Protection Agency, according to a Danish law amendment of May 2012.

Databases information on the study participants are protected under the law on the treatment of personal data in Denmark.

The databases are hosted by the supplier of database, Data & Documentation, DEFACTUM, Social and Health Services and Labour Market, Central Region Denmark. A copy of the research database is delivered to the responsible research manager in the Research Unit, at Child and Adolescent Mental Health Center, the Capital Region of Denmark. The research data are made anonymous for the researchers as the subjects are identified by a unique MMM ID number, and DEFACTUM holds a separate key file with information about the link between ID numbers and the personal civil registration numbers. Thereby, the data analysts are able to test hypotheses blinded for treatment allocation.

DATABASES AND DATA SECURITY

The municipality is the data responsible agency for the local database and for notification of the database to the Data Protection Agency, with PsykiatriFonden as data manager. The database in the municipality contains data from the initial visitation (SDQ, SCAS, MFQ, background information and child and parent's formulation of the child's top problem).

PsykiatriFonden is data responsible for the research database which contains all other data from inclusion, informed consent to participate in the project, randomization, assessments throughout the periods of intervention and follow-up.

The informed consent to participate in the research project includes participants' consent to transferal of the data from the local database in the municipality to the research database for research purposes. There will be no disclosure of other information from the municipality to the research database. There will also be no transfer of medical records, either from the general practitioner, hospital or specialized services.

The research database retrieves all the data collected in accordance with the informed consent to the research project and the study protocol. These data are only available for the principal investigator (PI) and the authorized researchers working on the study protocol.

Furthermore, each of the PPR psychologists who conduct the MMM training will have access to view the following data on their own cases:

1. Data from the visitation
2. Session content and process data entered by the trainer.
3. Child and parent responses to green questionnaires (the "Session-by-Session" and the severity of the top problem).

The participating children, parents and teachers will access the questionnaires electronically by use of personal links sent by e-mail and text message. All participants will log in using a personal user name followed by a two-factor login, i.e., a password and a one-time code send by e-mail and SMS for the final step in the two-factor log in.
If the child is 6-10 years old, the parents' mobile number will be used to send the link by SMS to the child. If the child/adolescent is 11-16 years old and has a mobile number, this is used; otherwise the parents' will be used. The same principle applies to e-mails. If the child is 6-10 years old, the parents' e-mail address will be used to send the link by e-mail to the child. If the child/adolescent is 11-16 years old and has an e-mail address, this is used; otherwise the parents' will be used.

FINANCIAL SUPPORT

The MMM project is initiated by the Danish foundation TrygFonden and headed by Psykiatrifonden in partnership with TrygFonden. TrygFonden provides funding for Psykiatrifonden for payments of the participating municipalities, regions, external consultants and project leaders and managers. The research budget will be administered by the Research Unit, Child and Adolescent Mental Health Center, Capital Region of Denmark. The PI has previously rated applications for TrygFonden for a small fee. The PI has no conflict of interest in relation to the MMM study. The research group will work independently of the sponsor with regard to data collection, statistical analyses, interpretation and dissemination of study results in scientific journals.

FINANCIAL COMPENSATION FOR PARTICIPATION IN RESEARCH ASSESSMENTS

The children will be offered a small financial compensation as incentive for completion of all study measures at pre, post, and follow-up. The aim is to decrease drop out from assessments, particularly in the TAU group. The compensation for completing the whole package of research questionnaires up to week 26 will be a gift card of 250 d.kr. This sum should be allowed by the scientific ethical committee, as the sum is too small to act as the sole motivation for entering the study.

INFORMED CONSENT

The oral and written information about the research project will be presented after the visitation procedures, if child/parent and the PPR psychologist agree on an indicated problem within the domains of anxiety, depressive symptoms and/or behavioral problems, and if the psychologist finds that the child is eligible for inclusion.
The oral and written information is then provided by the same PPR psychologists, who conducted the visitation and who will deliver the MMM training in case the child is included and allocated to the experimental intervention. The PPR psychologists are accustomed to working with children and parents, and will be able to customize the information for the child and the parents according to their educational and cultural background. The child and parents must agree to the place and time of the meeting and that they will be informed about health science research and requested to give informed consent if they want to participate. The written information will be delivered to parents when they are invited to the meeting. If wanted by the young participants of age 15–17 years, they will also get written information about the trial. The research subject and the parent will be made aware that it is possible to bring one accompanying person for the meeting. They will also be informed of the right to take time to consider the information. The meeting will be held on premises in PPR, schools or other municipal sites. The meeting location must be undisturbed and secure privacy and discretion for the potential study participants. The PPR psychologist will schedule one hour for the meeting. During the meeting there will be time for the child and parents to listen to the information and ask questions. The information of the trial will be neutral and delivered without use of technical, therapeutic or value-laden terms, and adjusted to child age, maturity, experience, etc.

Children participating in the trial will receive oral information and be involved in discussion to the extent that the information is age-appropriate and does not harm the child. The child or young person's expressions of their own will—to the extent these are relevant—will be given preference. The written informed consent must be obtained from both custodial parents. Exemption from this rule will not be relevant because the parents are invited to participate in MMM training as support for the child.

Informed consent will include consent to video recordings of all training sessions with child and/or parent. The participants are informed that video recordings will be used for supervision and for quality assurance. Informed consent will also include consent for all the parent- and child-reported answers to questionnaires administered during the visitation, pre- and post-treatment periods to be transferred from the municipal database to the MMM research database for research purposes. The time between the oral and written information and the submission of parents' signature on the consent form will be at least 24 hours, unless the parents directly ask for less time.
The consent follows standard formulations developed by the research ethics committees. The signed consent will be scanned and uploaded in the municipal database and transferred to the research database. The IT system ensures that it is not possible to be included in the trial without written, informed consent.

PARTICIPANTS' ACCESS TO INFORMATION ON THE TRIAL

All children included in the study will be allocated a contact persons in the local PPR (the MMM trainer or the TAU coordinator) and allowed to contact this person at any time if needed for additional information about the trial. All study participants may contact the project manager, Birthe Wielandt Houe (Psykiatrifonden) and the PI (Research Unit, Child and Adolescent Mental Health Center, Capital Region Psychiatry) at all times.

PUBLICATION OF STUDY RESULTS

Information about the study will continuously be disseminated in Danish and international media, on the website of Psykiatrifonden and in scientific journals. All relevant experiences with the development and testing of the intervention, including the development of video-supported supervision and the development of program adherence and trainer competence objectives will be disseminated. Furthermore, we will disseminate our experiences with the introduction of web-based questionnaires as standardized self- and parent-reported outcome measures in a municipal context. Positive, negative and inconclusive results will be made public. The principal investigator will prepare manuscripts for publications in international scientific journals. Coauthorship will be defined in accordance with the Vancouver Guidelines.

ETHICAL CONSIDERATIONS

The feasibility of RCT, including the extension of the trial until the end of April 2017, is approved by the scientific ethical committee in the Capital Region of Denmark (Protocol nr: H-15009648). We will apply to the same scientific ethical committee for approval of the present protocol for the effectiveness of RCT and the associated sub-studies before the end of 2016.

The experimental MMM training comprises evidence methods and techniques that are organized in modules in order to adapt the training to the individual needs of the participants and target the problem formulated by themselves. The control group will receive treatment as usual in the municipality. We therefore expect that children in both groups will experience reduced severity and impact of problems and improved levels of functioning and quality of life during the intervention and follow-up periods. However, study participants (and their parents) may expect some level of temporary discomfort owing to the fact that they are confronting problematic behavior.

Study participants and their parents in both intervention groups will be answering web-based questionnaires and some may find it time-consuming and tiresome to answer questionnaires repeatedly. The child and parents are informed that project participation is voluntary and that they can withdraw their consent at any point in time without providing any reason. Their decisions concerning participation will not in any way affect their access to the usual support and treatment in the municipality.

CLINICAL AND SCIENTIFIC PERSPECTIVES

The study will make it possible to confirm or dismiss the hypothesis that MMM training is superior to treatment that is usual in Danish municipalities.

Whether or not the MMM proves effective when implemented in the real word setting of PPR in Danish municipalities, the trial will generate important new knowledge. All the results will be relevant and inform future implementation and dissemination of indicated prevention and treatment programs for children with emotional and behavioral disturbances. Thus, the study represents an important step towards an organization offering evidence-based treatments at the lowest effective level and with high levels of user involvement and relevance for the participants.

PARTICIPANTS' INSURANCE

PsykiatriFonden has signed insurance (Insurance Number E 9406 716 152, Topdanmark) to cover for injuries that may be connected with participation in the MMM research project. The insurance covers personal injury and/or property damage for a maximum sum of 20,000,000 d.kr. per year.

Reference List

(1) Costello EJ, Copeland W, Angold A. Trends in psychopathology across the adolescent years: what changes when children become adolescents, and when adolescents become adults? J Child Psychol Psychiatry 2011 October;52(10):1015-25.

(2) Polanczyk GV, Salum GA, Sugaya LS, Caye A, Rohde LA. Annual research review: A meta-analysis of the worldwide prevalence of mental disorders in children and adolescents. J Child Psychol Psychiatry 2015 March;56(3):345-65.

(3) Copeland WE, Adair CE, Smetanin P, Stiff D, Briante C, Colman I et al. Diagnostic transitions from childhood to adolescence to early adulthood. J Child Psychol Psychiatry 2013 July;54(7):791-9.

(4) Costello EJ, Mustillo S, Erkanli A, Keeler G, Angold A. Prevalence and development of psychiatric disorders in childhood and adolescence. Arch Gen Psychiatry 2003 August;60(8):837-44.

(5) Kessler RC, Avenevoli S, Costello EJ, Georgiades K, Green JG, Gruber MJ et al. Prevalence, persistence, and sociodemographic correlates of DSM-IV disorders in the National Comorbidity Survey Replication Adolescent Supplement. Arch Gen Psychiatry 2012 April;69(4):372-80.

(6) Copeland WE, Shanahan L, Erkanli A, Costello EJ, Angold A. Indirect comorbidity in childhood and adolescence. Front Psychiatry 2013;4:144.

(7) Meltzer H, Ford T, Goodman R, Vostanis P. The burden of caring for children with emotional or conduct disorders. Int J Family Med 2011;2011:801203.

(8) Snell T, Knapp M, Healey A, Guglani S, Evans-Lacko S, Fernandez JL et al. Economic impact of childhood psychiatric disorder on public sector services in Britain: estimates from national survey data. J Child Psychol Psychiatry 2013 September;54(9):977-85.

(9) Christiansen E, Larsen KJ, Agerbo E, Bilenberg N, Stenager E. Incidence and risk factors for suicide attempts in a general population of young people: a Danish register-based study. Aust N Z J Psychiatry 2013 March;47(3):259-70.

(10) Copeland WE, Angold A, Shanahan L, Costello EJ. Longitudinal patterns of anxiety from childhood to adulthood: the Great Smoky Mountains Study. J Am Acad Child Adolesc Psychiatry 2014 January;53(1):21-33.

(11) Wesselhoeft R, Sorensen MJ, Heiervang ER, Bilenberg N. Subthreshold depression in children and adolescents - a systematic review. J Affect Disord 2013 October;151(1):7-22.

(12) Furlong M, McGilloway S, Bywater T, Hutchings J, Smith SM, Donnelly M. Cochrane review: behavioural and cognitive-behavioural group-based parenting programmes for early-onset

conduct problems in children aged 3 to 12 years (Review). Evid Based Child Health 2013 March 7;8(2):318-692.

(13) Weisz JR, Kuppens S, Eckshtain D, Ugueto AM, Hawley KM, Jensen-Doss A. Performance of evidence-based youth psychotherapies compared with usual clinical care: a multilevel meta-analysis. JAMA Psychiatry 2013 July;70(7):750-61.

(14) Kandel DB, Johnson JG, Bird HR, Weissman MM, Goodman SH, Lahey BB et al. Psychiatric comorbidity among adolescents with substance use disorders: findings from the MECA Study. J Am Acad Child Adolesc Psychiatry 1999 June;38(6):693-9.

(15) Shea BJ, Hamel C, Wells GA, Bouter LM, Kristjansson E, Grimshaw J et al. AMSTAR is a reliable and valid measurement tool to assess the methodological quality of systematic reviews. J Clin Epidemiol 2009 October;62(10):1013-20.

(16) Shea BJ, Grimshaw JM, Wells GA, Boers M, Andersson N, Hamel C et al. Development of AMSTAR: a measurement tool to assess the methodological quality of systematic reviews. BMC Med Res Methodol 2007;7:10.

(17) Davis TE, III, May A, Whiting SE. Evidence-based treatment of anxiety and phobia in children and adolescents: current status and effects on the emotional response. [Review]. Clinical Psychology Review 2011 June;31(4):592-602.

(18) Compton SN, March JS, Brent D, Albano AM, Weersing R, Curry J. Cognitive-behavioral psychotherapy for anxiety and depressive disorders in children and adolescents: an evidence-based medicine review. [Review] [80 refs]. Journal of the American Academy of Child & Adolescent Psychiatry 2004 August;43(8):930-59.

(19) Higa-McMillan CK, Francis SE, Rith-Najarian L, Chorpita BF. Evidence Base Update: 50 Years of Research on Treatment for Child and Adolescent Anxiety. J Clin Child Adolesc Psychol 2016;45(2):91-113.

(20) Reynolds S, Wilson C, Austin J, Hooper L. Effects of psychotherapy for anxiety in children and adolescents: a meta-analytic review. Clinical Psychology Review 2012 June;32(4):251-62.

(21) Mychailyszyn MP, Brodman DM, Read KL, Kendall PC. Cognitive-behavioral school-based interventions for anxious and depressed youth: A meta-analysis of outcomes. [References]. Clinical Psychology: Science and Practice 19[2], 129-153. 2012.

(22) James AC, James G, Cowdrey FA, Soler A, Choke A. Cognitive behavioural therapy for anxiety disorders in children and adolescents. Cochrane Database Syst Rev 2015;(2):CD004690.

(23) Bennett K, Manassis K, Walter SD, Cheung A, Wilansky-Traynor P, Diaz-Granados N et al. Cognitive behavioral therapy age effects in child and adolescent anxiety: an individual patient data metaanalysis. Depress Anxiety 2013 September;30(9):829-41.

(24) Hudson JL, Keers R, Roberts S, Coleman JR, Breen G, Arendt K et al. Clinical Predictors of Response to Cognitive-Behavioral Therapy in Pediatric Anxiety Disorders: The Genes for Treatment (GxT) Study. J Am Acad Child Adolesc Psychiatry 2015 June;54(6):454-63.

(25) Ollendick TH, Jarrett MA, Grills-Taquechel AE, Hovey LD, Wolff JC. Comorbidity as a predictor and moderator of treatment outcome in youth with anxiety, affective, attention

deficit/hyperactivity disorder, and oppositional/conduct disorders. [Review] [160 refs]. Clinical Psychology Review 2008 December;28(8):1447-71.

(26) Lebowitz ER, Shic F, Campbell D, MacLeod J, Silverman WK. Avoidance moderates the association between mothers' and children's fears: findings from a novel motion-tracking behavioral assessment. Depress Anxiety 2015 February;32(2):91-8.

(27) Manassis K, Lee TC, Bennett K, Zhao XY, Mendlowitz S, Duda S et al. Types of parental involvement in CBT with anxious youth: a preliminary meta-analysis. J Consult Clin Psychol 2014 December;82(6):1163-72.

(28) Thulin U, Svirsky L, Serlachius E, Andersson G, Ost LG. The effect of parent involvement in the treatment of anxiety disorders in children: a meta-analysis. Cognitive Behaviour Therapy 2014;43(3):185-200.

(29) Silverman WK, Kurtines WM, Jaccard J, Pina AA. Directionality of change in youth anxiety treatment involving parents: an initial examination. J Consult Clin Psychol 2009 June;77(3):474-85.

(30) Compton SN, Peris TS, Almirall D, Birmaher B, Sherrill J, Kendall PC et al. Predictors and moderators of treatment response in childhood anxiety disorders: results from the CAMS trial. J Consult Clin Psychol 2014 April;82(2):212-24.

(31) Lundkvist-Houndoumadi I, Hougaard E, Thastum M. Pre-treatment child and family characteristics as predictors of outcome in cognitive behavioural therapy for youth anxiety disorders. Nord J Psychiatry 2014 November;68(8):524-35.

(32) Gearing RE, Schwalbe CS, Lee R, Hoagwood KE. The effectiveness of booster sessions in CBT treatment for child and adolescent mood and anxiety disorders. Depression & Anxiety 2013 September;30(9):800-8.

(33) Kendall PC, Hedtke KA. Cognitive-Behavioral Therapy for Anxious Children: Therapist Manual, 3rd Edition. 2006.

(34) Kendall PC, Crawley S, Benjamin C, Mauro C. Brief Coping Cat:Therapist Manual for the 8-session Workbook. 2013.

(35) Rapee RM, Lyneham HJ, Schniering CA, Wutrich V, Abbott MA, Hudson JLA et al. The Cool Kids Child and Adolescent Anxiety Program Therapist Manual. 2006.

(36) Arendt K, Thastum M, Hougaard E. Efficacy of a Danish version of the Cool Kids program: a randomized wait-list controlled trial. Acta Psychiatr Scand 2015 May 27.

(37) David-Ferdon C, Kaslow NJ. Evidence-based psychosocial treatments for child and adolescent depression. [Review] [193 refs]. Journal of Clinical Child & Adolescent Psychology 2008 January;37(1):62-104.

(38) Hetrick SE, Cox GR, Witt KG, Bir JJ, Merry SN. Cognitive behavioural therapy (CBT), third-wave CBT and interpersonal therapy (IPT) based interventions for preventing depression in children and adolescents. Cochrane Database Syst Rev 2016;8:CD003380.

(39) Stice E, Shaw H, Bohon C, Marti CN, Rohde P. A meta-analytic review of depression prevention programs for children and adolescents: factors that predict magnitude of intervention effects. J Consult Clin Psychol 2009 June;77(3):486-503.

(40) Merry SN, Hetrick SE, Cox GR, Brudevold-Iversen T, Bir JJ, McDowell H. Psychological and educational interventions for preventing depression in children and adolescents. Cochrane Database Syst Rev 2011;(12):CD003380.

(41) Weisz JR, McCarty CA, Valeri SM. Effects of psychotherapy for depression in children and adolescents: a meta-analysis. Psychological Bulletin 2006 January;132(1):132-49.

(42) Hetrick SE, Cox GR, Merry SN. Where to go from here? An exploratory meta-analysis of the most promising approaches to depression prevention programs for children and adolescents. Int J Environ Res Public Health 2015 May;12(5):4758-95.

(43) Hetrick SE, Cox GR, Fisher CA, Bhar SS, Rice SM, Davey CG et al. Back to basics: could behavioural therapy be a good treatment option for youth depression? A critical review. Early Interv Psychiatry 2014 April 4.

(44) Cox GR, Callahan P, Churchill R, Hunot V, Merry SN, Parker AG et al. Psychological therapies versus antidepressant medication, alone and in combination for depression in children and adolescents. Cochrane Database Syst Rev 2014;11:CD008324.

(45) Arnberg A, Ost LG. CBT for children with depressive symptoms: a meta-analysis. Cogn Behav Ther 2014;43(4):275-88.

(46) Forti-Buratti MA, Saikia R, Wilkinson EL, Ramchandani PG. Psychological treatments for depression in pre-adolescent children (12 years and younger): systematic review and meta-analysis of randomised controlled trials. Eur Child Adolesc Psychiatry 2016 March 11.

(47) Curry JF, Wells KC, Brent DA, Clarke GN, Rohde P, Albano AM et al. Treatment of Adolescents with Depression Study (TADS) cognitive behavior therapy manual: Introduction, rationale, and adolescent sessions. 2003.

(48) Bearman SK, Ugueto A, Alleyne A, Weisz JR. Adapting CBT for depression to fit diverse youths and contexts: Applying the deployment-focused model of treatment development and testing. 2010.

(49) Bearman SK, Weisz JR. Primary and Secondary Control Enhancement Training (PASCET): Applying the deployment-focused model of treatment development and testing. 2009.

(50) Clarke GN, Hornbrook M, Lynch F, Polen M, Gale J, Beardslee W et al. A randomized trial of a group cognitive intervention for preventing depression in adolescent offspring of depressed parents. Archives of General Psychiatry 2001 December;58(12):1127-34.

(51) Clarke G, Rohde P, Lewinsohn P, Hops H, Seeley J. Cognitive-behavioral treatment of adolescent depression: Efficacy of acute group treatment and booster sessions. Journal of the American Academy of Child & Adolescent Psychiatry 38, 272-279. 1999.

(52) Brunwasser SM, Gillham JE, Kim ES. A Meta-Analytic Review of the Penn Resiliency Program's Effect on Depressive Symptoms. Journal of Consulting & Clinical Psychology 77[6], 1042-1054. 2009.

(53) Stark KD, Streusand W, Krumholz LS, Patel P. Cognitive-behavioral therapy for depression: The ACTION treatment program for girls. 2010.

(54) Battagliese G, Caccetta M, Luppino OI, Baglioni C, Cardi V, Mancini F et al. Cognitive-behavioral therapy for externalizing disorders: A meta-analysis of treatment effectiveness. Behav Res Ther 2015 December;75:60-71.

(55) Mytton J, DiGuiseppi C, Gough D, Taylor R, Logan S. School-based secondary prevention programmes for preventing violence. Cochrane Database Syst Rev 2006;(3):CD004606.

(56) Stoltz S. Stay Cool Kids?! Effectiveness, Moderation and Mediation of a Preventive Intervention for Externalizing Behavior. 2012. Utrecht University Repository (2012).

(57) Wilson SJ, Lipsey MW. School-based interventions for aggressive and disruptive behavior: update of a meta-analysis. Am J Prev Med 2007 August;33(2 Suppl):S130-S143.

(58) Eyberg SM, Nelson MM, Boggs SR. Evidence-based psychosocial treatments for children and adolescents with disruptive behavior. J Clin Child Adolesc Psychol 2008 January;37(1):215-37.

(59) McCart M, Priester P, Davies W, zen R. Differential effectiveness of behavioral parent-training and cognitive-behavioral therapy for antisocial youth: a meta-analysis. Journal of Abnormal Child Psychology 34[4], 525-541. 2006.

(60) Michelson D, Davenport C, Dretzke J, Barlow J, Day C. Do evidence-based interventions work when tested in the "real world?" A systematic review and meta-analysis of parent management training for the treatment of child disruptive behavior. Clin Child Fam Psychol Rev 2013 March;16(1):18-34.

(61) Webster-Stratton C, Rinaldi J, Jamila MR. Long-Term Outcomes of Incredible Years Parenting Program: Predictors of Adolescent Adjustment. Child Adolesc Ment Health 2011 February;16(1):38-46.

(62) Forgatch MS, Patterson GR, Degarmo DS. Evaluating fidelity: predictive validity for a measure of competent adherence to the Oregon model of parent management training. Behav Ther 2005;36(1):3-13.

(63) Forgatch MS, Patterson GR, Degarmo DS, Beldavs ZG. Testing the Oregon delinquency model with 9-year follow-up of the Oregon Divorce Study. Dev Psychopathol 2009;21(2):637-60.

(64) Wilson P, Rush R, Hussey S, Puckering C, Sim F, Allely CS et al. How evidence-based is an 'evidence-based parenting program'? A PRISMA systematic review and meta-analysis of Triple P. BMC Med 2012;10:130.

(65) Kazdin A, Esveldt-Dawson K, French N, Unis A. Problem-Solving Skilss Training and Realtionship Therapy in the Treatment of Antisocial Child Behavior. Journal of Consulting and Clinical Psychology 55[1], 76-85. 1987.

(66) Kazdin A, Siegel T, Bass D. Cognitive Problem-Solving Skilss Training and Parent Management training in the Treatment of Antisocial Behavior in Children. Journal of Consulting and Clinical Psychology 60[5], 733-747. 1992.

(67) Lochman JE, Wells KC. The coping power program for preadolescent aggressive boys and their parents: outcome effects at the 1-year follow-up. J Consult Clin Psychol 2004 August;72(4):571-8.

(68) Lochman JE, Boxmeyer C, Powell N, Qu L, Wells K, Windle M. Dissemination of the Coping Power program: importance of intensity of counselor training. J Consult Clin Psychol 2009 June;77(3):397-409.

(69) Lochman JE, Powell N, Boxmeyer C, Andrade B, Stromeyer SL, Jimenez-Camargo LA. Adaptations to the coping power program's structure, delivery settings, and clinician training. Psychotherapy (Chic) 2012 June;49(2):135-42.

(70) Lochman JE, Wells KC, Qu L, Chen L. Three year follow-up of coping power intervention effects: evidence of neighborhood moderation? Prev Sci 2013 August;14(4):364-76.

(71) Lochman JE, Baden RE, Boxmeyer CL, Powell NP, Qu L, Salekin KL et al. Does a booster intervention augment the preventive effects of an abbreviated version of the coping power program for aggressive children? J Abnorm Child Psychol 2014;42(3):367-81.

(72) Stoltz S, van Londen M, Dekovic M, Orobio de Castro B, Prinzie P, Lochman JE. Effectiveness of an individual school-based intervention for children with aggressive behaviour: a randomized controlled trial. Behavioural and Cognitive psychotherapy 41[5], 525-548. 2013.

(73) Stoltz S, Dekovic M, van Londen M, Orobio de Castro B, Prinzie P. What Works for Whom, How and under What Cicumstances? Testing Moderated Mediation of Intervention Effects on Externalizing Behavior in Children. Social Development 22[2], 406-425. 2013.

(74) Chorpita B, Weisz JR. MATCH-ADTC Modular Approach to Therapy for Children with Anxiety, Depression, Trauma, or Conduct Problems. 2009.

(75) Chorpita BF, Daleiden EL, Weisz JR. Identifying and selecting the common elements of evidence based interventions: a distillation and matching model. Ment Health Serv Res 2005 March;7(1):5-20.

(76) Weisz JR, Chorpita BF, Palinkas LA, Schoenwald SK, Miranda J, Bearman SK et al. Testing standard and modular designs for psychotherapy treating depression, anxiety, and conduct problems in youth: a randomized effectiveness trial. Arch Gen Psychiatry 2012 March;69(3):274-82.

(77) Chorpita BF, Weisz JR, Daleiden EL, Schoenwald SK, Palinkas LA, Miranda J et al. Long-term outcomes for the Child STEPs randomized effectiveness trial: a comparison of modular and standard treatment designs with usual care. J Consult Clin Psychol 2013 December;81(6):999-1009.

(78) Park AL, Tsai KH, Guan K, Reding ME, Chorpita BF, Weisz JR. Service Use Findings from the Child STEPs Effectiveness Trial: Additional Support for Modular Designs. Adm Policy Ment Health 2015 January 13.

(79) Bullis JR, Sauer-Zavala S, Bentley KH, Thompson-Hollands J, Carl JR, Barlow DH. The unified protocol for transdiagnostic treatment of emotional disorders: preliminary exploration of effectiveness for group delivery. Behav Modif 2015 March;39(2):295-321.

(80) Farchione TJ, Fairholme CP, Ellard KK, Boisseau CL, Thompson-Hollands J, Carl JR et al. Unified protocol for transdiagnostic treatment of emotional disorders: a randomized controlled trial. Behav Ther 2012 September;43(3):666-78.

(81) Girio-Herrera E, Ehrenreich-May J. Using flexible clinical processes in the Unified Protocol for the Treatment of Emotional Disorders in Adolescence. Psychotherapy (Chic) 2014 March;51(1):117-22.

(82) Bilek EL, Ehrenreich-May J. An open trial investigation of a transdiagnostic group treatment for children with anxiety and depressive symptoms. Behav Ther 2012 December;43(4):887-97.

(83) Ehrenreich-May J, Bilek E, Queen A, Hernandez Rodriguez J. A Unified Protocol for the Group Treatment of Childhood Anxiety and Depression. Revista de Psicopatología y Psicología Clínica 17[3], 219-236. 2012.

(84) Cross W, West J, Wyman PA, Schmeelk-Cone K, Xia Y, Tu X et al. Observational measures of implementer fidelity for a school-based preventive intervention: development, reliability, and validity. Prev Sci 2015 January;16(1):122-32.

(85) Schoenwald SK, Garland AF. A review of treatment adherence measurement methods. Psychol Assess 2013 March;25(1):146-56.

(86) Forgatch MS, Degarmo DS. Sustaining fidelity following the nationwide PMTO implementation in Norway. Prev Sci 2011 September;12(3):235-46.

(87) Schoenwald SK, Garland AF, Chapman JE, Frazier SL, Sheidow AJ, Southam-Gerow MA. Toward the effective and efficient measurement of implementation fidelity. Adm Policy Ment Health 2011 January;38(1):32-43.

(88) Schoenwald SK, Garland AF, Southam-Gerow MA, Chorpita BF, Chapman JE. Adherence Measurement in Treatments for Disruptive Behavior Disorders: Pursuing Clear Vision through Varied Lenses. Clin Psychol (New York) 2011 December 1;18(4):331-41.

(89) Arendt K, Thastum M, Hougaard E. Homework Adherence and Cognitive Behaviour Treatment Outcome for Children and Adolescents with Anxiety Disorders. Behav Cogn Psychother 2016 March;44(2):225-35.

(90) Westra HA, Dozois DJ, Marcus M. Expectancy, homework compliance, and initial change in cognitive-behavioral therapy for anxiety. J Consult Clin Psychol 2007 June;75(3):363-73.

(91) Goodman A, Goodman R. Strengths and difficulties questionnaire as a dimensional measure of child mental health. J Am Acad Child Adolesc Psychiatry 2009 April;48(4):400-3.

(92) Goodman R, Meltzer H, Bailey V. The Strengths and Difficulties Questionnaire: a pilot study on the validity of the self-report version. Eur Child Adolesc Psychiatry 1998 September;7(3):125-30.

(93) Goodman R. The extended version of the Strengths and Difficulties Questionnaire as a guide to child psychiatric caseness and consequent burden. J Child Psychol Psychiatry 1999 July;40(5):791-9.

(94) Niclasen J, Teasdale TW, Andersen AM, Skovgaard AM, Elberling H, Obel C. Psychometric properties of the Danish Strength and Difficulties Questionnaire: the SDQ assessed for more than 70,000 raters in four different cohorts. PLoS One 2012;7(2):e32025.

(95) Jeppesen P, Clemmensen L, Munkholm A, Rimvall MK, Rask CU, Jorgensen T et al. Psychotic experiences co-occur with sleep problems, negative affect and mental disorders in preadolescence. J Child Psychol Psychiatry 2015 May;56(5):558-65.

(96) Hall CL, Taylor J, Moldavsky M, Marriott M, Pass S, Newell K et al. A qualitative process evaluation of electronic session-by-session outcome measurement in child and adolescent mental health services. BMC Psychiatry 2014;14:113.

(97) Stringaris A, Goodman R. The value of measuring impact alongside symptoms in children and adolescents: a longitudinal assessment in a community sample. J Abnorm Child Psychol 2013 October;41(7):1109-20.

(98) Devilly GJ, Borkovec TD. Psychometric properties of the credibility/expectancy questionnaire. J Behav Ther Exp Psychiatry 2000 June;31(2):73-86.

(99) Nock MK, Ferriter C, Holmberg E. Parent Beliefs about Treatment Credibility and Effectiveness: Assessment and Relation to Subsequent Treatment Participation. J Child Fam Stud 2007;16:27-38.

(100) Goodman A, Heiervang E, Collishaw S, Goodman R. The 'DAWBA bands' as an ordered-categorical measure of child mental health: description and validation in British and Norwegian samples. Soc Psychiatry Psychiatr Epidemiol 2011 June;46(6):521-32.

(101) Goodman R, Ford T, Richards H, Gatward R, Meltzer H. The Development and Well-Being Assessment: description and initial validation of an integrated assessment of child and adolescent psychopathology. J Child Psychol Psychiatry 2000 July;41(5):645-55.

(102) Daviss WB, Birmaher B, Melhem NA, Axelson DA, Michaels SM, Brent DA. Criterion validity of the Mood and Feelings Questionnaire for depressive episodes in clinic and non-clinic subjects. J Child Psychol Psychiatry 2006 September;47(9):927-34.

(103) Jeffreys M, Rozenman M, Gonzalez A, Warnick EM, Dauser C, Scahill L et al. Factor Structure of the Parent-Report Mood and Feelings Questionnaire (MFQ) in an Outpatient Mental Health Sample. J Abnorm Child Psychol 2016 August;44(6):1111-20.

(104) Vostanis P, Feehan C, Grattan E, Bickerton WL. A randomised controlled out-patient trial of cognitive-behavioural treatment for children and adolescents with depression: 9-month follow-up. J Affect Disord 1996 September 9;40(1-2):105-16.

(105) Arendt K, Hougaard E, Thastum M. Psychometric properties of the child and parent versions of Spence children's anxiety scale in a Danish community and clinical sample. J Anxiety Disord 2014 December;28(8):947-56.

(106) Ravens-Sieberer U, Auquier P, Erhart M, Gosch A, Rajmil L, Bruil J et al. The KIDSCREEN-27 quality of life measure for children and adolescents: psychometric results from a cross-cultural survey in 13 European countries. Qual Life Res 2007 October;16(8):1347-56.

(107) Ravens-Sieberer U, Herdman M, Devine J, Otto C, Bullinger M, Rose M et al. The European KIDSCREEN approach to measure quality of life and well-being in children: development, current application, and future advances. Qual Life Res 2014 April;23(3):791-803.

(108) Tarakcioglu MC, Cakin MN, Olgun NN, Aydemir O, Weiss MD. Turkish validity and reliability study of the Weiss Functional Impairment Rating Scale-Parent Report. Atten Defic Hyperact Disord 2014 November 27.

(109) Oronoz B, Alonso-Arbiol I, Balluerka N. A Spanish adaptation of the Parental Stress Scale. Psicothema 2007 November;19(4):687-92.

(110) Attride-Stirling J, Humphrey C, Tennison B, Cornwell J. Gathering data for health care regulation: learning from experience in England and Wales. J Health Serv Res Policy 2006 October;11(4):202-10.

(111) Linden M, Schermuly-Haupt ML. Definition, assessment and rate of psychotherapy side effects. World Psychiatry 2014 October;13(3):306-9.

(112) Rozental A, Kottorp A, Boettcher J, Andersson G, Carlbring P. Negative Effects of Psychological Treatments: An Exploratory Factor Analysis of the Negative Effects Questionnaire for Monitoring and Reporting Adverse and Unwanted Events. PLoS One 2016;11(6):e0157503.

(113) Jonsson U, Alaie I, Parling T, Arnberg FK. Reporting of harms in randomized controlled trials of psychological interventions for mental and behavioral disorders: a review of current practice. Contemp Clin Trials 2014 May;38(1):1-8.

(114) Scheeringa MS, Weems CF, Cohen JA, Amaya-Jackson L, Guthrie D. Trauma-focused cognitive-behavioral therapy for posttraumatic stress disorder in three-through six year-old children: a randomized clinical trial. J Child Psychol Psychiatry 2011 August;52(8):853-60.

Section 1: Administrative information

Working Title

Transdiagnostic, Cognitive and Behavioral Intervention versus Treatment As Usual for School-Aged Children with Emotional and Behavioral Disturbances: Statistical Analysis Plan for The Mind My Mind Study - A Pragmatic Randomized Effectiveness Trial

Collaborators/Authors

Pia Jeppesen[1,2], Rasmus Trap Wolf[1,3], Sabrina M. Nielsen[4,5], Robin Christensen[4,5], Kerstin von Plessen[1,6], Niels Bilenberg[7,8], Per Hove Thomsen[9,10], Mikael Thastum[11], Simon-Peter Neumer[12,13], Louise Berg Puggaard[1], Mette Maria Agner Pedersen[1], Anne Katrine Pagsberg[1,2], Wendy Silverman[14], Christoph U. Correll[15,16,17,18].

[1]Child and Adolescent Mental Health Centre, Mental Health Services – Capital Region of Denmark, Copenhagen, Denmark

[2]Department of Clinical Medicine, Faculty of Health and Medical Sciences, University of Copenhagen, Denmark

[3]Danish Centre for Health Economics, Department of Public Health, University of Southern Denmark, Denmark

[4]Musculoskeletal Statistics Unit, The Parker Institute, Bispebjerg and Frederiksberg Hospital, Copenhagen, Denmark

[5]Research Unit of Rheumatology, Department of Clinical Research, University of Southern Denmark, Odense University Hospital, Denmark

[6]Division of Child and Adolescent Psychiatry, Department of Psychiatry, Lausanne University Hospital CHUV, Switzerland.

[7]Department for Child and Adolescent Psychiatry, Mental Health Services in the Region of Southern Denmark.

[8]University of Southern Denmark, Odense, Denmark.

[9]Research Center at Department for Child- and Adolescent Psychiatry, Psychiatry, Aarhus University Hospital, Skejby, Denmark.

[10]Institute of Clinical Medicine, Aarhus University, Aarhus, Denmark.

[11]Centre for the Psychological Treatment of Children and Adolescents, Department of Psychology and Behavioural Sciences, Aarhus BSS, Aarhus University, Denmark.
[12]Centre for Child and Adolescent Mental Health, Oslo, Norway.
[13]The Arctic University of Norway, Centre for Child and Youth Mental Health and Child Welfare, North Norway (RKBU North), Tromsø, Norway.
[14]Anxiety and Mood Disorders Program, Yale Child Study Center, Yale School of Medicine, New Haven, CT, USA.
[15]Donald and Barabara Zucker School of Medicine at Hofstra/Northwell, Department of Psychiatry and Molecular Medicine, Hempstead, New York, USA
[16]The Zucker Hillside Hospital, Department of Psychiatry, Glen Oaks, New York, USA.
[17]Feinstein Institute for Medical Research, Center for Psychiatric Neuroscience, Manhasset, New York, USA
[18]Charité Universitätsmedizin, Department of Child and Adolescent Psychiatry, Berlin, Germany

Trial Registration

Clinical Trials Identifier: NCT03535805
Danish Ethics Committee: Journal-nr.: 17011408, Date of approval: June 20th, 2017

SAP Version

Version 1.0 Date: August 5th, 2019

Protocol Version

Version 1.2 Date: March 27th, 2019

Roles and Responsibility – non-signatory names and contribution

Project Leader:	Pia Jeppesen, PhD, Associate Clinical Professor
Advisory Board:	Anne Katrine Pagsberg, Professor
	Kerstin Plessen, Professor
	Niels Bilenberg, Professor
	Per Hove Thomsen, Professor
	Mikael Thastum, Professor
	Simon-Peter Neumer, Ph.D., Senior Researcher
	Wendy Silverman, Professor
	Christoph U. Correll, Professor
Senior Biostatistician:	Robin Christensen, MSc, PhD (Biostatistics), Professor
Statistical Analysis:	Sabrina M. Nielsen, MSc, PhD Fellow (Biostatistics)

Roles and Responsibility – signatures

Person performing the analyses: Sabrina M. Nielsen

Signature [signature] 7/8 - 19 Date

Senior biostatistician responsible: Robin Christensen

Signature [signature] Date Aug. 8, 2019

Chief investigator/clinical lead: Pia Jeppesen

Signature Pia Jeppesen Date August 7th 2019

Section 2: Introduction

Background and Rationale

Impairing emotional and behavioral problems are common in children and adolescents and mark a three-fold increased risk of mental disorder in young adulthood.[1-3] Evidence-based psychological interventions are recommended for indicated prevention and first-line treatment[4], but access to treatment is often limited.

A new, transdiagnostic and modular, cognitive and behavioral therapy program '*Mind My Mind*' (MMM) comprising evidence-based interventions for children with emotional and behavioral problems was designed to be delivered by educational psychologists in the Danish municipalities. A feasibility randomized controlled trial (RCT) (NCT03448809), demonstrated that the study design was acceptable among children, parents, and therapists, and it provided data to estimate the sample size needed for a pivotal RCT.

Aim and Objectives

Our aim is to investigate the effectiveness and cost-effectiveness of MMM compared with '*Treatment As Usual*' (TAU) for children and adolescents with impairing anxiety, depressive symptoms and/or behavioral problems. Both beneficial and harmful effects will be evaluated.

The primary objective is to compare the effectiveness of MMM, relative to TAU, on the impact of mental health problems reported by the parent using the impact scale of the Strengths and Difficulties Questionnaire (SDQ) at the end of treatment (week 18). The SDQ-impact scale evaluates the impact of mental health problems on the everyday life of the child, i.e., how much the difficulties upset the child and interfere with the child's home life, friendships, classroom learning, and leisure activities.

The hypothesis was that the parent-reported impact of problems will be significantly lower for children in the MMM group compared with children in the TAU group after the 18-week intervention period (primary hypothesis).

Key secondary objectives are to compare the effectiveness of MMM, relative to TAU, measured at week 18 on level of (*i*) Anxiety (Spence Children's Anxiety Scale [SCAS], parent-reported), (*ii*) Depressive symptoms (Mood and Feelings Questionnaire [MFQ], parent reported), (*iii*) Daily functioning of child (Weiss Functional Impairment Rating Scale [WFIRS], parent reported), (*iv*) School attendance (proportion of school-days within the last 4 weeks, where the child is present as recorded by the parent), (*v*) Top-problem scores (parent reported), Quality of life (KIDSCREEN-27) two subscales on (*vi*) physical well-being and (*vii*) psychological well-being (child reported), Behavioral problems (Eyberg Child Behavior Inventory [ECBI]), (*viii*) ECBI intensity score (parent reported), (ix) ECBI problem

score (parent reported), and (*x*) Emotional and behavioral problems (SDQ total difficulties scale, parent reported).

The hypotheses were that the severity of key secondary outcomes of psychopathology, daily functioning, school attendance and health-related quality of life would show significant group differences in favor of MMM compared with TAU at week 18 (secondary hypotheses).

Exploratory objectives are:
To compare the proportion of children with an SDQ impact score reduction ≥ 1 point from baseline to week 18, and the proportion of children scoring below the SDQ inclusion cut-off at week 18 (see inclusion criteria 2, page 11)

To compare the sustained effectiveness of MMM, relative to TAU from baseline to week 26 (i.e., 8 weeks after cessation of the MMM intervention) on the impact of mental health problems reported by the parent (using SDQ), as well as the level of (*i*) Anxiety (SCAS, parent-reported), (*ii*) Depressive symptoms (MFQ, parent reported), (*iii*) Daily functioning of child (WFIRS, parent reported), (*iv*) School attendance (proportion of school-days within the last four weeks where the child is present as recorded by the parent), (*v*) Top-problem scores (parent reported), Quality of life (KIDSCREEN-27) two subscales on (*vi*) physical well-being and (*vii*) psychological well-being, child reported), Behavioral problems (ECBI), (*viii*) ECBI intensity score (parent reported) (ix) ECBI problem score (parent reported), and (*x*) Emotional and behavioral problems (SDQ total difficulties scale, parent-reported). Furthermore, to compare proportion of children with SDQ impact score reduction ≥ 1 point from baseline to week 26, and proportion of children scoring below the SDQ inclusion cut-off at week 26.

To compare the effectiveness of MMM, relative to TAU, on the different measures of psychopathology, functioning, and quality of life that are not already part of the primary, secondary or exploratory objectives. These exploratory outcomes include responses from children at week 18 (i.e., at the end of MMM intervention), and at week 26 (i.e., 8 weeks after cessation of the MMM intervention). Some of the questionnaires could not be administered to children below the age of 8 years (SCAS and MFQ) or below the age of 11 years (SDQ), as they are not standardized for children this age. Hence, we compare the impact of mental health problems (SDQ-impact score) and the emotional and behavioral difficulties (SDQ-total difficulties score) as reported by children of age 11-16 years, and anxiety (SCAS) and depressive symptoms (MFQ) as reported by children of age 8-16 years in the randomized design. The Top-problem score and the Health-Related Quality of Life (KIDSCREEN-27) had no age-restrictions and was administered to all children.

Furthermore, we will explore the level of satisfaction measured by the parent- and child-reported Experience of Service Questionnaire (ESQ), the teacher-reported impact of mental health problems (SDQ-impact scale, range 0-6), the teacher-reported emotional and behavioral difficulties (SDQ-total difficulties scale), the parent-reported KIDSCREEN-27

scale scores (Physical well-being, Psychological well-being, Autonomy & Parents, Peers & Social Support and School Environment); and finally, the parent's response on the Parental Stress Scale (PSS) in MMM, compared to TAU at week 18, and at week 26.

Measures of potential harms

There are no well validated instruments available to monitor safety and potential hams of psychological interventions in children and adolescents. Instead of introducing specific questionnaires, we defined two binary composite scores. One for suicidality and negative cognitions (the primary measure of harms) based on selected items from MFQ for children aged 8-16 years, and another one for poor quality of family relationships, free time and friendships (the secondary measure of harms) based on selected items from KIDSCREEN-27 for all the children. The self-reported MFQ is prioritized because suicidality and negative cognitions are internalized symptoms that parents are often not or only insufficiently aware of.

If at least one out of the selected items (four items of suicidality, and four items of guilt, hopelessness and negative self-evaluation) is scored "always" (the most severe response) at follow-up, but not at entry, then a potential harm is counted as present (yes/no) in the domain of suicidality and/or negative cognitions. If at least one out of the selected items (five items of family relations and free time, and four items of friendship), is scored "always" (the most severe response) at follow-up, but not at entry, then a potential harm is counted as present (yes/no) in the domain of family relations/free time and/or friends.

We compare the effectiveness of MMM, relative to TAU, on the proportion of children with suicidality and/or negative cognitions at week 18, and at week 26. We compare the effectiveness of MMM, relative to TAU, on the proportion of children with poor quality of family relationships, free time and friendships at week 18, and at week 26.

The economic evaluation will comprise both a cost-effectiveness analysis (CEA) and a cost-utility analysis (CUA) comparing the effects of MMM versus TAU with the cost of MMM versus TAU, including health services, social services and parental cost from baseline to the end of the post-intervention follow-up period (week 26). The detailed listing of the cost to the different stakeholders (municipality, region, private households) will not be available for the present report and will be delivered at a later time point.

Section 3: Study Methods

Trial Design and Interventions

A total of 396 children were included and randomized. The trial was designed as a pragmatic, multi-site, randomized, parallel-group, controlled trial for children aged 6-16 years with emotional and behavioral difficulties. Treatment allocation was performed in a 1:1 ratio. Patients were randomized to either Mind My Mind (MMM) or Treatment as Usual (TAU) -control.

MMM: is a newly developed, transdiagnostic and modular cognitive and behavioral therapy (CBT) program comprising evidence-based interventions for children and adolescents with anxiety, depression or disruptive behavior. The evidence-based CBT methods were organized into modules, and the intervention was tailored to the individual child by the dosing and the sequencing of the modules. Parents were engaged in child's therapy and supported the child in doing the homework. Parent management training was offered for behavioral disturbances. The educational psychologists delivered the therapy after a one-week training in the manualized intervention, followed by weekly supervision. The treatment fidelity was monitored by video observation of therapy sessions. The full MMM training program consisted of 9-13 sessions plus one booster session, which each were completed within 17 weeks.

TAU: The parents in the TAU group were offered two sessions (at week 2 and week 17) to support them to seek help for the child in the municipality. This care coordination visit was provided by psychologists (or other local professionals) who hold records of the currently available treatment options in the municipality. TAU varies considerably from no intervention to counselling, talk therapy, pedagogical advice, network meetings, and/or individual support in the school setting. Some children are offered CBT interventions, but access to manualized treatment is almost inexistent.

Randomization and Allocation Concealment

Allocation concealment was done through centralized randomization, which was provided by the Data & Documentation department named DEFACTUM, Social and Health Services and Labour Market, Central Denmark Region. A computer-generated allocation sequence with variable and unknown block size was employed. Randomization was performed centrally, with stratification, and the sequence was generated by an independent statistician using a random number generator. The principal investigator, the project manager and all researchers and therapists were blinded to the sequence used. To optimize comparability between the two treatment groups, randomization was stratified across three factors:

1) Municipality (2 levels: Vordingborg & Næstved OR Holstebro & Helsingør)
2) Child's age (2 levels: 6–10 years OR 11–16 years)
3) The principal problem classified by the psychologist during the visitation (3 levels: the classification of the top-problem as 1=anxiety OR 2=depressive symptoms OR 3=behavioral problems; the classification of the principal problem into one of the three categories was mandatory, otherwise the child was not eligible).

For each stratum (with a total of 12 strata), computer-generated random allocation sequence lists were prepared by DEFACTUM. This central treatment allocation system was set up to ensure allocation concealment, so that the person enrolling participants did not know in advance which treatment the next child would get. Randomization was done after eligibility criteria and baseline assessments were electronically confirmed in the web-based case report form (see section 5). Following this approach, we masked knowledge of forthcoming assignments.

Power and Sample Size Considerations

The minimal relevant difference (MIREDIF) in impact of mental health problems between the two randomly allocated intervention groups (MMM versus TAU) measured by the SDQ-impact-scale at week 18 (and subsequently at week 26) was set at 1.0 units on the parent-reported SDQ-impact-scale (range 0-10), potentially corresponding to a change from severe to moderate, or from moderate to little or no impact in one important aspect of the child's life (distress, home-life, friendships, classroom learning or leisure activities).

We pre-specified the use of a two-sided type-1 error of 0.05 ($P<0.05$), with a risk of type-2 error of 0.10 (corresponding to a statistical power of 90%). To estimate the sample size needed, we used an expected SD of 2.7 (based on parameters from the feasibility trial) for the SDQ-impact-scale scores. Hence the required sample size necessary to detect or reject a difference of at least 1.0 point was estimated as 2 × 154 children =308 in total:

R software (version 3.4.3)code:

```
power.t.test(delta=1.0, sd=2.7, sig.level = 0.05, power=0.90)
```

Allowing for an attrition rate for up to 25%, it was decided to include and randomize 308/0.75 = 412 children in total. We randomized a total number of 396 children within the recruitment period and decided not to extend the recruitment period since attrition rates were lower than expected (approximately 15%, whereas power calculations allowed 25% attrition), meaning that the necessary number of children with follow-up data had been included.

Framework

The Mind-My-Mind trial protocol states that a secondary objective is to determine whether a potentially superior effect of MMM (over TAU) can be sustained even with a further 8-week extension added (assessed after 26 weeks). Therefore, the exploratory outcomes are tested for superiority to confirm a potential claim of sustained effectiveness related to MMM therapy for the primary outcome (SDQ-impact).

Statistical Interim Analyses and Stopping Guidance

Interim data analysis for effectiveness was not conducted due to the relatively small sample size and short duration of this clinical trial. Safety data were not assessed systematically in the municipalities; however, the steering committee held the responsibility to act as a 'Data and Safety Monitoring Board' (DSMB). The steering committee met regularly throughout the whole data collection period and monitored the safety based on reports of local experiences (without disclosing the group identity of cases). Thus, we applied no pre-specified guidelines for determining stopping rules due to a safety concern. The clinical

opinion from the steering committee deliberations did not reveal any *ad hoc* safety concerns.

Timing of the Final Analysis

The final analysis for the MMM vs. TAU comparison is planned to take place in two separate stages: (1) First, we will perform the statistical analyses based on all the repeated measures from baseline to 18 weeks; this will constitute the first main report of the trial to the advisory board and will be prepared for the MMM/TAU comparison when every randomized child has reached the 18-week assessment, and when the data for the primary endpoint should have been received and cleaned (anticipated to be August 6'st, 2019); (2) Longer-term (sustained effect) endpoints at 26 weeks for the MMM/TAU comparison will be analyzed when every child has reached the 26 weeks assessment and data for the primary endpoint have been received and cleaned (anticipated to be September 15'th, 2019). Data analysis for the cost-effectiveness outcomes will be performed at a later stage (estimated to take place in 2020) when the relevant information necessary for those analyses will be available from the Danish register: the Danish National Prescription Registry, the National Patient Register, and the Danish National Health Service Register.

Timing of Outcome Assessments

The schedule of study procedures is given in the table below. The expected visit dates and visit windows was defined as:

Variable View	x0	w0	w2	w4	w6	w8	w10	w12	w14	w18	w26
Sex	x										
Age	x										
Region	x										
Principal domain of problems	x										
Developmental delays	x										
School absenteeism	x										
DAWBA diagnosis	x										
Physical illness	x										
Living arrangement	x										
Parent registered as informant	x										
Mother's highest education	x										
Immigration history of parents	x										
Number of children in the household	x										
Mother's mental health problems	x										
Both parents had mental health problems	x										
Adherence 9-13 sessions MMM										x	
Strengths and Difficulties Questionnaire (SDQ impact score, parent reported)		x	x	x	x	x	x	x	x	x	x
*Spence Children's Anxiety Scale (SCAS, parent-reported)		x								x	x
*Mood and Feelings Questionnaire (MFQ. Parent-reported)		x								x	x
*Weiss Functional impairment rating Scale (WFIRS-P, parent reported)		x								x	x
*School attendance (percent school-days in last 4 weeks)		x								x	x
*Top-problem scores, parent-reported		x	x	x	x	x	x	x	x	x	x
*KIDSCREEN Physical Well-Being (self-reported, t-scores)		x								x	x
*KIDSCREEN Psychological Well-Being (self-reported, t-scores)		x								x	x
*Eyberg Child Behaviour Inventory (ECBI, intensity of problems)		x								x	x
*Eyberg Child Behaviour Inventory (ECBI, no of problems)		x								x	x
*Emotional and behavioral difficulties (SDQ total difficulties score, parent-reported)		x								x	x
Self-reported impact of mental health problems (SDQ impact score, range 0-10)		x	x	x	x	x	x	x	x	x	x
Self-reported emotional and behavioral difficulties (SDQ total difficulties score)		x								x	x
Self-reported anxiety reported (SCAS)		x								x	x
Self-reported depressive symptoms (MFQ)		x								x	x
Self-reported Top-problem scores		x	x	x	x	x	x	x	x	x	x
KIDSCREEN Autonomy & Parents (self-reported, t-scores)		x								x	x
KIDSCREEN Peers & Social Support (self-reported, t-scores)		x								x	x
KIDSCREEN School Environment (self-reported, t-scores)		x								x	x
Parent-reported Experience of Service Questionnaire (ESQ)										x	x
Self-reported Experience of Service Questionnaire (ESQ)										x	x
Teacher-reported impact of mental health problems (SDQ impact score, range 0-6)		x								x	x
Teacher-reported emotional and behavioral difficulties (SDQ total difficulties score)		x								x	x
KIDSCREEN Physical Well-Being (parent-reported, t-scores)		x								x	x
KIDSCREEN Psychological Well-Being (parent-reported, t-scores)		x								x	x
KIDSCREEN Autonomy & Parents (parent-reported, t-scores)		x								x	x
KIDSCREEN Peers & Social Support (parent-reported, t-scores)		x								x	x
KIDSCREEN School Environment (parent-reported, t-scores)		x								x	x
Parental Stress Scale (PSS)		x								x	x
Suicidality and negative cognitions binary composite score										x	x
Autonomy and social relationships binary composite score										x	x
Referral or contact to regional mental health services (parent-reported)										x	x

x: indicates that it was assessed/collected.

*: Confirmatory secondary outcomes presented in hierarchical order

Section 4: Statistical Principles

Confidence Intervals and *P* values

Level of statistical significance and use of confidence intervals: All applicable statistical tests will be 2-sided and will be performed using a 5% statistical significance level. All confidence intervals presented will be 95% and two-sided. We will not apply explicit adjustments for multiplicity, rather we will analyze the confirmatory secondary outcomes in a prioritized order (see "*gatekeeping procedure*" below). The analyses of the key secondary outcomes will be performed in sequence until one of the analyses fails to show the statistically significant difference, or until all analyses have been completed at a statistical significance level of 0.05. The key secondary statistical tests will be reported with *P* values for hypothesis tests and claims of statistical significance.

Adherence and Protocol Deviations

Adherence to the MMM intervention will be assessed based on the percent of participants who have completed 9-13 sessions of MMM ($n_{completed}$) out of all allocated to MMM ($n_{allocated}$). This outcome is defined as: % Adherence = ($n_{completed}/n_{allocated}$) *100%.

Other protocol deviations include active or passive refusal to continue to fill out the questionnaires (all participants were asked to continue to reply to questionnaires regardless of any type of drop out from treatment and/or referral to other mental health services).

Analysis Populations

The primary analyses will be based on the Intention to Treat (ITT) population, i.e., based on the Full Analysis Set. The ITT principle asserts the effect of a treatment policy (that is, the planned treatment regimen), rather than the actual treatment given (i.e., it is independent of treatment adherence). Accordingly, participants allocated to a treatment group (MMM or TAU, respectively) should be followed up, assessed and analyzed as members of that group, irrespective of their adherence to the planned course of treatment (i.e., independent of withdrawals and cross-over phenomena).

Section 5: Trial Population

Recruitment, Screening data, and Eligibility

Reporting of screening data will be used to describe the representativeness of the trial sample (Figure 1). Information to be included is depicted in the CONSORT flow diagram and the draft baseline characteristics table 1 below. The number of ineligible patients randomized, if any, will be reported, with reasons for ineligibility.

Help-seeking parents were invited to register their child in the local Pedagogical Psychological Services (in Danish '*Pædagogisk Psykologisk Rådgivning*', PPR) in their municipality.

A two-stage standardized screening for eligibility was implemented in the PPR:
Stage 1: The child and parent answered the strengths and difficulties questionnaire (SDQ), and if the SDQ-parent-scores were above the lower threshold for eligibility, the IT system automatically proceeded to administer the Spence Children's Anxiety Scale (SCAS), the Mood and Feelings Questionnaire (MFQ), and questions about child' and parents' characteristics (family constitution, health, social and school functioning). At this stage children were excluded, if problems were too mild for intervention. The lower threshold was based on parent-reported answers to the SDQ and an algorithm that combined scores of emotional and behavioral problems with functional impairments.
Stage 2: All children above the inclusion threshold were assessed with a clinical psychopathological interview by a trained psychologist in the PPR. The goal was to identify the mental health problems, formulate the Top-problem in the participants' own words, categorize the principal problem as 1) anxiety, 2) depressive symptoms or 3) behavioral problems - or detect a more severe mental health disorder in need of other treatment (see first exclusion criterion).

Finally, the PPR psychologist decided if the child was eligible according to the study inclusion criteria:

Inclusion criteria:

1) Aged 6–16 years and in 0-9th grade (excluding the second semester of the 9th grade).

2) SDQ scores reported by the parent are above the lower cutoff: a total difficulties score of ≥14 and/or emotional problems ≥5, and/or conduct score ≥3; combined with a functional impairment score of ≥1. Scores above this cutoff place the child's difficulties within the top 10 percent of mental health problems in the general age-matched population in Denmark.

3) The child and parents determine one top problem that has to fall within the domains of anxiety, depressive symptoms or behavioral problems, according to the classification by the PPR psychologist.

4) The child and at least one of the two parents understand and speak Danish sufficiently to participate in the treatment.

5) Written informed consent from the holders of the parental rights and responsibilities (usually both parents).

Exclusion criteria:

1) Indications that the child may have a severe mental disorder, like autism-spectrum disorder, attention-deficit/hyperactivity disorder (ADHD), schizophrenia-spectrum psychosis, eating disorder, severe obsessive-compulsive disorder, repeated self-harm, abuse or dependence of alcohol or psychoactive drugs, or other mental disorder requiring referral to a more intensive assessment or treatment within child and adolescent mental health services (after systematic assessment and according to usual recommendations and guidelines).
2) Indications of intellectual functional impairment, severe learning difficulties, or other special needs that would interfere negatively with the MMM training. The judgment is made as a best estimate by the PPR psychologist on the basis of the available information. A formal intelligence test is not required.
3) A prior diagnosis of any developmental or mental disorder after assessment by the regional child and adolescent psychiatrist, regardless of present status or treatment. A prior examination that did not result in a diagnosis of any specific mental health disorder will not exclude the child. The PPR psychologist must consult the study PI who decides whether there is sufficient information to exclude the child because of a significant prior psychiatric history.
4) Prior participation in the MMM pilot or current study.
5) The child and/or parents are unable to participate in weekly sessions throughout the next 13–18 weeks.

Enrolment procedures:

If the child was found eligible, the PPR psychologist asked the legal guardians (usually both parents) to give informed consent for their child's participation in the trial.

The PPR psychologist checked "yes" or "no" to each of the inclusion and exclusion criteria and uploaded the signed consent form to the database.

An automatic e-mail notified the research leader who reviewed the consent form and the answers to the inclusion and exclusion criteria before she approved the child for inclusion in the MMM study.

Once the child was enrolled, the database automatically sent an e-mail and SMS to the child and the parents with a link to the web-based baseline questionnaires.

The IT system automatically approved the child for randomization once the baseline data were collected. All standardized questionnaires had to be completed by each of the respondents.

Withdrawal/follow-up

Level of withdrawal, from intervention and/or from follow-up: *see draft Figure 1 below.* Timing of withdrawal/lost to follow-up data: *see draft Figure 1 below.* Reasons and details of how withdrawal/lost to follow-up data will be presented: see draft Figure 1 below.

Baseline Patient Characteristics

List of baseline characteristics to be summarized: *see draft Table 1 below.* Details on baseline characteristics will be summarized descriptively.

Section 6: Analysis

Outcome Definitions

The data assessment table (above), displaying the outcome measures collected at various time points, indicates how many data points will be part of the analysis. Also, Table 1 and Table 2 below define each outcome explicitly, clearly identifying primary and secondary variables. This display includes a clear specification of the hierarchical order (gatekeeping). Tables 1, 2, and 3 identify the specific measurement variable and its units (e.g., SDQ-impact scores) and provide descriptions and details of any data manipulations or derivations to be performed in the footnotes. If the calculation of a score is more complex, using a validated algorithm, then the algorithm will be provided in a footnote. Scoring, including handling of missing data items, will follow the procedure proposed by the instrument developers, which will be described and justified in the footnote. Sufficient detail will be provided in order for the reader to understand how the scores and results are to be calculated for each outcome.

Analysis Methods

Our primary analyses will be based on the ITT population, including all randomized participants with available data at baseline. Missing data will be handled indirectly and statistically modeled using repeated-measures linear mixed models (see below). These models will be valid if data are 'Missing at Random' (MAR): *"Any systematic difference between the missing values and the observed values can be explained by differences in observed data"*.[5] Contrasts between groups will be estimated based on repeated-measures analysis of covariance applied in mixed linear models (i.e., at 18 weeks from baseline).

The primary statistical model will consist of fixed effects and random effects. Fixed effects define the expected values of the observations, and random effects define the variance and covariances of the observations. In this study, participants were randomly assigned to two treatment groups (MMM vs TAU), and observations were made at nine time points for the primary outcome measure (i.e., at baseline and 2, 4, 6, 8, 10, 12, 14, and 18 weeks from baseline [see data collection table above]) for each participant. Basically, there are two fixed-effect factors: group and time. Random effects result from variation between

and within participants. We anticipate that measures on the same patient at different times are correlated, with measures taken closely together in time being more highly correlated than measures taken more apart in time; observations on different participants will be assumed as being independent.

The objectives of a repeated measures designs are to make inferences about the expected values of the observations, that is, about the means of the populations from which participants are sampled. This objective is achieved by taking into account treatment and time effects in the model. Data will be analyzed using SAS and R, with the particular outcome variable at baseline level as a covariate, using a multilevel repeated measures random effects model with participants as the random effect factor based on a restricted maximum likelihood (REML) model.

For continuous outcomes (e.g., SDQ-impact score) the change from baseline will be the response (dependent) variable, and the baseline value (one for each participant), treatment group (two levels), and time (nine levels) will be included as covariates, as well as the interaction between treatment group and time; Patient ID will be handled as a random effect. As the study design was based on a stratified randomization technique, we will also adjust for the three stratification variables. This statistical model holds all between-group comparisons at all assessment points up to 18 weeks from baseline (including baseline) and allows for evaluation of the average effect, as well as the trajectory over time from baseline to 18-week follow-up.

Categorical outcomes for dichotomous endpoints (including responder status and harms) will be analyzed with logistic regression based on a Generalized Linear Mixed Model with the same fixed effects and covariates as the respective analysis of covariance. Since Odds Ratios (*OR*s) for outcomes of common incidence either over- or under estimate the corresponding risk estimate, we will convert all the calculated *OR* values and 95% confidence intervals into approximate Risk Ratios (*RR*) in the text. This approach will also allow us to calculate numbers-needed-to-treat (NNTs) and numbers-needed-to-harm (NNHs) for efficacy and safety outcomes by dividing 1 by the risk difference that can guide clinicians in their decision making.

Missing Data and Sensitivity Analyses

Robustness is a concept that refers to the sensitivity of the overall conclusions to various limitations of the data, assumptions, and analytic approaches to data analysis. Robustness implies that the treatment effect and primary conclusions of the trial are not substantially affected when analyses are carried out based on alternative assumptions or analytic approaches.

Loss to follow-up and missing data for various reasons is difficult to avoid in randomized trials and in particular in pragmatic trials like the Mind-My-Mind trial. We will apply the analysis framework suggested by White et al (2011) in which missing data related to the ITT approach depend on making plausible assumptions about the missingness of the data and including all participants in subsequent sensitivity analyses[6]:

1. Attempt to follow up all randomized participants, even if they withdraw from allocated treatment (i.e., contact all individuals unless they explicitly stated that they had withdrawn their consent)

2. Perform a main analysis of all observed data that are valid under a plausible assumption about the missingness of the data (i.e., Model-based: data as observed; using linear mixed models, assuming that data are 'Missing at Random' [MAR])

3. Perform sensitivity analyses to explore the effect of departures from the assumption made in the main analysis (i.e., a non-responder-imputation: using the value at baseline to replace missing data will correspond to a non-responder imputation; these models will potentially be valid even if data are 'Missing Not At Random' [MNAR])

4. Account for all randomized participants, at least in the sensitivity analyses (covered by #2 and #3 above, plus the corresponding analyses based on the per protocol population).

The interpretation of the corresponding statistical measures of uncertainty of the treatment effect and treatment comparisons will involve consideration of the potential contribution of bias to the *p*-value, 95% confidence interval, and of the inference in general.

#1+2: Our primary analysis population will be all participants with available data at baseline, statistically modelled using repeated-measures linear mixed models (see above). These models will be valid if data are 'MAR'.

#3+4 Sensitivity: We will analyze all variables, with missing data being handled by multiple imputation techniques of the baseline level.

When the full analysis set, the per protocol set (defined as participants with available data at baseline, week 18, and/or week 26), and the analyses on the two different enrolment periods lead to essentially the same conclusions, confidence in the trial results is increased.

Additional Analyses

For erroneous reasons this trial included participants without appropriate confirmation in www.clinicaltrials.gov; 244 of 396 (61.6%) participants were unintentionally included before final trial registration was approved (and publicly available) in ClinicalTrials.gov. However, no data were analyzed before the entire study was completed and the database was locked. As a consequence, we will perform further exploration of the sensitivity of conclusions to the choice of the set of participants analyzed. In the Mind-My-Mind Trial, we will perform sensitivity analyses on two different enrolment (time) periods, corresponding to before (244 participants) and after registration (152 participants); the first mentioned period corresponds to approximately the first seven months of active inclusion.

Harms

Participants may experience some temporary discomfort as they learn new behaviors. Furthermore, some discomfort may be associated with answering questionnaires on psychopathology. However, due to the lack of evidence on potential harms related to psychological interventions in children and adolescents, the suicidality, negative cognitions, and poor quality of family relationships, free time and friendships will be monitored (see Table 4 below).

The trial data will be linked with data from the national registries on outpatient services, inpatient services, and emergency room visits to measure group-differences in service use related to acute crisis, self-harm and suicide attempts during the study period. The advantage of using register-based data to capture these rare events is the complete follow-

up of all included and randomized children, independent of any attrition from the study and from answering questionnaires. However, the annual update of the registries takes a full calendar year, so data will not be available before autumn 2020, and are therefore not part of the present study report.

Statistical Software

Analyses will be performed using SAS (proc mixed or proc glmm) and R version 3.5.0 (or newer R Project for Statistical Computing) with the packages lme4, nlme and emmeans.

Section 7: Manuscript Outline

Appendix 1: All protocol versions (merged); pdf format
Appendix 2: Statistical Analysis Plan; pdf format
Figure 1: Trial Flow Diagram

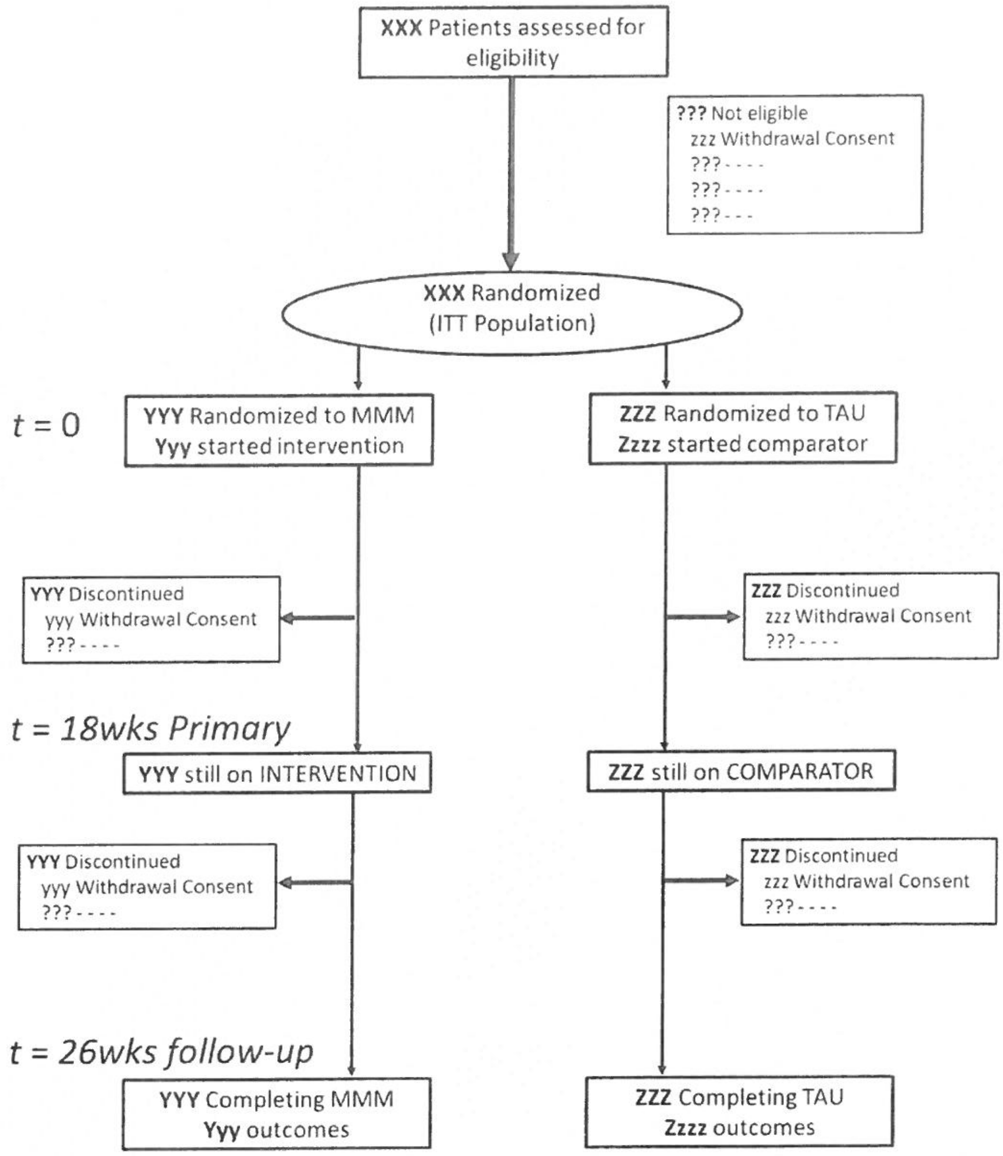

Table 1. Baseline Characteristics (The ITT Population)

Characteristic[1]	MMM group (n = ??)	TAU group (n = ??)	Total ($n = n_{MMM} + n_{TAU}$)
Demographic characteristics			
Girls, no. (%)			
Age, mean (SD), y			
Age-group:<br>6-10 y, no. (%)<br>11-16 y, no. (%)			
Region:<br>Holstebro-Helsingør, no. (%)<br>Vordingborg-Næstved, no. (%)			
Principal domain of problems:<br>Anxiety, no. (%)<br>Depressive symptoms, no. (%)<br>Behavioral problems, no. (%)			
Developmental delays:<br>Language, no. (%)<br>Any other[2], no (%)			
School absenteeism:<br>Above 4 weeks last y, no. (%)			
Number of DSM-IV/V Mental disorder based on DAWBA:<br>Anxiety disorder, no. (%)<br>Depressive disorder, no. (%)<br>Behavioral disorder, no. (%)<br>Neurodevelopmental disorder, no. (%)<br>Any disorder, no. (%)<br>Comorbidity, ≥ 2 disorders, no. (%)			
Physical illness (asthma, diabetes, eczema, epilepsy, other), no. (%)			
Living arrangement:<br>Both parents, no. (%)<br>Single parent, no. (%)<br>Other/reconstituted family, no. (%)			
Parent registered as informant:<br>Mother, no. (%)<br>Father, no. (%)			
Mother's highest education:<br>Elementary school, 9-10 y, no. (%)<br>High school/ skilled, 11-14 y, no. (%)<br>Bachelor and above, 15-17 y, no. (%)<br>Higher education, ≥17, no (%)			
Immigration history of parents:<br>Two parents born in Denmark, no. (%)<br>One foreign born, no. (%)<br>Two foreign born, no. (%)			
Number of children in the household:<br>only the index child<br>2, no. (%)<br>≥3, no. (%)			
Mother's mental health problems:<br>Anxiety, no. (%)<br>Depression, no. (%),<br>Other, no. (%)			
Both parents had mental health problems, no. (%)			
**Outcome measures**			
Primary outcome measure:			
SDQ Impact score (0-10)			
Key secondary outcome measures:			
Anxiety (SCAS, parent-reported; 0-114)			
Depressive symptoms (MFQ, parent reported; 0-68)			
Level of daily functioning of child (WFIRS, parent reported; 0-150)			
School attendance (percent school-days in the last 4 weeks; 0-100%)			
Top-problem score (parent reported; 1-10)			
KIDSCREEN-27, converted to T-values (SD)<br>Physical well-being<br>Psychological well-being			
Behavioral problems (ECBI, parent reported)<br>Intensity score (7-252)<br>Problem score (0-36)			

Emotional & behavioral problems (SDQ Total difficulties; 0-40)			

Abbreviations:
SDQ, The Strengths and Difficulties Questionnaire; SCAS, The Spence Children' s Anxiety Scale; MFQ, The Mood and Feelings Questionnaire, WFIRS-P, The Weiss Functional Impairment Rating Scale-Parent Reported; ECBI, The Eyberg Child Behaviuor Inventory; Kidscreen-27, a Health-Related Quality of Life (HRQOL) with five dimensions, of which we use the scales: Physical Well-Being (5 items), Psychological Well-Being (7 items). DAWBA: Development and Well Being Assessment, used for DSM-IV/V diagnoses.

[1] Data are presented as mean (SD) unless otherwise stated.

[2] Any other developmental delays include motor, social communication and learning difficulties.

Figure 2: Trajectory Least Squares Means scores over time for the child's impact of mental health problems reported by the parent (SDQ) from baseline to week 18, plus the extended follow-up (26 weeks).

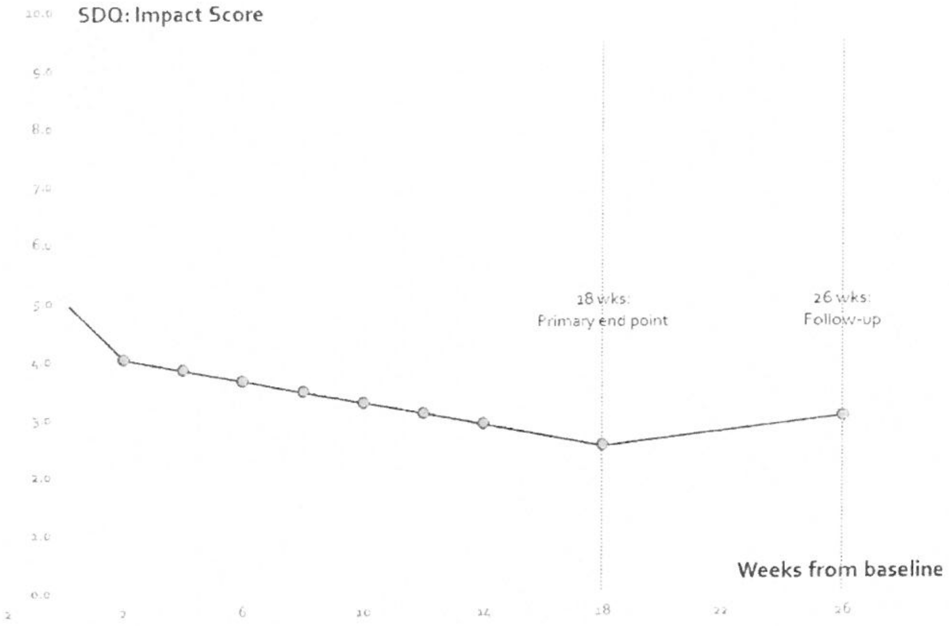

Table 2. Change from Baseline in Primary and Key Secondary Outcomes at 18 Weeks (The ITT Population)

**End Point**[1]	**MMM group (n = XXX)**	**TAU group (n = XXX)**	**Difference Between Groups (95% CI)**	***P* Value**
Primary outcome measure:				
Parent reported SDQ Impact score (0-10)				
Secondary outcome measures:				
Anxiety (SCAS, parent-reported; 0-114)				
Depressive symptoms (MFQ, parent reported; 0-68)				
Level of daily functioning of child (WFIRS, parent reported; 0-150)				
School attendance (percent school-days in the last 4 weeks, parent reported; 0-100%)				
Top-problem score (parent reported; 1-10)				
Self-reported KIDSCREEN-27, converted to T-values (SD)<br>Physical well-being<br>Psychological well-being				
Behavioral problems (ECBI, parent reported)<br>Intensity score (7-252)<br>Problem score (0-36)				
Emotional & behavioral problems (SDQ Total difficulties, parent reported; 0-40)				
Responder indices:				
Parent reported SDQ Impact score ≥ 1 point reduction from baseline, no. (%)				
Scoring below the parent reported SDQ inclusion cut-off[2], no. (%)				

Abbreviations:

SDQ, The Strengths and Difficulties Questionnaire; SCAS, The Spence Children's Anxiety Scale; MFQ, The Mood and Feelings Questionnaire, WFIRS-P, The Weiss Functional Impairment Rating Scale-Parent Reported; ECBI, The Eyberg Child Behavior Inventory; Kidscreen-27, a Health-Related Quality of Life (HRQOL) with five dimensions, of which we use the scales: Physical Well-Being, Psychological Well-Being.

[1] Data are presented as mean (SD) unless otherwise stated.

[2] Inclusion cut-off on the parent-reported SDQ: a total difficulties score of ≥14 and/or emotional problems ≥5, and/or conduct score ≥3; combined with a functional impairment score of ≥1.

Table 3. Exploratory Outcomes: Change from Baseline at 26 Weeks (The ITT Population)

End Point[1]	MMM group (n = XXX)	TAU group (n = XXX)	Difference Between Groups (95% CI)	*P* Value
Primary outcome measure:				
Parent reported SDQ Impact score (0-10)				
Secondary outcome measures:				
Anxiety (SCAS, parent-reported; 0-114)				
Depressive symptoms (MFQ, parent reported; 0-68)				
Level of daily functioning of child (WFIRS, parent reported; 0-150)				
School attendance (percent school-days in the last 4 weeks, parent reported; 0-100%)				
Top-problem score (parent reported; 1-10)				
Self-reported KIDSCREEN-27, converted to T-values (SD)<br>Physical well-being<br>Psychological well-being				
Behavioral problems (ECBI, parent reported)<br>Intensity score (7-252)<br>Problem score (0-36)				
Emotional & behavioral problems (SDQ Total difficulties, parent reported; 0-40)				
Responder indices:				
Parent reported SDQ Impact score ≥ 1 point reduction from baseline, no. (%)				
Scoring below the parent reported SDQ inclusion cut-off[2], no. (%)				

Abbreviations:
SDQ, The Strengths and Difficulties Questionnaire; SCAS, The Spence Children's Anxiety Scale; MFQ, The Mood and Feelings Questionnaire, WFIRS-P, The Weiss Functional Impairment Rating Scale-Parent Reported; ECBI, The Eyberg Child Behaviuor Inventory; Kidscreen-27, a Health-Related Quality of Life (HRQOL) with five dimensions, of which we use the scales: Physical Well-Being, Psychological Well-Being.

[1] Data are presented as mean (SD) unless otherwise stated.

[2] Inclusion cut-off on the parent-reported SDQ: a total difficulties score of ≥14 and/or emotional problems ≥5, and/or conduct score ≥3; combined with a functional impairment score of ≥1.

Table 4. Potential harms and negative outcomes at end of treatment (week 18), and at follow-up (week 26)

	MMM (n=XXX	TAU (n=XXX)	Difference Between Groups (95% CI)	*P* Value
**Potential harms measures**				
Primary measure of harms at week 18: Composite score of suicidality and/or negative cognition, no. (%)				
Primary measure of harms at week 26: Composite score of suicidality and/or negative cognition, no. (%)				
Secondary measure of harms at week 18: Composite score of poor quality of family relationships, free time and/or friendships, no. (%)				
Secondary measure of harms at week 26: Composite score of poor quality of family relationships, free time and/or friendships, no. (%)				
**Referrals to CAMHS**				
Parent-reported contact to CAMHS, from entry to week 18, no (%)				
Parent-reported contact to CAMHS, from entry to week 26, no (%)				

Abbreviation:
CAMHS, child and adolescent mental health services

Table 5. Other Exploratory Outcomes at 18 weeks and at 26 Weeks

End Point	*18 weeks*				*26 weeks*			
	N	MMM group (n = XX)	TAU group (n = XX)	Difference Between Groups (95% CI)	N	MMM group (n = XX)	TAU group (n = XX)	Difference Between Groups (95% CI)
Self-reported SDQ impact score (0-10)								
Self-reported SDQ total difficulties score (0-40)								
Self-reported anxiety (SCAS; 0-114)								
Self-reported depressive symptoms (MFQ; 0-66)								
Self-reported Top-problem score (1-10)								
KIDSCREEN Autonomy & Parents (self-reported, t-scores)								
KIDSCREEN Peers & Social Support (self-reported, t-scores)								
KIDSCREEN School Environment (self-reported, t-scores)								
Parent-reported Experience of Service Questionnaire (ESQ)								
Self-reported Experience of Service Questionnaire (ESQ)								
Teacher-reported SDQ impact score (0-6)								
Teacher-reported SDQ total difficulties score (0-40)								
KIDSCREEN Physical Well-Being (parent-reported, t-scores)								
KIDSCREEN Psychological Well-Being (parent-reported, t-scores)								
KIDSCREEN Autonomy & Parents (parent-reported, t-scores)								
KIDSCREEN Peers & Social Support (parent-reported, t-scores)								
KIDSCREEN School Environment (parent-reported, t-scores)								
Parental Stress in role functioning (PSS)								

Abbreviations:
SDQ, The Strengths and Difficulties Questionnaire; SCAS, The Spence Children's Anxiety Scale; MFQ, The Mood and Feelings Questionnaire, WFIRS-P, The Weiss Functional Impairment Rating Scale-Parent Reported; ECBI, The Eyberg Child Behaviour Inventory; Kidscreen-27, a Health-Related Quality of Life (HRQOL); PSS; Parental Stress Scale

Appendix Table 1. Change from Baseline in Primary and Key Secondary Outcomes at 18 Weeks (The ITT Population: missing data handled using multiple imputation)

End Point[1]	MMM group (n = XXX)	TAU group (n = XXX)	Difference Between Groups (95% CI)	*P* Value
Primary outcome measure:				
Parent reported SDQ Impact score (0-10)				
Secondary outcome measures:				
Anxiety (SCAS, parent-reported; 0-114)				
Depressive symptoms (MFQ, parent reported; 0-68)				
Level of daily functioning of child (WFIRS, parent reported; 0-150)				
School attendance (percent school-days in the last 4 weeks, parent reported; 0-100%)				
Top-problem score (parent reported; 1-10)				
Self-reported KIDSCREEN-27, converted to T-values (SD)<br>Physical well-being<br>Psychological well-being				
Behavioral problems (ECBI, parent reported)<br>Intensity score (7-252)<br>Problem score (0-36)				
Emotional & behavioral problems (SDQ Total difficulties, parent reported; 0-40)				
Responder indices:				
Parent reported SDQ Impact score ≥ 1 point reduction from baseline, no. (%)				
Scoring below the parent reported SDQ inclusion cut-off[2], no. (%)				

Abbreviations:
SDQ, The Strengths and Difficulties Questionnaire; SCAS, The Spence Children's Anxiety Scale; MFQ, The Mood and Feelings Questionnaire, WFIRS-P, The Weiss Functional Impairment Rating Scale-Parent Reported; ECBI, The Eyberg Child Behaviuor Inventory; Kidscreen-27, a Health-Related Quality of Life (HRQOL) with five dimensions, of which we use the scales: Physical Well-Being, Psychological Well-Being.

[1] Data are presented as mean (SD) unless otherwise stated.

[2] Inclusion cut-off on the parent-reported SDQ: a total difficulties score of ≥14 and/or emotional problems ≥5, and/or conduct score ≥3; combined with a functional impairment score of ≥1.

Appendix Table 2. Exploratory Outcomes at 26 Weeks (The ITT Population: missing data handled using multiple imputation)

End Point[1]	MMM group (n = XXX)	TAU group (n = XXX)	Difference Between Groups (95% CI)	*P* Value
Primary outcome measure:				
Parent reported SDQ Impact score (0-10)				
Secondary outcome measures:				
Anxiety (SCAS, parent-reported; 0-114)				
Depressive symptoms (MFQ, parent reported; 0-68)				
Level of daily functioning of child (WFIRS, parent reported; 0-150)				
School attendance (percent school-days in the last 4 weeks, parent reported; 0-100%)				
Top-problem score (parent reported; 1-10)				
Self-reported KIDSCREEN-27, converted to T-values (SD)<br>Physical well-being<br>Psychological well-being				
Behavioral problems (ECBI, parent reported)<br>Intensity score (7-252)<br>Problem score (0-36)				
Emotional & behavioral problems (SDQ Total difficulties, parent reported; 0-40)				
Responder indices:				
Parent reported SDQ Impact score ≥ 1 point reduction from baseline, no. (%)				
Scoring below the parent reported SDQ inclusion cut-off[2], no. (%)				

*Non-Responder Imputation: Defined as Baseline Observation Carried Forward.

Abbreviations:

SDQ, The Strengths and Difficulties Questionnaire; SCAS, The Spence Children' s Anxiety Scale; MFQ, The Mood and Feelings Questionnaire, WFIRS-P, The Weiss Functional Impairment Rating Scale-Parent Reported; ECBI, The Eyberg Child Behavior Inventory; Kidscreen-27, a Health-Related Quality of Life (HRQOL) with five dimensions, of which we use the scales: Physical Well-Being, Psychological Well-Being.

[1] Data are presented as mean (SD) unless otherwise stated.

[2] Inclusion cut-off on the parent-reported SDQ: a total difficulties score of ≥14 and/or emotional problems ≥5, and/or conduct score ≥3; combined with a functional impairment score of ≥1.

Appendix Table 3. Change from Baseline in the Primary and Secondary Outcomes at 18 Weeks: Per Protocol Population*

End Point[1]	MMM group (n = XXX)	TAU group (n = XXX)	Difference Between Groups (95% CI)	*P* Value
Primary outcome measure:				
Parent reported SDQ Impact score (0-10)				
Secondary outcome measures:				
Anxiety (SCAS, parent-reported; 0-114)				
Depressive symptoms (MFQ, parent reported; 0-68)				
Level of daily functioning of child (WFIRS, parent reported; 0-150)				
School attendance (percent school-days in the last 4 weeks, parent reported; 0-100%)				
Top-problem score (parent reported; 1-10)				
Self-reported KIDSCREEN-27, converted to T-values (SD)<br>Physical well-being<br>Psychological well-being				
Behavioral problems (ECBI, parent reported)<br>Intensity score (7-252)<br>Problem score (0-36)				
Emotional & behavioral problems (SDQ Total difficulties, parent reported; 0-40)				
Responder indices:				
Parent reported SDQ Impact score ≥ 1 point reduction from baseline, no. (%)				
Scoring below the parent reported SDQ inclusion cut-off[2], no. (%)				

*Only participants with data both at baseline and at week 18 are included in the analysis.

Abbreviations:
SDQ, The Strengths and Difficulties Questionnaire; SCAS, The Spence Children' s Anxiety Scale; MFQ, The Mood and Feelings Questionnaire, WFIRS-P, The Weiss Functional Impairment Rating Scale-Parent Reported; ECBI, The Eyberg Child Behavior Inventory; Kidscreen-27, a Health-Related Quality of Life (HRQOL) with five dimensions, of which we use the scales: Physical Well-Being, Psychological Well-Being.

[1] Data are presented as mean (SD) unless otherwise stated.

[2] Inclusion cut-off on the parent-reported SDQ: a total difficulties score of ≥14 and/or emotional problems ≥5, and/or conduct score ≥3; combined with a functional impairment score of ≥1.

References

1. Costello EJ, Copeland W, Angold A. Trends in psychopathology across the adolescent years: What changes when children become adolescents, and when adolescents become adults? J Child Psychol Psychiatry. 2011;52(10):1015-1025. doi:10.1111/j.1469-7610.2011.02446.x
2. Copeland WE, Adair CE, Smetanin P, et al. Diagnostic transitions from childhood to adolescence to early adulthood. J Child Psychol Psychiatry. 2013;54(7):791-799. doi:10.1111/jcpp.12062
3. Reef J, Van Meurs I, Verhulst FC, Van Der Ende J. Children's problems predict adults' DSM-IV disorders across 24 years. J Am Acad Child Adolesc Psychiatry. 2010;49(11):1117-1124. doi:10.1016/j.jaac.2010.08.002
4. Arango C, Díaz-Caneja CM, McGorry PD, et al. Preventive strategies for mental health. The Lancet Psychiatry. 2018;5(7):591-604. doi:10.1016/S2215-0366(18)30057-9
5. Detry MA, Ma Y. Analyzing Repeated Measurements Using Mixed Models. Jama. 2016;314(4):407-408. doi:10.1001/jama.2015
6. White IR, Horton NJ, Carpenter J, Pocock SJ. Strategy for intention to treat analysis in randomised trials with missing outcome data. BMJ. 2011;342:d40. doi:10.1136/bmj.d40

Summary of amendments of the study protocol

Protocol Version	Date	Revisions
Protocol version 1.2, 2017-18-04	18th of April 2017	Original version approved by the local scientific ethics committee (Journal nr.:H-17011408)
Protocol version 1.2, with extension 2	4th of December 2017	Site changes: one additional municipality (Helsingoer) was added to improve the recruitment rate by conducting the trial in four municipalities instead of three (see table 9 and 27 in the final protocol). The estimated sample size (412 included children and 309 children with follow-up data, assuming 25% attrition) was not changed.
Protocol version 1.2, with extension 3	17th of May 2018	The inclusion period was extended with 1½ months, now ending at 31st of December 2018 instead of 15th of October 2018. The schedule for the recruitment rate in the municipalities was adjusted in accordance with the extension.
Final protocol was version 1.2, with extension 4: In Danish: "Protocol version 1.2 med tillæg 2019-27-03"	27th of March 2019	The follow-up period was extended with two months, now ending at 31st of August 2019 instead of 30th of June 2019. A total of 396 children had been included and randomized by the end of the inclusion period in December 2018. The recruitment period was not extended, because the observed attrition rate was much lower than expected. Hence, we expected to have enough children with follow-up data, after adjusting the sample size from 412 to 396 included children.
