# Supplementary material for: Effectiveness of Transdiagnostic Cognitive-Behavioral Psychotherapy Compared With Management as Usual for Youth With Common Mental Health Problems: A Randomized Clinical Trial
Source: JAMA Psychiatry. 2020 Dec 23;78(3):1–12. doi: 10.1001/jamapsychiatry.2020.4045 (PMC7758821; doi:10.1001/jamapsychiatry.2020.4045)
Supplement: Supplement 2. — eMethods 1. Steering Committee eMethods 2. Literature Overview eMethods 3. Interventions in Mind My Mind (MMM) eMethods 4. Management as Usual (MAU) eFigure 1. The Prototypical Program for Anxiety in MMM eFigure 2. The Prototypical Program for Depressive Symptoms in MMM eFigure 3. The Prototypical Program for Parent Training in MMM eFigure 4. Scatterplot of the Correlation Between the Number of Sessions and the Primary Outcome Measure at 18 weeks, in the MMM Intervention Group (Observed Values) eTable 1. Potential Harms and Negative Outcomes at 18 Weeks and at 26 Weeks (the ITT Population) eTable 2. Other Exploratory Outcomes: Change from Baseline at 18 Weeks and 26 Weeks (the ITT Population) eTable 3. Change From Baseline in Primary and Secondary Outcomes at 18 Weeks in the ITT Population: Missing Data Handled Using Multiple Imputation eTable 4. Exploratory Outcomes: Change from Baseline at 26 Weeks in The ITT Population: Missing Data Handled Using Multiple Imputation eTable 5. Change From Baseline in Primary and Secondary Outcomes at 18 Weeks: Per Protocol Population eTable 6. Change From Baseline in Primary and Secondary Outcomes at 18 Weeks: Before Trial Registration eTable 7. Change From Baseline in Primary and Secondary Outcomes at 18 Weeks: After Trial Registration eTable 8. Correlation Matrix Exploring the Intercorrelations of the Primary and Key Secondary Outcomes (Change Scores) in the MMM Group eTable 9. Correlation Matrix Exploring the Intercorrelations of the Primary and Key Secondary Outcomes (Change Scores) in the MAU Group eTable 10. Subgroup Analysis for the Observed Parent-Reported SDQ Impact Scores (n = 343) eReferences. [file jamapsychiatry-e204045-s002.pdf]

Supplementary Online Content

Jeppesen P, Wolf RT, Nielsen SM, et al. Effectiveness of transdiagnostic cognitive-behavioral psychotherapy compared with management as usual for youth with common mental health problems: a randomized clinical trial. *JAMA Psychiatry.* Published online December 23, 2020. doi:10.1001/jamapsychiatry.2020.4045

eMethods 1. Steering Committee
eMethods 2. Literature Overview
eMethods 3. Interventions in Mind My Mind (MMM)
eMethods 4. Management as Usual (MAU)
eFigure 1. The Prototypical Program for Anxiety in MMM
eFigure 2. The Prototypical Program for Depressive Symptoms in MMM
eFigure 3. The Prototypical Program for Parent Training in MMM
eFigure 4. Scatterplot of the Correlation Between the Number of Sessions and the Primary Outcome Measure at 18 weeks, in the MMM Intervention Group (Observed Values)
eTable 1. Potential Harms and Negative Outcomes at 18 Weeks and at 26 Weeks (the ITT Population)
eTable 2. Other Exploratory Outcomes: Change from Baseline at 18 Weeks and 26 Weeks (the ITT Population)
eTable 3. Change From Baseline in Primary and Secondary Outcomes at 18 Weeks in the ITT Population: Missing Data Handled Using Multiple Imputation
eTable 4. Exploratory Outcomes: Change from Baseline at 26 Weeks in The ITT Population: Missing Data Handled Using Multiple Imputation
eTable 5. Change From Baseline in Primary and Secondary Outcomes at 18 Weeks: Per Protocol Population
eTable 6. Change From Baseline in Primary and Secondary Outcomes at 18 Weeks: Before Trial Registration
eTable 7. Change From Baseline in Primary and Secondary Outcomes at 18 Weeks: After Trial Registration
eTable 8. Correlation Matrix Exploring the Intercorrelations of the Primary and Key Secondary Outcomes (Change Scores) in the MMM Group
eTable 9. Correlation Matrix Exploring the Intercorrelations of the Primary and Key Secondary Outcomes (Change Scores) in the MAU Group
eTable 10. Subgroup Analysis for the Observed Parent-Reported SDQ Impact Scores (n = 343)
eReferences.

This supplementary material has been provided by the authors to give readers additional information about their work.

©2020 Jeppesen P et al. *JAMA Psychiatry*.

eMethods 1. Steering Committee

The steering committee was responsible for the study's overall management. The members of the steering committee were delegates from the four participating municipalities, the three regional child and adolescent mental health services (the Zealand Region, the Capital Region of Denmark, and the Central Denmark Region), and the nongovernmental organization (Psykiatrifonden) that was responsible for the implementation of the MMM program in the municipalities:

Kitt Boel (leader of Educational-Psychological Advisory Services [Danish abbreviation: PPR], in Naestved, the Zealand Region, Denmark)

Steen Rønne (Leader of PPR in Vordingborg, the Zealand Region, Denmark)

Finn Brunberg (Leader of PPR in Helsingoer, the Capital Region of Denmark)

Steen Fredriksen (Leader of PPR in Holstebro, the Central Denmark Region)

Anders Hede (Head of Research, TrygFonden)

Ida Hagemann (Project Manager, TrygFonden)

Marianne Skjold (CEO, Psykiatrifonden)

Torsten Bjørn Jacobsen (Chairman, Psykiatrifonden)

Anne Lindhardt (Former Chairman, Psykiatrifonden)

Birthe Wielandt Houe (Project Manager, Psykiatrifonden)

Pia Jeppesen (Principal Investigator, the Capital Region of Denmark)

Per Hove Thomsen (Professor, the Central Denmark Region).

Anne Katrine Pagsberg (Professor, the Capital Region of Denmark)

Niels Bilenberg (Professor, the Region of Southern Denmark)

Katrine Bærentzen (Clinical Leader, the Capital Region of Denmark)

Jesper Pedersen (Chief Physician, the Zealand Region, Denmark)

Elsebeth Vesterheden (Chief Nurse, the Central Denmark Region)

©2020 Jeppesen P et al. *JAMA Psychiatry*.

The steering committee also acted as the data and safety monitoring board. The steering committee met regularly throughout the study period and monitored the study's safety based on reports of local experiences without disclosing the allocated treatment group identity of cases.

Psykiatrifonden was responsible for the implementation of the MMM program, including training organization and therapist supervision, as well as video tool use and Web-based data collection. The trial was conducted in accordance with the principles of the Declaration of Helsinki.

©2020 Jeppesen P et al. *JAMA Psychiatry*.

eMethods 2. Literature Overview

We conducted a systematic literature search in the Ovid MEDLINE, EMBASE, and Psychinfo databases focusing on systematic reviews and meta-analyses of the efficacy of cognitive and behavioral therapies (CBT) for children and adolescents with anxiety, depressive symptoms/disorders, or behavioral problems/disorders. The search covered the period from the start of the databases. It was first performed in 2014 and subsequently updated in 2016 and on December 11th, 2019. The first author (Pia Jeppesen) screened and assessed the publications (899 hits in 2019) to select systematic reviews and meta-analyses fulfilling the following criteria:

1. Systematic reviews and/or meta-analyses of randomized controlled trials (RCTs) and cluster-randomized trials of a CBT intervention targeting anxiety, depressive symptoms/disorders, or behavioral problems/disorders in youth (younger than 18 years) that compare the experimental intervention with a control condition (e.g., waitlist control group, treatment as usual, attention control, or an active psychological intervention).
2. Reviews that assess and report the quality of the included studies to help readers assess the strength of the evidence in the review.

The systematic literature search and our reading of selected papers (see below) provided us with an overview of the evidence for the effects of CBT for anxiety, depressive symptoms and disorders, and behavioral problems and disorders in youth. Inspired by the distillation process described by Chorpita et al.,[1] we used this overview of the evidence base to select well-documented programs for early and preventive intervention of emotional and behavioral problems in youth. Finally, we listed the common CBT methods and techniques across the disorder-specific programs. The Mind My Mind (MMM) program (see Supplementary Appendix, Intervention – Mind My Mind) was developed based on clinical experience combined with our comprehensive overview of the evidence base.

Here, we briefly discuss the current evidence base for problem- and disorder-specific CBT that targets anxiety, depressive symptoms and disorders, or behavioral problems and disorders in youth based on our literature search as of December 11th, 2019. In general, the systematic reviews and meta-analyses assessed the quality of the evidence as low to moderate due to small samples, poor methodology, heterogeneity of results, and the risk of publication biases.

©2020 Jeppesen P et al. *JAMA Psychiatry*.

Evidence for the Effectiveness of CBT for Anxiety in Youth

The effects of CBT on children and adolescents with anxiety are documented in several systematic reviews and meta-analyses.[2–8] These studies proved the beneficial effects of CBT compared to a wait list control (WLC) on the rate of remission of anxiety diagnoses and on measures of psychopathology. The latest update of the Cochrane review (41 studies, 1806 participants)[7] examined an average of 13 weekly sessions of CBT compared to WLC and found significant beneficial effects on the remission rate (59% versus 15%, OR 7.85, number needed to treat [NNT]=3) and the reduction of anxiety symptoms. Apparently, there were no moderating effects of age, gender, or severity and comorbidity of the anxiety.

A recent network meta-analysis (101 studies, 6625 participants)[9] of the efficacy and acceptability of various types of psychotherapy for acute anxiety disorders in children and adolescents found that most formats of CBT and behavioral therapies (i.e., CBT without cognitive restructuring) were significantly more effective than WLC in reducing anxiety symptoms. The median number of sessions was 12; these were typically scheduled once per week. Group-based CBT was the only intervention that was significantly more effective than most other psychotherapies and control conditions in reducing posttreatment anxiety symptoms. It was likewise more effective than some psychotherapies and all control conditions after a short-term follow-up. Therefore, in terms of posttreatment efficacy, the most effective treatments were group CBT and group behavioral therapy. There was a non-significant difference by age pointing toward group CBT for adolescents and group behavioral therapy for children. However, regarding the efficacy after a median follow-up period of 6 months (range 1–12 months posttreatment), the most effective treatments were parent-only CBT and individual behavioral therapy with parental involvement. In terms of health-related quality of life and functioning, almost all CBT, but not behavioral therapy, was significantly more beneficial compared to psychological placebo and WLC. The results[9] indicated that group CBT might be the initial choice of psychotherapy for anxiety disorders in children and adolescents. More research is needed to make clear recommendations regarding age- and disorder-specific treatment. Furthermore, there were indications of inflated estimates of the effects of CBT when compared with WLC[9].

©2020 Jeppesen P et al. *JAMA Psychiatry*.

Comorbidity of anxiety with depression and conduct disorder is frequent and usually predicts an overall worse course during CBT treatment for anxiety compared to no comorbidity,[10,11] and even though both anxiety and depressive symptoms are reduced during CBT for anxiety,[11,12] a better treatment of comorbid conditions is warranted.

Although there is no clear evidence of additive beneficial effects of parental involvement in youth CBT, there is a clear rationale for the involvement of parents as support for the child (according to the developmental needs of the child) and as co-agents of change. Because most parents naturally try to protect their children from unpleasant situations and feelings, they may accidentally contribute to maladaptive patterns of avoidance behavior, thereby reinforcing the child's anxiety.[13] An important goal of parental involvement in CBT is therefore to make the parents aware of the maladaptive responses and teach them better ways to support their child in the sessions and during homework in which the child learns to apply more adaptive and realistic ways of thinking and acting. A study of various types of parental involvement [14] found that the active training of parents in contingency management and the transfer of control to parents were associated with better long-term effects.[14]

A recent meta-analytic study[15] of CBT for internalizing disorders (anxiety, depression, posttraumatic stress disorder, and obsessive-compulsive disorder) in children and adolescents included 76 RCTs testing 106 CBT programs. The results indicated that parental involvement was significantly associated with larger pretreatment to posttreatment and pretreatment to follow-up effect sizes. The results indicate that parental involvement may help maximize the long-term effectiveness of youth CBT. This is also in line with the findings of the network meta-analysis [9] in which parent-only CBT and individual behavioral therapy with parental involvement produced the highest efficacy at long-term follow-up.

A comparison of CBT programs with and without booster sessions[16] found that CBT programs with booster sessions were associated with significantly better effects. In contrast, a recent meta-analytic study[15] of CBT for internalizing disorders found no significant associations between the use of booster sessions, goal setting, and maintenance/relapse prevention in CBT on one hand and the effect sizes at post-treatment or follow-up on the other hand.

In conclusion, CBT is established as an effective psychological treatment for anxiety disorders in children and adolescents across a range of ages, co-morbidities and delivery formats. No clear evidence indicates that one way of providing CBT is more effective than another, but

parental involvement may help maximize the long-term effectiveness of CBT for internalizing conditions in children and adolescents. There is no evidence for the long-term efficacy of youth CBT beyond 12 months after the end of treatment.

The most well-documented CBT programs for the treatment of anxiety disorders in children and adolescents are Coping Cat[17], which was developed into a brief version[18], and Cool Kids.[19] The effect of Cool Kids was tested in Denmark and demonstrated significant positive effects when delivered as group CBT with parental involvement and compared to WLC.[20]

Evidence for the effectiveness of CBT for Depressive Symptoms and Disorders in Youth

The effectiveness of CBT for the prevention and treatment of subclinical and milder clinical levels of depressive disorders in youth was studied in several systematic reviews and meta-analyses of RCTs and cluster-randomized trials.[3,6,21–30]

The Cochrane review and meta-analysis of preventive psychological interventions[25] included 83 trials, of which 67 were carried out in school settings, eight in colleges or universities, and four in clinical settings. The preventive psychological interventions included CBT, interpersonal therapy (IPT) and third-wave CBT, which were compared with no intervention or with an attention placebo when available. Most interventions were delivered in a group format. The primary outcome was depression diagnosis at follow-up, which assessed up to 12 months after the intervention. The comparison of intervention versus no intervention (32 RTCs, 5965 participants) showed a small statistically significant effect (a depression rate of 17% versus 19%, NNT=33). There were also small positive benefits associated with the psychological depression prevention programs measured as symptom reduction. However, when the interventions were compared with an attention placebo control, there were no effects. This was supported by a recent review[31] that found little evidence to support school-based interventions for universal and targeted prevention of depression or anxiety.

In terms of psychotherapy for the treatment of child and adolescent depression, the number of trials is relatively small, and the evidence is limited. A network meta-analysis[24] investigated the comparative efficacy and acceptability of psychotherapies for depression in children and

adolescents (52 RCTs, 3805 participants) of nine psychotherapies and four control conditions. At post-treatment, only IPT and CBT were significantly more effective than most control conditions, and only IPT and CBT were more effective than play therapy. At follow-up, IPT and CBT were significantly more effective than most control conditions, and IPT and CBT were also superior to problem-solving therapy. Regarding acceptability, IPT and problem-solving therapy had significantly fewer all-cause discontinuations than CBT. The authors concluded[24] that IPT and CBT are the best available psychotherapies for depression in children and adolescents. Furthermore, the results indicated that use of WLC may have inflated the effect of the psychotherapies that were investigated in the network meta-analysis.

A 2006 meta-analysis[28] of the effects of psychotherapy for depression in children and adolescents found modest benefits associated with psychotherapy to treat depression. The mean effect size (0.34) was inferior compared to the mean effect size for CBT treatment of anxiety. Furthermore, CBT fared no better than other approaches, and effects were not maintained after long-term follow-up. A recent update[26] included 55 RCTs of psychotherapy versus a control condition for youth depression. This meta-analysis found beneficial effects of active therapy (mean effect size 0.36) at posttreatment, and at follow-up (mean effect size 0.21) at an average of 42 weeks posttreatment. The effect sizes were significantly larger for interpersonal therapy compared to CBT. The effects of psychotherapy showed some specificity for depressive symptoms that were reduced more than measures of anxiety and externalizing behavior. The authors concluded [26] that the evidence base for psychotherapy, including CBT, for youth depression is limited.

An exploratory meta-analysis[29] found insufficient evidence to determine the active components of CBT for depression, but programs with consistent beneficial effects seemed to focus on cognitive restructuring skills and problem solving.[29] In contrast, Weisz et al.[28] found evidence pointing toward greater effects for CBT with a strong focus on behavioral methods (e.g., behavioral activation) relative to the focus on cognitive restructuring. Behavioral activation is aimed at increasing the engagement in nurturing activities and thereby creating opportunities for the individual to experience positive affect.[32] Behavioral activation is an evidence-based treatment for depression in adults with extensive research supporting its effectiveness. So far, few studies have focused on behavioral activation as a stand-alone treatment in youth.[33] These studies do, however, show promising reductions in depressive symptoms following behavioral activation,[33,34] so behavioral activation may thus be effective when delivered as a stand-alone treatment or integrated into CBT for depression in.

©2020 Jeppesen P et al. *JAMA Psychiatry*.

Regarding suicidality, a Cochrane review and meta-analysis[30] of psychological treatment versus antidepressant medication demonstrated a significant reduction in the incidence and severity of suicidal ideation with psychological treatment (mainly CBT) compared to drug treatment.

Very few studies have investigated the effects of CBT on preadolescent children with depression. A meta-analysis[21] focused on CBT for depressive symptoms in children with a mean age below 13 years (10 RCTs, 523 participants) and found significant effects of CBT compared to control conditions (WLC or active placebo), with larger effects in older studies, among older children, and with higher numbers of sessions. A more recent meta-analysis[22] of psychological treatments for depression in the same age group found inconclusive evidence for the effectiveness of all psychological treatments of depression in pre-adolescent children. The study included, however, only seven studies, of which only three were CBT studies of good quality.

In conclusion, there is evidence to support CBT as an effective treatment for depressive symptoms and disorders in mature children and adolescents, but the evidence is less clear for preadolescent children. The mean effect sizes are modest and significantly smaller than the effect sizes reported for CBT for youth anxiety disorders. CBT may be superior to psychopharmacological treatments of depression in terms of improved safety and reduced suicidal ideation. There is insufficient evidence to support school-based interventions for universal and targeted prevention focusing on anxiety and depressive symptoms.

The evidence-based CBT programs for the treatment of depressive symptoms and disorders in children and adolescents include the manual used in the Treatment of Adolescents with Depression Study,[35] the Primary and Secondary Control Enhancement Therapy,[36] the Penn Resiliency Program,[37] and the ACTION treatment program.[38] There are several ongoing studies of transdiagnostic CBT programs indicated for the prevention and treatment of anxiety and depressive symptoms in Norway, such as the transdiagnostic EMOTION intervention for children ages 8–12.[39]

Evidence for the Effectiveness of Parent Training and CBT for Behavioral Problems and Disorders in Youth

There are three groups of evidence-based interventions for behavioral problems and disorders (including violence, aggression, and disruptive behavior) in children and adolescents:

1. Parent training programs aimed at helping parents develop their parenting skills, communication, and contact with their child and to reinforce desired behaviors.
2. School-based CBT prevention programs for behavioral problems delivered individually or in groups with and without parental involvement and aimed at improving the child's social communication skills and anger management skills.
3. Programs that combine parent training and child psychotherapy.

Group-based parent training programs for parents of children with problem behaviors or conduct disorders have been extensively investigated. A Cochrane review and meta-analysis[40] compared group-based parent training programs for parents of children ages 3–12 with no treatment or WLC and found significant reductions in child conduct problems in the intervention group whether the changes were assessed by parents or by an independent, blinded assessor. The experimental intervention was also associated with significant improvements in positive parenting skills, as well as reductions in negative or harsh parenting practices, based on the parent and independent observer's reports. There were also small but significant effects on the parents' mental health. Moreover, economic evaluations of the intervention compared to WLC indicated that the cost of bringing the average child with clinically significant levels of conduct problems into the non-clinical range was only EUR 2217 per family. The severity of the child's behavioral problems and the family's socioeconomic status did not moderate the treatment effects.[40]

The evidence for school-based indicated prevention programs for children and adolescents when delivered individually or in groups and with or without the involvement of parents have been documented in systematic reviews and meta-analyses.[41,42] The studies found significant positive effects of the school-based programs on behavioral problems, as well as on social skills, social cognition, and adaptation when compared to no intervention. Programs with a strong focus on adaptive social skills training led to greater effects than did programs with a strong focus on anger management.[43] Moreover, lengthy and more complex programs showed lesser effects than brief, more focused programs.[41] Some authors found that individual treatment had a greater impact than group treatment,[43] whereas others authors did not.[44] It seemed to be important that parents and teachers agreed on the need for intervention, as outcomes were significantly worse if only teachers recognized the child's behavioral problems.

A meta-analysis of CBT for behavioral problems in children with externalizing disorders (Attention Deficit Hyperactivity Disorder and Oppositional Defiant Disorder)[45] focused

on interventions comprising parent training and child psychotherapy based on CBT-methods. The intervention was compared with WLC or self-help assistance for parents. The study's results indicated significant effects on children's disruptive behaviors, social skills, and parental distress. Small reductions in problem internalization, aggression, and maternal depressive symptoms were also seen.[45] This evidence suggested the beneficial effects of combined parent training and child psychotherapy for externalizing problems.

In conclusion, parent training programs with and without child psychotherapy (i.e., social and communication skills training) have demonstrated beneficial effects on a broad spectrum of symptoms and functioning in children and parents. However, parent training may not be developmentally appropriate in late adolescence, and the effects of parent training have only been documented for children up to age 13.[40,46] The beneficial effects of child psychotherapy increased with child age and maturity.[47]

The evidence-based programs for parent training are widely disseminated and include the Incredible Years (IY),[48] the Oregon Model of Parent Management Training (PMT-O),[49] and the Triple-P[50] program. The Problem Solving Skills Training[51] program is a combined program that targets parents and youth, whereas Coping Power[52,53] and its Dutch adaptation, Alles Kidzzz[44,54] target only youth, both individually or in groups. IY and the PMT-O are used in Danish municipalities.

eMethods 3. Interventions in Mind My Mind (MMM)

Rationale for Transdiagnostic and Modular CBT

Despite evidence for the beneficial effects of CBT on anxiety, depressive symptoms and disorders, and behavioral problems and disorders in youth, there is a lack of implementation and dissemination of such evidence-based treatments in everyday clinical practice.

These persistent gaps between knowledge and practice call for initiatives to overcome the barriers related to implementing evidence-based programs to address common mental health problems in youth. Important challenges include the broad spectrum of common mental health

problems, and the high rate of co-occurring and fluctuating problems in youth. Timely delivery of quality treatment requires the implementation of several disorder-specific programs—each supported by a system that identifies the target group, trains and supervises the therapists, and monitors the treatment effects. Frequent staff turnover in many health services adds to the complexity and expense of this model.

The challenges of implementing several disorder-specific treatment programs have inspired the development and evaluation of transdiagnostic treatment approaches that address multiple problem areas and enable more flexible adaptation to the needs of individual children and families, and thereby increase transferability to various care settings while retaining the benefits of evidence-based and manualized treatments.[55,56]

The transdiagnostic treatment approaches have the potential to achieve the following:

- Target the comorbidity and fluctuation of psychopathology in youths by tailoring treatment to the individual child.
- Target common key mechanisms of pathology.
- Minimize training demands and costs for clinicians.
- Facilitate the large-scale implementation of evidence-based treatment by targeting a large group of youth.

There are basically two different transdiagnostic approaches to the delivery of CBT for youth:[55]

1. A unified approach that targets underlying and common dysfunctions across multiple psychopathologies (e.g., cognitive inflexibility and emotion regulation) to reduce avoidance and other maladaptive behaviors.
2. A modular approach comprising *problem-specific and generic modules* along with decision rules (e.g., guided by flowcharts) that determine the dosing of modules to tailor the treatment to the individual child and family.

The implementation and evaluation of transdiagnostic treatment programs for youths is still in its early stages, but there are promising results from studies of the unified[57,58] and modular approaches[59,60] to the implementation of evidence-based CBT for youths.

The rationale for the development of a new transdiagnostic and modular manual in Denmark was the need for large-scale implementation of evidence-based psychological interventions to a

very heterogenous group of youth in a non-specialist community care setting. The transdiagnostic and modular approach made it possible to bring together various evidence-based interventions targeting a broad range of mental health problems and disorders into one manageable “package” of interventions that could be “transported” into this type of setting. In other words: the primary goal was to design a single package of multiple interventions for multiple classes of problems/disorders to “speed up” the dissemination of an evidence-based practice in youth mental health prevention and intervention.

The first empirically supported transdiagnostic and modular intervention for youth with emotional and behavioral problems is the Child “STEPs” using the “Modular Approach to Therapy for Children with Anxiety, Depression, Trauma, or Conduct Problems” (MATCH-ADTC).[60,61] The MMM manual was inspired by the MATCH-ADTC, but our aim, content and structure differ in several aspects. Both manuals are transdiagnostic and modular and include somewhat similar evidence-based behavioral and cognitive behavioral (CBT) processes, methods and techniques for treating different types of anxiety, depressive symptoms and behavioral problems. However, the MMM manual was designed for indicated prevention and treatment of emotional and behavioral problems in help-seeking youths in a non-specialist school-based setting, whereas the MATCH-ADTC targeted children who were clinically referred for treatment in a community mental health setting.

Content of the Transdiagnostic and Modular Treatment in MMM

The Mind My Mind program (MMM) is a transdiagnostic and modular intervention for indicated prevention and early treatment of children aged 6-16 years with emotional and/or behavioral problems below the threshold for psychiatric referral. The treatment manual contains evidence-based CBT methods and techniques.

The MMM manual prescribes 9-13 sessions of individual training delivered in 17 weeks or less, with a booster delivered 6-8 weeks after end of treatment, whereas the MATCH-ADTC manual allow for flexible adaptation of the duration of the treatment, which resulted in a mean duration of approximately 30 weeks (16 sessions) in the first effectiveness trial.[60] Unlike MATCH-ADTC, the MMM intervention was not designed to treat trauma as a primary problem.

The MMM manual is a book of 323 pages, including 73 worksheets and 3 flowcharts. The treatment manual was designed for delivery by psychologists with very limited education and training in manualized CBT. To this end, instructions are comprehensive yet easy to read. The content is organized into generic and problem-specific modules. Each module is presented in a fixed format starting with an overview of aims, materials, processes and methods, followed by detailed instructions of when and how to apply the methods and techniques. Furthermore, examples of the dialogue between the therapist and the participants are integrated throughout the text, key principles are highlighted in boxes, and case-vignettes illustrate different training courses. Finally, the MMM manual is packed and delivered to the municipality together with a fully integrated program for education and supervision of the psychologists, and a database for standardized recruitment, assessment and monitoring of youth outcomes.

Despite the implementation support, the modular approach can be challenging to apply for the therapists. The therapist may experience a constant pressure to make decisions about the focus of the therapy, i.e. what are is the current problems, which one is more important to focus on, and when should the focus change? The MMM training begins with the formulation of a “Top-problem” as a starting point for the case formulation, the setting of goals, and the monitoring of outcome. The MMM manual guides the therapist to explore the problems in a collaborative process with the youth and parent, by which they reach a common understanding of the problem, or the set of problems that currently drive the distress and impairment and that motivate them to seek help. In case of multiple co-occurring problems, the MMM manual still encourages the formulation of one single core problem (called Top-problem) that is written down, using the own words of the youth/parent. The nature of the Top-problem defines which main course (see Flowcharts in Figure S1, S2 and S3) to start with.

The child is the primary recipient of therapy within the main programs for anxiety and depressive symptoms, whereas in the case of a child with behavioral problems, the parents are the main recipient of the therapy and thus become co-agents of changing their child’s behavior.

A total of 35 modules are included in the treatment manual. These modules are either *problem-specific or generic*.

For *anxiety* the *problem-specific modules* are:

©2020 Jeppesen P et al. *JAMA Psychiatry*.

1. Psychoeducation: awareness and scaling of emotions, introduction of the cognitive circle model.
2. Cognitive flexibility and restructuring: “detective thinking,” probability of the feared event and/or consequences, substituting negative thoughts with helpful thoughts, coping with worry.
3. Exposures: build a hierarchy of feared situations and plan rewards at each step, plan exposures and prevent avoidance, interceptive and in vivo exposures, work with identification and regulation of emotions.

For *depressive* symptoms the *problem-specific modules* are:

1. Psychoeducation: awareness and scaling of emotions, introduction to the cognitive circle model.
2. Behavioral activation: connect feelings and activities, register nourishing and demanding activities, schedule pleasant and nourishing activities, work with thought traps.
3. Cognitive flexibility and restructuring: “detective thinking,” ambiguous situations, substituting negative thoughts with helpful thoughts, how to stop ruminating.
4. Relaxation techniques.

For parent training targeting child *behavioral problems*, the *problem-specific modules* are:

1. Supporting positive behaviors:
    a. how to set clear directions
    b. house rules
    c. use praise, encouragement and incentive systems for contingency management.
2. Setting limits for the child:
    a. set healthy limits
    b. be involved and monitor child.
3. Positive family relationships:
    a. positive communication.
4. Supporting the parents:
    a. boost parents’ energy.

©2020 Jeppesen P et al. *JAMA Psychiatry*.

For training of children with *behavioral problems* (often combined with parent training) the *problem-specific modules* are:

1. The child's self-image
2. Recognition and regulation of emotions: scaling of emotions, body signals, controlling anger, progressive relaxation.

The *generic modules* within the MMM manual include (1) CBT techniques which are included in all treatment programs: case formulation at the beginning of treatment and a relapse prevention plan at the end of treatment, and (2) modules targeting possible underlying dysfunctions common to both anxiety, depression, and behavioral problems. Some of the generic modules are thus included in the prototypical treatment while others are optional. The *generic modules* include:

1. Case formulation and setting goals for the treatment
2. Social skills and social cognition (optional)
3. Problem-solving skills (optional)
4. Trauma narrative and cognitive processing of the traumatic event(s), (optional)
5. Motivation (optional)
6. Suicidal risk assessment (optional)
7. Review of acquired skills: how to maintain progress (relapse prevention plan) and set new goals.

Individually Adapted Transdiagnostic Treatment in MMM

The MMM manual was developed for help-seeking youth with emotional and behavioral problems above the 90-percentile in the general population of youth; and still below the threshold for referral to specialized treatment in the child and adolescent mental health services in Denmark. We aimed to address a population with unmet needs for clinical care in whom indicated prevention would be possible and relevant. Based on our experience with the visitation processes in the MMM feasibility trial, we expected most of the help-seeking youth to present with multiple problems. The transdiagnostic modular approach provides the therapist with a range of options for tailoring the therapy to current needs by the flexible dosing and sequencing of generic and problem-specific modules.

©2020 Jeppesen P et al. *JAMA Psychiatry*.

The MMM manual allows for two levels of individualized transdiagnostic adaptations: a) the therapist may continue with the same main course and use other generic or specific modules as extra modules if needed to address multiple problems while maintaining the original focus, or b) the therapist may shift from one main treatment program to another, if the understanding of the core psychopathology (i.e. the Top-problem) has changed. According to the MMM manual, a shift in the main program should always be approved by the supervisor, and the decision should be based on best practice for assessing the psychopathology. This way, the MMM manual allows for two levels of individualized transdiagnostic adaptations. There is a trade-off between the doses of treatment for one single problem versus the diluted doses of treatments for each of multiple problems. The MMM manual encourages the therapist to use minor transdiagnostic adaptations, and to refrain from a shift in the main program, unless the nature of the primary problem has clearly changed.

eMethods 4. Management as Usual (MAU)

To reduce the risk of attrition from the MAU group, the MAU treatment was enhanced compared to the regular MAU offered in the municipalities in the following ways:

The parents in the MAU group were offered two care-coordination sessions (at week 2 and week 17) by a psychologist (or other local professional). The care-coordinators were not trained in delivering the MMM treatment. The first meeting had to be held no later than 13 days after randomization (i.e., parallel with the first session of MMM therapy). The aim of the first meeting was to review the problems identified in the visitation process of the MMM study, to suggest relevant treatment offers in the municipality, and to help coordinate the interventions in the municipality. The second meeting had to be held no later than week 17 (i.e., parallel to the end of the MMM therapy). The aim of the second meeting was to evaluate the child's current problems and needs. The treatment offers in MAU varied considerably from no intervention to counselling, talk therapy, pedagogical advice, network meetings, and/or individual support in the school setting. Some children were offered CBT interventions, but access to manualized psychological treatment in the municipality was limited.

©2020 Jeppesen P et al. *JAMA Psychiatry*.

eFigure 1. The Prototypical Program for Anxiety in MMM

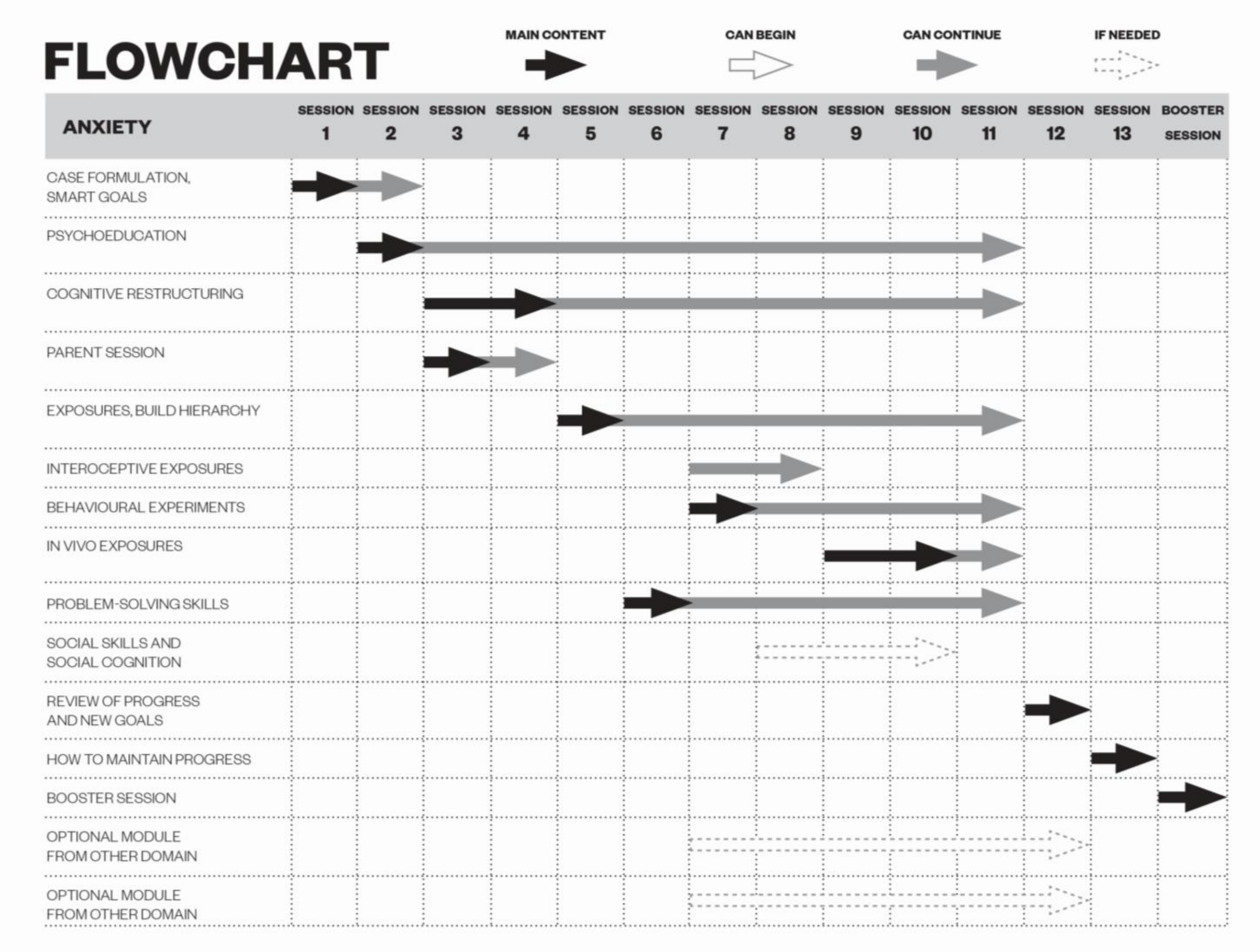

eFigure 1. Illustration of the prototypical sequencing and dosing of modules for anxiety.

©2020 Jeppesen P et al. *JAMA Psychiatry*.

eFigure 2. The Prototypical Program for Depressive Symptoms in MMM

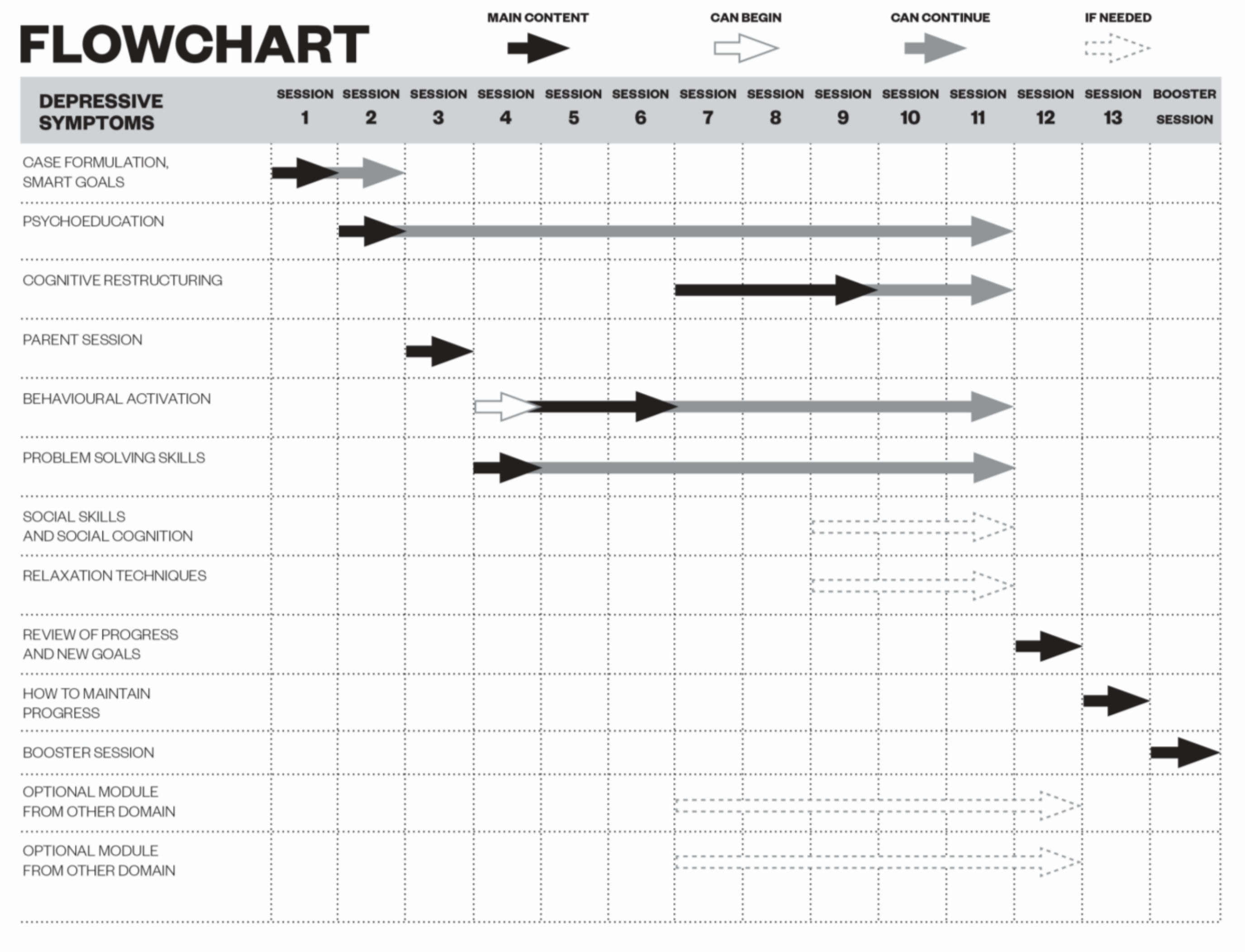

eFigure 2. Illustration of the prototypical sequencing and dosing of modules for depressive symptoms.

©2020 Jeppesen P et al. *JAMA Psychiatry*.

eFigure 3. The Prototypical Program for Parent Training in MMM

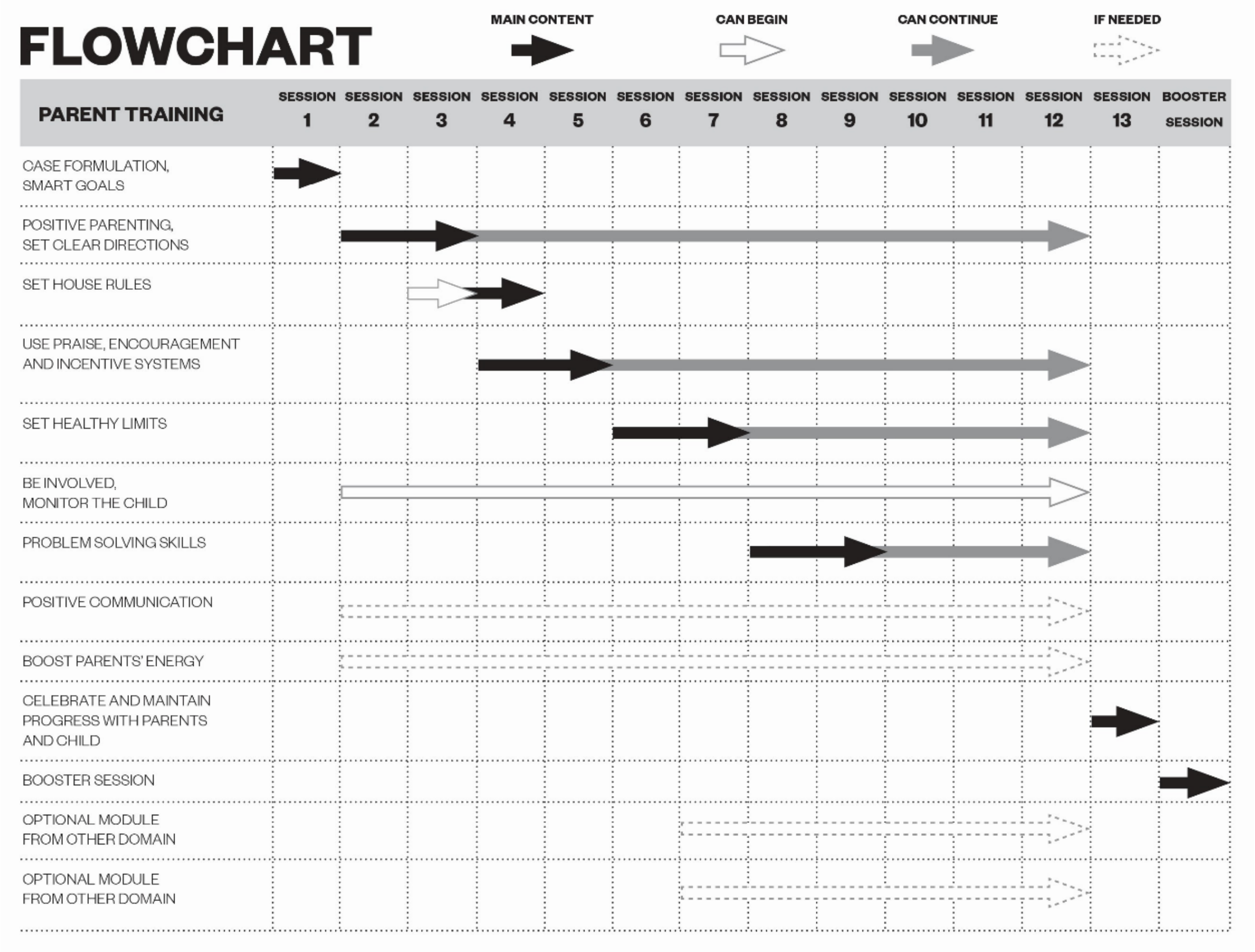

eFigure 3. Illustration of the prototypical sequencing and dosing of modules in the parent training targeting child behavioral problems.

©2020 Jeppesen P et al. *JAMA Psychiatry*.

eFigure 4. Scatterplot of the Correlation Between the Number of Sessions and the Primary Outcome Measure at 18 weeks, in the MMM Intervention Group (Observed Values)*

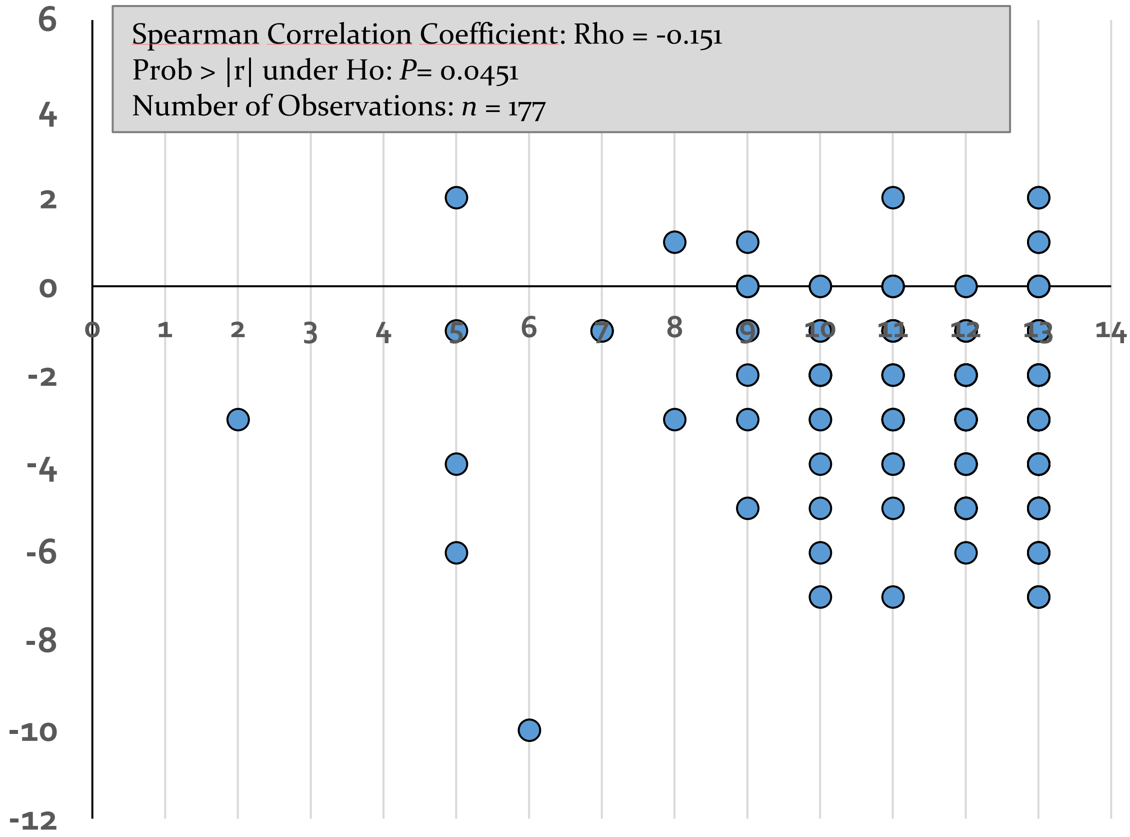

*Data at the x-axis are the number of therapy sessions (excluding booster sessions). Data at the Y-axis are the change in the parent-reported SDQ-Impact scores at 18 weeks (week 18 - week 0, range 0-10).

©2020 Jeppesen P et al. *JAMA Psychiatry*.

eTable 1. Potential Harms and Negative Outcomes at 18 Weeks and at 26 Weeks (the ITT Population)*

	n	MMM	n	MAU	Risk Ratio	P value
Composite score of suicidality and/or negative cognition (self-reported)						
Week 18	151	20 (13%)	121	22 (18%)	0.73 (0.42 to 1.27)	0.26
Week 26	150	7 (5%)	120	20 (17%)	0.28 (0.12 to 0.64)	0.003
Composite score of poor quality of family relationships, free time and/or friendships (self-reported)						
Week 18	173	20 (12%)	142	12 (8%)	1.37 (0.69 to 2.70)	0.37
Week 26	173	17 (10%)	140	14 (10%)	0.98 (0.50 to 1.92)	0.96
Referral to CAMHS† (parent-reported)						
From entry to week 18	176	7 (4%)	161	12 (7%)	0.53 (0.22 to 1.32)	0.17
From entry to week 26	178	10 (6%)	155	12 (8%)	0.73 (0.32 to 1.63)	0.44

*Data is presented as no (%)
† CAMHS, child and adolescent mental health services

Values are counts and percentages; no imputation used for missing data. Estimates for differences between groups are unadjusted risk ratios with corresponding 95% confidence intervals based on data as observed.

©2020 Jeppesen P et al. *JAMA Psychiatry*.

eTable 2. Other Exploratory Outcomes: Change from Baseline at 18 Weeks and 26 Weeks (the ITT Population)*

	n	MMM (18wks)	n	MAU (18wks)	Difference (18wks)	n	MMM (26wks)	n	MAU (26wks)	Difference (26wks)
**Teacher reports**										
Impact score (SDQ-impact*, teacher-reported)	143	–0.48 (1.29)	139	0.12 (1.35)	–0.60 (–0.91 to –0.29)	124	–0.72 (1.58)	127	0.01 (1.33)	–0.73 (–1.09 to –0.36)
Emotional and behavioral problems (SDQ-total-difficulties*, teacher-reported)	143	–1.55 (5.12)	139	–0.81 (4.44)	–0.74 (–1.86 to 0.38)	124	–2.00 (5.45)	127	–0.78 (4.85)	–1.22 (–2.50 to 0.06)
**Remaining self-reports**										
Impact score (SDQ-impact†, self-reported)	77	–1.06 (2.02)	62	–0.69 (2.12)	–0.37 (–1.07 to 0.32)	75	–1.43 (2.34)	60	–0.72 (2.37)	–0.71 (–1.52 to 0.10)
Emotional and behavioral problems (SDQ-total-difficulties†, self-reported)	77	–2.30 (4.59)	62	–1.23 (5.56)	–1.07 (–2.78 to 0.63)	75	–3.65 (5.43)	60	–1.58 (6.48)	–2.07 (–4.10 to –0.04)
Anxiety (SCAS‡, self-reported)	151	–6.13 (11.97)	123	0.79 (14.79)	–6.91 (–10.17 to –3.66)	150	–8.89 (12.45)	120	–1.62 (15.68)	–7.27 (–10.73 to –3.81)
Depressive symptoms (MFQ§, self-reported)	151	–4.21 (10.95)	121	–1.74 (9.71)	–2.47 (–4.97 to 0.03)	150	–7.52 (12.00)	118	–2.92 (11.34)	–4.60 (–7.44 to –1.77)
Top-problem score (self-reported)	174	–3.18 (2.62)	146	–1.31 (2.57)	–1.87 (–2.44 to –1.30)	173	–3.51 (2.67)	145	–1.52 (2.72)	–1.99 (–2.59 to –1.40)
Autonomy & Parents (KIDSCREEN‖, self-reported)	172	0.75 (11.07)	142	1.72 (10.48)	–0.97 (–3.38 to 1.44)	173	3.56 (11.07)	140	1.59 (9.63)	1.97 (–0.37 to 4.31)
Peers & Social Support (KIDSCREEN‖, self-reported)	173	1.22 (11.55)	141	1.45 (12.62)	–0.23 (–2.92 to 2.45)	173	2.06 (12.67)	139	0.75 (13.37)	1.31 (–1.60 to 4.22)
School Environment (KIDSCREEN‖, self-reported)	173	2.14 (9.65)	142	0.96 (11.19)	1.19 (–1.13 to 3.50)	173	3.18 (10.13)	140	1.32 (11.51)	1.86 (–0.55 to 4.27)
**Remaining parent reports**										
Physical Well-Being (KIDSCREEN‖, parent-reported)	176	3.16 (8.69)	165	2.53 (9.04)	0.63 (–1.26 to 2.52)	178	5.11 (9.04)	155	3.95 (9.33)	1.17 (–0.82 to 3.15)
Psychological Well-Being (KIDSCREEN‖, parent-reported)	176	5.94 (9.76)	165	2.57 (8.84)	3.38 (1.39 to 5.36)	178	7.54 (9.75)	155	4.61 (10.64)	2.93 (0.73 to 5.13)

©2020 Jeppesen P et al. *JAMA Psychiatry*.

Autonomy & Parents (KIDSCREEN‖, parent-reported)	175	1.52 (7.38)	164	0.57 (8.88)	0.95 (–0.80 to 2.70)	178	3.72 (9.07)	154	2.71 (8.72)	1.01 (–0.92 to 2.94)
Peers & Social Support (KIDSCREEN‖, parent-reported	176	2.71 (11.95)	164	1.75 (11.18)	0.96 (–1.51 to 3.43)	178	3.66 (13.03)	155	2.80 (12.89)	0.86 (–1.94 to 3.66)
School Environment (KIDSCREEN‖, parent-reported)	175	3.11 (9.77)	165	1.84 (9.79)	1.28 (–0.81 to 3.36)	178	3.63 (10.60)	153	2.74 (8.69)	0.88 (–1.20 to 2.97)
Parental Stress in role functioning (PSS ¶)	176	–1.05 (5.24)	165	–0.80 (5.55)	–0.25 (–1.40 to 0.90)	178	–1.88 (6.31)	155	–0.72 (5.79)	–1.15 (–2.47 to 0.16)
**Level of satisfaction**										
Experience of service (ESQ**, parent-reported)	176	18.0 (16.0, 20.0)	162	7.0 (4.0, 12.0)	11	178	18.5 (15.0, 20.0)	155	8.0 (4.0, 14.0)	10.5
Experience of Service (ESQ**, self-reported)	173	12.0 (9.0, 13.0)	142	6.0 (2.0, 9.0)	6	173	12.0 (9.0, 14.0)	140	7.0 (2.5, 10.0)	5

* The Strengths and Difficulties Questionnaire, teacher-report. SDQ-impact scale (0-6). SDQ-total-difficulties scale (0-40).
† The Strengths and Difficulties Questionnaire, self-report. SDQ-impact scale (0-10). SDQ-total-difficulties scale (0-40). The SDQ self-report was not administered to children below the age of 11 years.
‡ The Spence Children's Anxiety Scale (0-114). The SCAS self-report was not administered to children below the age of 8 years.
§ The Mood and Feelings Questionnaire (0-66). The MFQ self-report was not administered to children below the age of 8 years.
‖ A Health-Related Quality of Life (HRQOL) with five dimensions, of which we use the scales: Physical Well-Being, Psychological Well-Being (T-values [SD]).
¶ Parental Stress Scale
** Experience of Service Questionnaire. ESQ parent-reported and ESQ self-reported are values at follow-up since no baseline values exist for these outcomes. These are reported as medians interquartile range (IQR) and difference in medians.

Descriptive statistical estimates are mean changes (SD) and differences in mean changes (95%CI) at 18 weeks and 26 weeks follow-up. No imputation used for missing data.

©2020 Jeppesen P et al. *JAMA Psychiatry*.

eTable 3. Change From Baseline in Primary and Secondary Outcomes at 18 Weeks in the ITT Population: Missing Data Handled Using Multiple Imputation*

	n	MMM	n	MAU	Difference	P value
**Primary outcome measure**						
Impact score (SDQ-impact†, parent-reported)	197	–2.34 (0.15)	199	–1.21 (0.16)	–1.13 (–1.53 to –0.73)	<0.001
**Key secondary outcome measures**						
Anxiety (SCAS‡, parent-reported)	197	–6.79 (0.98)	199	–1.55 (1.01)	–5.24 (–7.71 to –2.77)	<0.001
Depressive symptoms (MFQ§, parent-reported)	197	–6.15 (0.68)	199	–2.99 (0.71)	–3.16 (–4.94 to –1.38)	<0.001
Level of daily functioning of child (WFIRS‖, parent-reported)	197	–7.64 (0.91)	199	–3.03 (0.96)	–4.61 (–6.98 to –2.24)	<0.001
School attendance¶ (parent-reported)	197	0.04 (0.01)	199	0.00 (0.02)	0.04 (0.00 to 0.07)	0.05
Top-problem score (parent-reported)	197	–3.02 (0.18)	199	–1.29 (0.18)	–1.73 (–2.19 to –1.26)	<0.001
KIDSCREEN-27 (self-reported) **						
Physical well-being	197	3.52 (0.79)	199	2.65 (0.81)	0.87 (–1.19 to 2.93)	0.41
Psychological well-being	197	3.17 (0.69)	199	1.06 (0.75)	2.11 (0.25 to 3.97)	0.03
Behavioral problems (ECBI††, parent-reported)						
Intensity score	197	–14.20 (1.45)	199	–6.90 (1.48)	–7.30 (–10.99 to –3.61)	<0.001
Problem score	197	–3.77 (0.40)	199	–2.42 (0.41)	–1.35 (–2.37 to –0.33)	0.010
Emotional & behavioral problems (SDQ-total-difficulties†, parent-reported)	197	–4.17 (0.35)	199	–2.02 (0.37)	–2.15 (–3.07 to –1.23)	<0.001
**Responder indices**						
Parent-reported SDQ-impact score ≥ 1 point reduction from baseline, no. (%)	197	160 (81%)	199	111 (56%)	3.49 (2.14 to 5.71)	<0.001
Parent-reported SDQ-scores below inclusion cut-off‡‡, no. (%)	197	108 (55%)	199	68 (34%)	2.49 (1.60 to 3.87)	<0.001

* Data are presented as mean (SD) unless otherwise stated.
† The Strengths and Difficulties Questionnaire. SDQ-impact scale (0-10). SDQ-total-difficulties scale (0-40).
‡ The Spence Children's Anxiety Scale (0-114).
§ The Mood and Feelings Questionnaire (0-68).
‖ The Weiss Functional Impairment Rating Scale (0-150).
¶ Percentage of school days in the last 4 weeks (0–100%).
** A Health-Related Quality of Life (HRQOL) with five dimensions, of which we use the scales: Physical Well-Being, Psychological Well-Being (T-values [SD]).
†† The Eyberg Child Behavior Inventory. Intensity score (36-252). Problem score (0-36).
‡‡ Inclusion cut-off: SDQ Total Difficulties score of at least 14, Emotional Problems score of at least 5, and/or Conduct Problems score of at least 3, combined with an SDQ Impact score of at least 1.

©2020 Jeppesen P et al. *JAMA Psychiatry*.

Missing data were replaced using multiple imputation. Multiple imputations were performed separately for each group using chained equations with predictive mean matching for continuous outcomes and logistic regression imputation models for binary outcomes. Variables in the imputation model included all covariates in the primary analysis model. A total of 100 complete datasets were generated, with treatment effect estimates combined across datasets using Rubin's rules[62].

Group-wise estimates for continuous outcomes are Least-Squares Means (SE), and dichotomous outcomes are mean numbers (%) at 18 weeks follow-up based on the 100 complete datasets. Estimates for difference between groups are based on Least-Squares Means with corresponding 95% confidence intervals (derived from linear models) for continuous outcomes. For dichotomous outcomes, estimates are odds ratios with corresponding 95% confidence intervals from logistic regression.

©2020 Jeppesen P et al. *JAMA Psychiatry*.

eTable 4. Exploratory Outcomes: Change from Baseline at 26 Weeks in The ITT Population: Missing Data Handled Using Multiple Imputation*

	n	MMM	n	MAU	Difference	P value
**Primary outcome measure**						
Impact score (SDQ-impact†, parent-reported)	197	–2.54 (1.63)	199	–1.43 (2.10)	–1.10 (–1.61 to –0.60)	<0.001
**Key secondary outcome measures**						
Anxiety (SCAS‡, parent-reported)	197	–8.04 (4.89)	199	–1.49 (8.03)	–6.55 (–9.36 to –3.73)	<0.001
Depressive symptoms (MFQ§, parent-reported)	197	–7.90 (3.80)	199	–3.85 (6.08)	–4.06 (–6.15 to –1.97)	<0.001
Level of daily functioning of child (WFIRS‖, parent-reported)	197	–9.20 (5.71)	199	–4.25 (7.49)	–4.94 (–7.92 to –1.97)	0.001
School attendance¶ (parent-reported)	197	0.06 (0.48)	199	0.02 (0.45)	0.05 (–0.00 to 0.10)	0.05
Top-problem score (parent-reported)	197	–3.26 (1.74)	199	–1.78 (1.83)	–1.48 (–2.00 to –0.96)	<0.001
KIDSCREEN-27 (self-reported) **						
Physical well-being	197	4.39 (4.69)	199	4.21 (6.43)	0.18 (–2.07 to 2.42)	0.88
Psychological well-being	197	4.76 (4.03)	199	3.10 (7.23)	1.66 (–0.49 to 3.82)	0.13
Behavioral problems (ECBI††, parent-reported)						
Intensity score	197	–18.13 (8.51)	199	–9.57 (11.83)	–8.56 (–13.45 to –3.68)	<0.001
Problem score	197	–4.97 (3.09)	199	–2.88 (3.20)	–2.09 (–3.36 to –0.82)	0.001
Emotional & behavioral problems (SDQ-total-difficulties†, parent-reported)	197	–5.19 (2.84)	199	–2.80 (3.30)	–2.39 (–3.50 to –1.29)	<0.001
**Responder indices**						
Parent-reported SDQ-impact score ≥ 1 point reduction from baseline, no. (%)	197	161 (82%)	199	122 (61%)	1.34 (1.15 to 1.56)	<0.001
Parent-reported SDQ-scores below inclusion cut-off‡‡, no. (%)	197	126 (64%)	199	79 (40%)	1.61 (1.25 to 2.06)	<0.001

* Data are presented as mean (SD) unless otherwise stated.
† The Strengths and Difficulties Questionnaire. SDQ-impact scale (0-10). SDQ-total-difficulties scale (0-40).
‡ The Spence Children's Anxiety Scale (0-114).
§ The Mood and Feelings Questionnaire (0-68).
‖ The Weiss Functional Impairment Rating Scale (0-150).
¶ Percentage of school days in the last 4 weeks (0–100%).
** A Health-Related Quality of Life (HRQOL) with five dimensions, of which we use the scales: Physical Well-Being, Psychological Well-Being (T-values [SD]).
†† The Eyberg Child Behavior Inventory. Intensity score (36-252). Problem score (0-36).

©2020 Jeppesen P et al. *JAMA Psychiatry*.

‡‡ Inclusion cut-off: SDQ Total Difficulties score of at least 14, Emotional Problems score of at least 5, and/or Conduct Problems score of at least 3, combined with an SDQ Impact score of at least 1.

Missing data were replaced using multiple imputation. Multiple imputations were performed separately for each group using chained equations with predictive mean matching for continuous outcomes and logistic regression imputation models for binary outcomes. Variables in the imputation model included all covariates in the primary analysis model. A total of 100 complete datasets were generated, with treatment effect estimates combined across datasets using Rubin's rules[62].

Group-wise estimates for continuous outcomes are mean changes (SDs), and dichotomous outcomes are mean numbers (%) at 26 weeks follow-up based on the 100 complete datasets. Estimates for difference between groups are unadjusted differences in mean changes with corresponding 95% confidence intervals for continuous outcomes; for dichotomous outcomes, differences are estimated as unadjusted risk ratios with corresponding 95% confidence intervals.

©2020 Jeppesen P et al. *JAMA Psychiatry*.

eTable 5. Change From Baseline in Primary and Secondary Outcomes at 18 Weeks: Per Protocol Population*

	n	MMM	n	MAU	Difference	P value
**Primary Outcome Measure**						
Impact score (SDQ-impact†, parent-reported)	164	–2.35 (0.13)	131	–1.21 (0.15)	–1.14 (–1.52 to –0.77)	<0.001
**Key Secondary Outcome Measures**						
Anxiety (SCAS‡, parent-reported)	164	–6.63 (0.73)	131	–1.60 (0.80)	–5.03 (–7.06 to –2.99)	<0.001
Depressive symptoms (MFQ§, parent-reported)	164	–5.88 (0.52)	131	–2.58 (0.58)	–3.30 (–4.77 to –1.83)	<0.001
Level of daily functioning of child (WFIRS‖, parent-reported)	164	–7.69 (0.69)	131	–2.99 (0.77)	–4.70 (–6.64 to –2.75)	<0.001
School attendance¶ (parent-reported)	164	0.03 (0.01)	131	0.00 (0.01)	0.03 (0.01 to 0.06)	0.01
Top-problem score (parent-reported)	164	–3.09 (0.14)	131	–1.28 (0.16)	–1.82 (–2.22 to –1.41)	<0.001
KIDSCREEN-27 (self-reported) **						
Physical well-being	164	3.04 (0.58)	131	2.31 (0.64)	0.73 (–0.91 to 2.36)	0.38
Psychological well-being	164	2.68 (0.53)	131	0.80 (0.59)	1.88 (0.39 to 3.37)	0.01
Behavioral problems (ECBI††, parent-reported)						
Intensity score	164	–14.42 (1.06)	131	–7.35 (1.17)	–7.07 (–10.05 to –4.08)	<0.001
Problem score	164	–3.81 (0.30)	131	–2.45 (0.33)	–1.36 (–2.20 to –0.52)	0.002
Emotional & behavioral problems (SDQ-total-difficulties†, parent-reported)	164	–4.13 (0.26)	131	–1.89 (0.29)	–2.24 (–2.97 to –1.50)	<0.001
**Responder indices**						
Parent-reported SDQ-impact score ≥ 1 point reduction from baseline, no. (%)	164	134 (82%)	131	73 (56%)	3.64 (2.14 to 6.19)	<0.001
Parent-reported SDQ-scores below inclusion cut-off‡‡, no. (%)	164	90 (55%)	131	43 (33%)	2.54 (1.57 to 4.12)	<0.001

* Only participants with data both at baseline and at week 18 for all primary and key secondary outcome measures are included in the analysis. Data are presented as mean (SD) unless otherwise stated.
† The Strengths and Difficulties Questionnaire. SDQ-impact scale (0-10). SDQ-total-difficulties scale (0-40).
‡ The Spence Children's Anxiety Scale (0-114).
§ The Mood and Feelings Questionnaire (0-68).
‖ The Weiss Functional Impairment Rating Scale (0-150).
¶ Percentage of school days in the last 4 weeks (0–100%).
** A Health-Related Quality of Life (HRQOL) with five dimensions, of which we use the scales: Physical Well-Being, Psychological Well-Being (T-values [SD]).
†† The Eyberg Child Behavior Inventory. Intensity score (36-252). Problem score (0-36).
‡‡ Inclusion cut-off: SDQ Total Difficulties score of at least 14, Emotional Problems score of at least 5, and/or Conduct Problems score of at least 3, combined with an SDQ Impact score of at least 1.

©2020 Jeppesen P et al. *JAMA Psychiatry*.

Estimates for difference between groups are based on Least-Squares Means with corresponding 95% confidence intervals (derived from linear mixed models with repeated measures) for continuous outcomes. For dichotomous outcomes, estimates are odds ratios with corresponding 95% confidence intervals from logistic regression with missing data conservatively assumed to be non-responders. Numbers indicate the number of patients from each group included in the models. Group-wise estimates for continuous outcomes are Least-Squares Means (SE), and dichotomous outcomes are n (%) at 18 weeks follow-up.

©2020 Jeppesen P et al. *JAMA Psychiatry*.

eTable 6. Change From Baseline in Primary and Secondary Outcomes at 18 Weeks: Before Trial Registration*

	n	MMM	n	MAU	Difference	P value
**Primary Outcome Measure**						
Impact score (SDQ-impact†, parent-reported)	119	–2.39 (0.17)	124	–1.40 (0.16)	–0.99 (–1.44 to –0.54)	<0.001
**Key Secondary Outcome Measures**						
Anxiety (SCAS‡, parent-reported)	119	–7.61 (0.92)	125	–2.45 (0.87)	–5.15 (–7.55 to –2.76)	<0.001
Depressive symptoms (MFQ§, parent-reported)	119	–7.29 (0.66)	125	–3.23 (0.63)	–4.06 (–5.78 to –2.34)	<0.001
Level of daily functioning of child (WFIRS‖, parent-reported)	119	–9.08 (0.77)	125	–3.57 (0.75)	–5.51 (–7.55 to –3.47)	<0.001
School attendance¶ (parent-reported)	119	0.04 (0.01)	125	0.00 (0.01)	0.04 (0.01 to 0.07)	0.02
Top-problem score (parent-reported)	119	–3.29 (0.18)	125	–1.37 (0.18)	–1.93 (–2.41 to –1.45)	<0.001
KIDSCREEN-27 (self-reported) **						
Physical well-being	119	4.12 (0.69)	125	3.54 (0.71)	0.58 (–1.29 to 2.45)	0.54
Psychological well-being	119	4.31 (0.63)	125	2.79 (0.65)	1.53 (–0.19 to 3.25)	0.08
Behavioral problems (ECBI††, parent-reported)						
Intensity score	119	–14.31 (1.28)	125	–6.88 (1.23)	–7.43 (–10.78 to –4.08)	<0.001
Problem score	119	–3.93 (0.37)	125	–2.24 (0.35)	–1.68 (–2.65 to –0.72)	<0.001
Emotional & behavioral problems (SDQ-total-difficulties†, parent-reported)	119	–4.71 (0.32)	124	–2.64 (0.31)	–2.08 (–2.92 to –1.24)	<0.001
**Responder indices**						
Parent-reported SDQ-impact score ≥ 1 point reduction from baseline, no. (%)	119	84 (71%)	125	65 (52%)	2.21 (1.30 to 3.76)	0.003
Parent-reported SDQ-scores below inclusion cut-off‡‡, no. (%)	119	61 (51%)	125	40 (32%)	2.30 (1.35 to 3.92)	0.002

* The analysis population consist of the participants enrolled before the final trial registration was approved (and publicly available) in ClinicalTrials.gov. Data are presented as mean (SD) unless otherwise stated.
† The Strengths and Difficulties Questionnaire. SDQ-impact scale (0-10). SDQ-total-difficulties scale (0-40).
‡ The Spence Children's Anxiety Scale (0-114).
§ The Mood and Feelings Questionnaire (0-68).
‖ The Weiss Functional Impairment Rating Scale (0-150).
¶ Percentage of school days in the last 4 weeks (0–100%).
** A Health-Related Quality of Life (HRQOL) with five dimensions, of which we use the scales: Physical Well-Being, Psychological Well-Being (T-values [SD]).
†† The Eyberg Child Behavior Inventory. Intensity score (36-252). Problem score (0-36).
‡‡ Inclusion cut-off: SDQ Total Difficulties score of at least 14, Emotional Problems score of at least 5, and/or Conduct Problems score of at least 3, combined with an SDQ Impact score of at least 1.

©2020 Jeppesen P et al. *JAMA Psychiatry*.

Estimates for difference between groups are based on Least-Squares Means with corresponding 95% confidence intervals (derived from linear mixed models with repeated measures) for continuous outcomes. For dichotomous outcomes, estimates are odds ratios with corresponding 95% confidence intervals from logistic regression with missing data conservatively assumed to be non-responders. Numbers indicate the number of patients from each group included in the models. Group-wise estimates for continuous outcomes are Least-Squares Means (SE), and dichotomous outcomes are n (%) at 18 weeks follow-up.

©2020 Jeppesen P et al. *JAMA Psychiatry*.

eTable 7. Change From Baseline in Primary and Secondary Outcomes at 18 Weeks: After Trial Registration*

	n	MMM	n	MAU	Difference	P value
**Primary Outcome Measure**						
Impact score (SDQ-impact†, parent-reported)	78	–2.24 (0.20)	74	–0.95 (0.23)	–1.29 (–1.87 to –0.71)	<0.001
**Key Secondary Outcome Measures**						
Anxiety (SCAS‡, parent-reported)	78	–4.45 (0.91)	74	0.87 (1.04)	–5.32 (–7.92 to –2.73)	<0.001
Depressive symptoms (MFQ§, parent-reported)	78	–3.78 (0.67)	74	–1.72 (0.77)	–2.06 (–3.98 to –0.14)	0.04
Level of daily functioning of child (WFIRS‖, parent-reported)	78	–5.43 (1.02)	74	–1.32 (1.16)	–4.11 (–7.03 to –1.19)	0.006
School attendance¶ (parent-reported)	78	0.03 (0.01)	74	0.01 (0.02)	0.02 (-0.02 to 0.06)	0.29
Top-problem score (parent-reported)	78	–2.80 (0.21)	74	–1.42 (0.24)	–1.38 (–1.98 to –0.78)	<0.001
KIDSCREEN-27 (self-reported) **						
Physical well-being	78	1.78 (0.88)	74	1.01 (1.04)	0.77 (–1.82 to 3.37)	0.56
Psychological well-being	78	0.72 (0.76)	74	–1.92 (0.90)	2.64 (0.40 to 4.88)	0.02
Behavioral problems (ECBI††, parent-reported)						
Intensity score	78	–12.73 (1.53)	74	–5.55 (1.76)	–7.18 (–11.57 to –2.79)	0.002
Problem score	78	–3.22 (0.40)	74	–2.41 (0.46)	–0.81 (–1.96 to 0.33)	0.16
Emotional & behavioral problems (SDQ-total-difficulties†, parent-reported)	78	–3.17 (0.36)	74	–0.64 (0.42)	–2.53 (–3.58 to –1.49)	<0.001
**Responder indices**						
Parent-reported SDQ-impact score ≥ 1 point reduction from baseline, no. (%)	78	60 (77%)	74	28 (38%)	6.06 (2.90 to 12.67)	<0.001
Parent-reported SDQ-scores below inclusion cut-off‡‡, no. (%)	78	37 (47%)	74	16 (22%)	3.53 (1.71 to 7.29)	<0.001

* The analysis population consist of the participants enrolled after the final trial registration was approved (and publicly available) in ClinicalTrials.gov. Data are presented as mean (SD) unless otherwise stated.
† The Strengths and Difficulties Questionnaire. SDQ-impact scale (0-10). SDQ-total-difficulties scale (0-40).
‡ The Spence Children's Anxiety Scale (0-114).
§ The Mood and Feelings Questionnaire (0-68).
‖ The Weiss Functional Impairment Rating Scale (0-150).
¶ Percentage of school days in the last 4 weeks (0–100%).
** A Health-Related Quality of Life (HRQOL) with five dimensions, of which we use the scales: Physical Well-Being, Psychological Well-Being (T-values [SD]).
†† The Eyberg Child Behavior Inventory. Intensity score (36-252). Problem score (0-36).
‡‡ Inclusion cut-off: SDQ Total Difficulties score of at least 14, Emotional Problems score of at least 5, and/or Conduct Problems score of at least 3, combined with an SDQ Impact score of at least 1.

©2020 Jeppesen P et al. *JAMA Psychiatry*.

Estimates for difference between groups are based on Least-Squares Means with corresponding 95% confidence intervals (derived from linear mixed models with repeated measures) for continuous outcomes. For dichotomous outcomes, estimates are odds ratios with corresponding 95% confidence intervals from logistic regression with missing data conservatively assumed to be non-responders. Numbers indicate the number of patients from each group included in the models. Group-wise estimates for continuous outcomes are Least-Squares Means (SE), and dichotomous outcomes are n (%) at 18 weeks follow-up.

©2020 Jeppesen P et al. *JAMA Psychiatry*.

eTable 8. Correlation Matrix Exploring the Intercorrelations of the Primary and Key Secondary Outcomes (Change Scores) in the MMM Group*

	Impact score (SDQ-impact† , parent-reported)	Anxiety (SCAS‡ , parent-reported)	Depressive symptoms (MFQ§ , parent-reported)	Level of daily functioning (WFIRS‖, parent-reported	School attendance¶ (parent-reported	Top-problem score (parent-reported	KIDSCREEN-27** Physical well-being (self-reported)	KIDSCREEN-27**Psychological well-being, (self-reported)	Behavioral problems (ECBI† † , Intensity, parent-reported)	Behavioral problems (ECBI† † , Problems, parent-reported	Emotional and behavioral problems (SDQ-total-difficulties† , parent-reported)
Impact score (SDQ-impact†, parent-reported)	1.000										
P value											
n	177										
Anxiety (SCAS‡, parent-reported)	0.308	1.000									
P value	<0.001										
n	177	177									
Depressive symptoms (MFQ§, parent-reported)	0.355	0.534	1.000								
P value	<0.001	<0.001									
n	176	176	176								
Level of daily functioning (WFIRS‖, parent-reported)	0.339	0.475	0.585	1.000							
P value	<0.001	<0.001	<0.001								
n	176	176	176	176							
School attendance¶ (parent-reported)	-0.16	-0.106	-0.251	-0.149	1.000						
P value	0.037	0.168	0.001	0.052							
n	170	170	170	170	170						
Top-problem score (parent-reported)	0.281	0.515	0.462	0.449	-0.181	1.000					
P value	<0.001	<0.001	<0.001	<0.001	0.018						
n	177	177	176	176	170	177					
KIDSCREEN-27** Physical well-being (self-reported)	-0.08	-0.237	-0.284	-0.33	0.189	-0.259	1.000				
P value	0.301	0.002	<0.001	<0.001	0.015	0.001					
n	171	171	171	171	165	171	173				
KIDSCREEN-27**Psychological well-being, (self-reported)	-0.291	-0.283	-0.439	-0.34	0.186	-0.32	0.467	1.000			
P value	<0.001	<0.001	<0.001	<0.001	0.017	<0.001	<0.001				
n	170	170	170	170	164	170	172	172			
Behavioral problems (ECBI††, Intensity, parent-reported)	0.19	0.344	0.424	0.53	-0.052	0.341	-0.273	-0.206	1.000		
P value	0.011	<0.001	<0.001	<0.001	0.499	<0.001	<0.001	0.007			
n	176	176	176	176	170	176	171	170	176		
Behavioral problems (ECBI††, Problems, parent-reported)	0.167	0.316	0.39	0.476	-0.003	0.294	-0.246	-0.164	0.719	1.000	
P value	0.027	<0.001	<0.001	<0.001	0.972	<0.001	0.001	0.033	<0.001		
n	176	176	176	176	170	176	171	170	176	176	
Emotional and behavioral problems (SDQ-total-difficulties†, parent-reported)	0.371	0.405	0.394	0.511	-0.096	0.364	-0.3	-0.238	0.336	0.318	1.000
P value	<0.001	<0.001	<0.001	<0.001	0.214	<0.001	<0.001	0.002	<0.001	<0.001	

©2020 Jeppesen P et al. *JAMA Psychiatry*.

n	177	177	176	176	170	177	171	170	176	176	177

*Values are Spearman correlation coefficients, p-values and number of participants, respectively.

† The Strengths and Difficulties Questionnaire. SDQ-impact scale (0-10). SDQ-total-difficulties scale (0–40).

‡ The Spence Children's Anxiety Scale (0–114).

§ The Mood and Feelings Questionnaire (0–68).

‖ The Weiss Functional Impairment Rating Scale (0–150).

¶ Percentage of school days in the last 4 weeks (0–100%).

** Health-Related Quality of Life (HRQOL) with five dimensions, of which we used the Physical Well-Being and Psychological Well-Being scales (t-values [SD]).

†† The Eyberg Child Behavior Inventory. Intensity score (36–252). Problem score (0-36).

©2020 Jeppesen P et al. *JAMA Psychiatry*.

eTable 9. Correlation Matrix Exploring the Intercorrelations of the Primary and Key Secondary Outcomes (Change Scores) in the MAU Group*

	Impact score (SDQ-impact† , parent-reported)	Anxiety (SCAS‡ , parent-reported)	Depressive symptoms (MFQ§ , parent-reported)	Level of daily functioning (WFIRS‖, parent-reported	School attendance¶ (parent-reported	Top-problem score (parent-reported	KIDSCREEN-27** Physical well-being (self-reported)	KIDSCREEN-27**Psychological well-being, (self-reported)	Behavioral problems (ECBI† † , Intensity, parent-reported)	Behavioral problems (ECBI† † , Problems, parent-reported	Emotional and behavioral problems (SDQ-total-difficulties† , parent-reported)
Impact score (SDQ-impact†, parent-reported)	1.000										
P value											
n	166										
Anxiety (SCAS‡, parent-reported)	0.455	1.000									
P value	<0.001										
n	166	167									
Depressive symptoms (MFQ§, parent-reported)	0.419	0.581	1.000								
P value	<0.001	<0.001									
n	166	167	167								
Level of daily functioning (WFIRS‖, parent-reported)	0.507	0.526	0.604	1.000							
P value	<0.001	<0.001	<0.001								
n	161	162	162	162							
School attendance¶ (parent-reported)	-0.096	-0.239	-0.21	-0.282	1.000						
P value	0.236	0.003	0.009	<0.001							
n	153	153	153	153	153						
Top-problem score (parent-reported)	0.436	0.387	0.451	0.344	-0.12	1.000					
P value	<0.001	<0.001	<0.001	<0.001	0.139						
n	166	167	167	162	153	167					
KIDSCREEN-27** Physical well-being (self-reported)	-0.171	-0.177	-0.19	-0.168	0.184	-0.23	1.000				
P value	0.044	0.036	0.024	0.048	0.035	0.006					
n	139	140	140	140	131	140	142				
KIDSCREEN-27**Psychological well-being, (self-reported)	-0.261	-0.265	-0.367	-0.197	0.171	-0.299	0.498	1.000			
P value	0.002	0.002	<0.001	0.02	0.051	<0.001	<0.001				
n	139	140	140	140	131	140	142	142			
Behavioral problems (ECBI††, Intensity, parent-reported)	0.347	0.33	0.513	0.491	-0.18	0.293	-0.193	-0.164	1.000		
P value	<0.001	<0.001	<0.001	<0.001	0.026	<0.001	0.022	0.053			
n	164	165	165	162	153	165	140	140	165		
Behavioral problems (ECBI††, Problems, parent-reported)	0.301	0.28	0.354	0.448	-0.214	0.27	-0.29	-0.157	0.634	1.000	
P value	<0.001	<0.001	<0.001	<0.001	0.008	<0.001	0.001	0.063	<0.001		
n	164	165	165	162	153	165	140	140	165	165	
Emotional and behavioral problems (SDQ-total-difficulties†, parent-reported)	0.485	0.467	0.451	0.501	-0.171	0.387	-0.158	-0.293	0.494	0.451	1.000
P value	<0.001	<0.001	<0.001	<0.001	0.035	<0.001	0.063	<0.001	<0.001	<0.001	

©2020 Jeppesen P et al. *JAMA Psychiatry*.

n	166	166	166	161	153	166	139	139	164	164	166

*Values are Spearman correlation coefficients, p-values and number of participants, respectively.

† The Strengths and Difficulties Questionnaire. SDQ-impact scale (0-10). SDQ-total-difficulties scale (0–40).

‡ The Spence Children's Anxiety Scale (0–114).

§ The Mood and Feelings Questionnaire (0–68).

‖ The Weiss Functional Impairment Rating Scale (0–150).

¶ Percentage of school days in the last 4 weeks (0–100%).

** Health-Related Quality of Life (HRQOL) with five dimensions, of which we used the Physical Well-Being and Psychological Well-Being scales (t-values [SD]).

†† The Eyberg Child Behavior Inventory. Intensity score (36–252). Problem score (0-36).

Based on the conventional interpretation of correlations (0.0-0.39 are weak, 0.40-0.59 are moderate, 0.60-0.79 are strong, and 0.80-1 are very strong (BMJ Resources for readers at https://www.bmj.com/about-bmj/resources-readers/publications/statistics-square-one/11-correlation-and-regression), none of the correlations were "very strong". As expected, the change-scores for the two ECBI-intensity and ECBI-problem scores (both measuring behavioral problems) correlated strongly. The majority of the parent-reported change-scores showed weak correlations, and most noticeably, the change in parent-reported impact of problems (the primary outcome) was weakly correlated with each of the key secondary outcomes, except that in the MAU group, the change in parent-reported impact of problems correlated moderately with four key secondary outcomes measuring anxiety, depressive symptoms, daily functioning, and total symptoms.

©2020 Jeppesen P et al. *JAMA Psychiatry*.

eTable 10. Subgroup Analysis for the Observed Parent-Reported SDQ Impact Scores (n = 343)

	Subgroup	n	MMM	n	MAU	Difference	P value for interaction
**Age group**	6-10 y	97	1.64 (0.20)	90	2.61 (0.21)	-0.97 (-1.75 to -0.19)	0.404
	11-16 y	80	1.98 (0.22)	76	3.29 (0.23)	-1.31 (-2.17 to -0.46)	
**Region**	Holstebro, Helsingør	86	1.62 (0.21)	83	2.77 (0.22)	-1.15 (-1.98 to -0.33)	0.894
	Vordingborg, Næstved	91	2.02 (0.21)	83	3.12 (0.22)	-1.10 (-1.91 to -0.29)	

Subgroup estimates for difference between groups are based on Least-Squares Means with corresponding 95% confidence intervals (derived from an ANCOVA model with a main effect for group and covariate [subgroup] plus the interaction between group and covariate, while adjusting for the same design variables as the primary model). Numbers indicate the number of patients from each group included in the model. Group-wise estimates are Least-Squares Means (SE) at 18 weeks follow-up.

eReferences.

1. Chorpita BF, Daleiden EL, Weisz JR. Identifying and selecting the common elements of evidence based interventions: A distillation and matching model. *Ment Health Serv Res*. 2005;7(1):5-20. doi:10.1007/s11020-005-1962-6

2. Davis III TE, May A, Whiting SE. Evidence-based treatment of anxiety and phobia in children and adolescents: Current status and effects on the emotional response. *Clin Psychol Rev*. 2011;31(4):592-602. doi:10.1016/j.cpr.2011.01.001

3. Compton SN, March JS, Brent D, Albano AM, Weersing VR, Curry J. Cognitive-behavioral psychotherapy for anxiety and depressive disorders in children and adolescents: An evidence-based medicine review. *J Am Acad Child Adolesc Psychiatry*. 2004;43(8):930-959. doi:10.1097/01.chi.0000127589.57468.bf

4. Higa-McMillan CK, Francis SE, Rith-Najarian L, Chorpita BF. Evidence Base Update: 50 Years of Research on Treatment for Child and Adolescent Anxiety. *J Clin Child Adolesc Psychol*. 2016;45(2):91-113. doi:10.1080/15374416.2015.1046177

5. Reynolds S, Wilson C, Austin J, Hooper L. Effects of psychotherapy for anxiety in children and adolescents: A meta-analytic review. *Clin Psychol Rev*. 2012;32(4):251-262. doi:10.1016/j.cpr.2012.01.005

6. Mychailyszyn MP, Brodman DM, Read KL, Kendall PC. Cognitive-Behavioral School-Based Interventions for Anxious and Depressed Youth: A Meta-Analysis of Outcomes. *Clin Psychol Sci Pract*. 2012;19(2):129-153. doi:10.1111/j.1468-2850.2012.01279.x

7. James A, James G, Cowdrey F, Soler A, Choke A. Cognitive behavioural therapy for anxiety disorders in children and adolescents (Review). *Cochrane Database Syst Rev*. 2018;(2). doi:10.10.1002/14651858.CD004690.pub4.

8. Schwartz C, Barican J Lou, Yung D, Zheng Y, Waddell C. Six decades of preventing and treating childhood anxiety disorders: a systematic review and meta-analysis to inform policy and practice. *Evid Based Ment Heal*. 2019;22(3):103-110. doi:10.1136/ebmental-2019-300096

9. Zhou X, Zhang Y, Furukawa TA, et al. Different Types and Acceptability of Psychotherapies

for Acute Anxiety Disorders in Children and Adolescents: A Network Meta-analysis. *JAMA Psychiatry*. 2019;76(1):41-50. doi:10.1001/jamapsychiatry.2018.3070

10. Hudson JL, Keers R, Roberts S, et al. Clinical predictors of response to cognitive-behavioral therapy in pediatric anxiety disorders: The genes for treatment (GxT) study. *J Am Acad Child Adolesc Psychiatry*. 2015;54(6):454-463. doi:10.1016/j.jaac.2015.03.018

11. Ollendick TH, Jarrett MA, Grills-Taquechel AE, Hovey LD, Wolff JC. Comorbidity as a predictor and moderator of treatment outcome in youth with anxiety, affective, attention deficit/hyperactivity disorder, and oppositional/conduct disorders. *Clin Psychol Rev*. 2008;28(8):1447-1471. doi:10.1016/j.cpr.2008.09.003

12. Mahdi M, Jhawar S, Bennett SD, Shafran R. Cognitive behavioral therapy for childhood anxiety disorders: What happens to comorbid mood and behavioral disorders? A systematic review. *J Affect Disord*. 2019;251:141-148.

13. Lebowitz ER, Shic F, Campbell D, Macleod J, Silverman WK. Avoidance moderates the association between mothers' and children's fears: Findings from a novel motion-tracking behavioral assessment. *Depress Anxiety*. 2015;32(2):91-98. doi:10.1002/da.22333

14. Manassis K, Changgun Le T, Bennett K, et al. Types of parental involvement in CBT with anxious youth: A preliminary meta-analysis. *J Consult Clin Psychol*. 2014;82(6):1163-1172. doi:10.1037/a0036969

15. Sun M, Rith-Najarian LR, Williamson TJ, Chorpita BF. Treatment Features Associated with Youth Cognitive Behavioral Therapy Follow-Up Effects for Internalizing Disorders: A Meta-Analysis. *J Clin Child Adolesc Psychol*. 2019;48(sup1):S269-S283. doi:10.1080/15374416.2018.1443459

16. Gearing RE, Schwalbe CSJ, Lee RH, Hoagwood KE. The effectiveness of booster sessions in CBT treatment for child and adolescent mood and anxiety disorders. *Depress Anxiety*. 2013;30(9):800-808. doi:10.1002/da.22118

17. Kendall P, Hedtke K. *Cognitive-Behavioral Therapy for Anxious Children: Therapist Manual*. 3rd Editio. Child and Adolescent Anxiety Disorders Clinic Temple University; 2006.

18. Kendall P, Crawley S, Benjamin C, Mauro C. *The Brief Coping Cat: Therapist Manual for the*

8-Session Workbook.; 2013.

19. Rapee R, Lyneham H, Schniering C, Wuthrich V, Abbott M, Hudson J. *The Cool Kids: Child and Adolescent Anxiety Program*. Anxiety Research Unit Macquarie University; 2006.

20. Arendt K, Thastum M, Hougaard E. Efficacy of a Danish version of the Cool Kids program: A randomized wait-list controlled trial. *Acta Psychiatr Scand*. 2016;133(2):109-121. doi:10.1111/acps.12448

21. Arnberg A, Öst LG. CBT for Children with Depressive Symptoms: A Meta-Analysis. *Cogn Behav Ther*. 2014;43(4):275-288. doi:10.1080/16506073.2014.947316

22. Forti-Buratti MA, Saikia R, Wilkinson EL, Ramchandani PG. Psychological treatments for depression in pre-adolescent children (12 years and younger): systematic review and meta-analysis of randomised controlled trials. *Eur Child Adolesc Psychiatry*. 2016;25(10):1045-1054. doi:10.1007/s00787-016-0834-5

23. David-Ferdon C, Kaslow NJ. Evidence-Based Psychosocial Treatments for Child and Adolescent Depression. *J Clin Child Adolesc Psychol*. 2008;37(1):62-104. doi:10.1080/15374410701817865

24. Zhou X, Hetrick SE, Cuijpers P, et al. Comparative efficacy and acceptability of psychotherapies for depression in children and adolescents: A systematic review and network meta-analysis. *World Psychiatry*. 2015;14(2):207-222. doi:10.1002/wps.20217

25. Hetrick SE, Cox GR, Witt KG, Bir JJ, Merry SN. Cognitive behavioural therapy (CBT), third-wave CBT and interpersonal therapy (IPT) based interventions for preventing depression in children and adolescents. *Cochrane Database Syst Rev*. 2016;(8). doi:10.1002/14651858.CD003380.pub4

26. Eckshtain D, Kuppens S, Ugueto A, et al. Meta-Analysis: 13-Year Follow-up of Psychotherapy Effects on Youth Depression. *J Am Acad Child Adolesc Psychiatry*. Published online April 2019. doi:10.1016/j.jaac.2019.04.002

27. Stice E, Shaw H, Bohon C, Marti CN, Rohde P. A meta-analytic review of depression prevention programs for children and adolescents: Factors that predict magnitude of intervention effects. *J Consult Clin Psychol*. 2009;77(3):486-503. doi:10.1037/a0015168

©2020 Jeppesen P et al. *JAMA Psychiatry*.

28. Weisz JR, McCarty CA, Valeri SM. Effects of psychotherapy for depression in children and adolescents: A meta-analysis. *Psychol Bull*. 2006;132(1):132-149. doi:10.1037/0033-2909.132.1.132

29. Hetrick SE, Cox GR, Merry SN. Where to go from Here? An Exploratory Meta-Analysis of the Most Promising Approaches to Depression Prevention Programs for Children and Adolescents. *Int J Environ Res Public Health*. 2015;12(5):4758-4795. doi:10.3390/ijerph120504758

30. Cox GR, Callahan P, Churchill R, et al. Psychological therapies versus antidepressant medication, alone and in combination for depression in children and adolescents. *Cochrane Database Syst Rev*. 2014;2014(11. Art. No.: CD008324). doi:10.1002/14651858.CD008324.pub3

31. Caldwell DM, Davies SR, Hetrick SE, et al. School-based interventions to prevent anxiety and depression in children and young people: a systematic review and network meta-analysis. *The Lancet Psychiatry*. 2019;6(12):1011-1020. doi:10.1016/S2215-0366(19)30403-1

32. Hetrick SE, Cox GR, Fisher CA, et al. Back to basics: could behavioural therapy be a good treatment option for youth depression? A critical review. *Early Interv Psychiatry*. 2015;9(2):93-99. doi:10.1111/eip.12142

33. Tindall L, Mikocka-Walus A, McMillan D, Wright B, Hewitt C, Gascoyne S. Is behavioural activation effective in the treatment of depression in young people? A systematic review and meta-analysis. *Psychol Psychother Theory, Res Pract*. 2017;90(4):770-796. https://rmidt.tdnetdiscover.com/resolver/full?output=full&sid=OVID:medline&id=pmid:28299896&id=doi:10.1111%2Fpapt.12121&issn=1476-0835&isbn=&volume=90&issue=4&spage=770&pages=770-796&date=2017&title=Psychology+%26+Psychotherapy%3A+Theory%2C+Research+%26+P

34. Martin F, Oliver T. Behavioral activation for children and adolescents: a systematic review of progress and promise. *Eur Child Adolesc Psychiatry*. 2019;28(4):427-441.

35. Curry J, Wells K, Brent D, Clarke G, Rohde P, Albano A. *Treatment of Adolescents with Depression Study (TADS) Cognitive Behavior Therapy Manual: Introduction, Rationale, and*

©2020 Jeppesen P et al. *JAMA Psychiatry*.

Adolescent Sessions.; 2003.

36. Bearman S, Ugueto A, Alleyne A, Weisz J. Adapting CBT for depression to fit diverse youths and contexts: Applying the deployment-focused model of treatment development and testing. In: Weisz J, Kazdin A, eds. *Evidence-Based Psychotherapies for Children and Adolescents*. 2nd editio. Guilford; 2010:466-481. Accessed July 30, 2019. http://scholar.harvard.edu/jweisz/publications/adapting-cbt-depression-fit-diverse-youths-and-contexts-applying-deployment-focu

37. Brunwasser SM, Gillham JE, Kim ES. A Meta-Analytic Review of the Penn Resiliency Program's Effect on Depressive Symptoms. *J Consult Clin Psychol*. 2009;77(6):1042-1054. doi:10.1037/a0017671

38. Stark K, Streusand W, Krumholz L, Patel P. Cognitive-behavioral therapy for depression: The ACTION treatment program for girls. In: J. R. Weisz & A. E. Kazdin, ed. *Evidence-Based Psychotherapies for Children and Adolescents*. The Guilford Press; 2010:93-109. Accessed July 30, 2019. https://psycnet.apa.org/record/2010-09488-007

39. Loevaas MES, Sund AM, Lydersen S, et al. Does the Transdiagnostic EMOTION Intervention Improve Emotion Regulation Skills in Children? *J Child Fam Stud*. 2019;28(3):805-813. doi:10.1007/s10826-018-01324-1

40. Furlong M, McGilloway S, Bywater T, Hutchings J, Smith S, Donnelly M. Behavioural and cognitive-behavioural group-based parenting programmes for early-onset conduct problems in children aged 3 to 12 years (Review). *Cochrane Database Syst Rev*. 2012;2(2). doi:10.1002/14651858.CD008225.pub2.www.cochranelibrary.com

41. Mytton JA, DiGuiseppi C, Gough D, Taylor RS, Logan S. School-based secondary prevention programmes for preventing violence. *Cochrane Database Syst Rev*. 2006;2(2):814-891. doi:10.1002/14651858.CD004606.pub2

42. Stoltz S. *Stay Cool Kids ?! Effectiveness, Moderation and Mediation of a Preventive Intervention for Externalizing Behavior.* Utrecht University Repository; 2012.

43. Wilson SJ, Lipsey MW. School-Based Interventions for Aggressive and Disruptive Behavior. Update of a Meta-Analysis. *Am J Prev Med*. 2007;33(2 SUPPL.):130-143. doi:10.1016/j.amepre.2007.04.011

©2020 Jeppesen P et al. *JAMA Psychiatry*.

44. Stoltz S, Deković M, van Londen M, Orobio de Castro B, Prinzie P. What Works for Whom, How and under What Circumstances? Testing Moderated Mediation of Intervention Effects on Externalizing Behavior in Children. *Soc Dev*. 2013;22(2):406-425. doi:10.1111/sode.12017

45. Battagliese G, Caccetta M, Luppino OI, et al. Cognitive-behavioral therapy for externalizing disorders: A meta-analysis of treatment effectiveness. *Behav Res Ther*. 2015;75:60-71. doi:10.1016/j.brat.2015.10.008

46. Michelson D, Davenport C, Dretzke J, Barlow J, Day C. Do Evidence-Based Interventions Work When Tested in the "Real World?" A Systematic Review and Meta-analysis of Parent Management Training for the Treatment of Child Disruptive Behavior. *Clin Child Fam Psychol Rev*. 2013;16(1):18-34. doi:10.1007/s10567-013-0128-0

47. McCart MR, Priester PE, Davies WH, Azen R. Differential effectiveness of behavioral parent-training and cognitive-behavioral therapy for antisocial youth: A meta-analysis. *J Abnorm Child Psychol*. 2006;34(4):527-543. doi:10.1007/s10802-006-9031-1

48. Webster-Stratton C, Rinaldi J, Reid JM. Long-Term Outcomes of Incredible Years Parenting Program: Predictors of Adolescent Adjustment. *Child Adolesc Ment Health*. 2011;16(1):38-46. doi:10.1111/j.1475-3588.2010.00576.x

49. Forgatch MS, Patterson GR, Degarmo DS, Beldavs ZG. Testing the Oregon delinquency model with 9-year follow-up of the Oregon Divorce Study. *Dev Psychopathol*. 2009;21(2):637-660. doi:10.1017/S0954579409000340

50. Wilson P, Rush R, Hussey S, et al. How evidence-based is an "evidence-based parenting program"? A PRISMA systematic review and meta-analysis of Triple P. *BMC Med*. 2012;10(1):130. doi:10.1186/1741-7015-10-130

51. Kazdin A, Siegel TC, Bass D. Cognitive Problem-Solving Skills Training and Parent Management Training in the Treatment of Antisocial Behavior in Children. *J Consult Clin Psychol*. 1992;60(5):733-747.

52. Lochman JE, Wells KC, Qu L, Chen L. Three Year Follow-Up of Coping Power Intervention Effects: Evidence of Neighborhood Moderation? *Prev Sci*. 2013;14(4):364-376. doi:10.1007/s11121-012-0295-0

©2020 Jeppesen P et al. *JAMA Psychiatry*.

53. Lochman JE, Powell N, Boxmeyer C, Andrade B, Stromeyer SL, Jimenez-Camargo LA. Adaptations to the coping power program's structure, delivery settings, and clinician training. *Psychotherapy*. 2012;49(2):135-142. doi:10.1037/a0027165

54. Stoltz S, Van Londen M, Deković M, De Castro BO, Prinzie P, Lochman JE. Effectiveness of an individual school-based intervention for children with aggressive behaviour: A randomized controlled trial. *Behav Cogn Psychother*. 2013;41(5):525-548. doi:10.1017/S1352465812000525

55. McHugh RK, Murray HW, Barlow DH. Balancing fidelity and adaptation in the dissemination of empirically-supported treatments: The promise of transdiagnostic interventions. *Behav Res Ther*. 2009;47(11):946-953. doi:10.1016/j.brat.2009.07.005

56. Marchette LK, Weisz JR. Practitioner Review: Empirical evolution of youth psychotherapy toward transdiagnostic approaches. *J Child Psychol Psychiatry Allied Discip*. 2017;58(9):970-984. doi:10.1111/jcpp.12747

57. Bilek EL, Ehrenreich-May J. An Open Trial Investigation of a Transdiagnostic Group Treatment for Children With Anxiety and Depressive Symptoms. *Behav Ther*. 2012;43(4):887-897. doi:10.1016/j.beth.2012.04.007

58. Kennedy SM, Bilek EL, Ehrenreich-May J. A Randomized Controlled Pilot Trial of the Unified Protocol for Transdiagnostic Treatment of Emotional Disorders in Children. *Behav Modif*. 2019;43(3):330-360. doi:10.1177/0145445517753940

59. Chorpita BF, Weisz JR, Daleiden EL, et al. Long-term outcomes for the Child STEPs randomized effectiveness trial: A comparison of modular and standard treatment designs with usual care. *J Consult Clin Psychol*. 2013;81(6):999-1009. doi:10.1037/a0034200

60. Weisz JR, Chorpita BF, Palinkas LA, et al. Testing standard and modular designs for psychotherapy treating depression, anxiety, and conduct problems in youth: A randomized effectiveness trial. *Arch Gen Psychiatry*. 2012;69(3):274-282. doi:10.1001/archgenpsychiatry.2011.147

61. Weisz JR, Bearman SK, Ugueto AM, et al. Testing Robustness of Child STEPs Effects with Children and Adolescents: A Randomized Controlled Effectiveness Trial. *J Clin Child Adolesc Psychol*. 2019;00(00):1-14. doi:10.1080/15374416.2019.1655757

©2020 Jeppesen P et al. *JAMA Psychiatry*.

62. Rubin DB. *Multiple Imputation for Nonresponse in Surveys*. J. Wiley; 1987.

©2020 Jeppesen P et al. *JAMA Psychiatry*.
