# Supplementary material for: Effectiveness of Transdiagnostic Cognitive-Behavioral Psychotherapy Compared With Management as Usual for Youth With Common Mental Health Problems: A Randomized Clinical Trial
Source: JAMA Psychiatry. 2020 Dec 23;78(3):1–12. doi: 10.1001/jamapsychiatry.2020.4045 (PMC7758821; doi:10.1001/jamapsychiatry.2020.4045)
Supplement: Supplement 3. — Data Sharing Statement [file jamapsychiatry-e204045-s003.pdf]

Data Sharing Statement

Data

Data available: Yes
Data types: Deidentified participant data
How to access data: The pseudonymous data can be made available from six months after the publication date of this Article, and with no end date. Proposals for use of data and requests for access should be directed to pia.jeppesen@regionh.dk. To gain access, researchers will need to sign a data access agreement with the Research Unit of the Child and Adolescent Mental Health Centre - Capital Region of Denmark, Copenhagen, Denmark.
When available: With publication

Supporting Documents

Document types: Other (please specify)
Additional Information: Content of Supplement 2 List of Investigators Steering Committee Evidence for the Effectiveness of CBT for Anxiety in Youth Evidence for the effectiveness of CBT for Depressive Symptoms and Disorders in Youth Evidence for the Effectiveness of Parent Training and CBT for Behavioral Problems and Disorders in Youth Methods - Interventions in Mind My Mind (MMM) Rationale for Transdiagnostic and Modular CBT Content of the Transdiagnostic and Modular Treatment in MMM Individually Adapted Transdiagnostic Treatment in MMM Methods - Management As Usual (MAU) Figure S1: The Prototypical Program for Anxiety in MMM Figure S2: The Prototypical Program for Depressive Symptoms in MMM Figure S3: The Prototypical Program for Parent Training in MMM Figure S4. Scatterplot of the correlation between the number of sessions and the primary outcome measure at 18 weeks, in the MMM intervention group (observed values)* Table S1. Potential Harms and Negative Outcomes at 18 Weeks and at 26 Weeks (The ITT Population)* Table S2. Other Exploratory Outcomes: Change from Baseline at 18 Weeks and 26 Weeks (The ITT Population)* Table S3. Change from Baseline in Primary and Secondary Outcomes at 18 Weeks in the ITT Population: Missing Data Handled Using Multiple Imputation* Table S4. Exploratory Outcomes: Change from Baseline at 26 Weeks in The ITT Population: Missing Data Handled Using Multiple Imputation* Table S5. Change from Baseline in Primary and Secondary Outcomes at 18 Weeks: Per Protocol Population* Table S6. Change from Baseline in Primary and Secondary Outcomes at 18 Weeks: Before Trial Registration* Table S7. Change from Baseline in Primary and Secondary Outcomes at 18 Weeks: After Trial Registration* Table S8. Correlation matrix exploring the inter-correlations of the primary and key secondary outcomes

(change scores) in the MMM group* Table S9. Correlation matrix exploring the inter-correlations of the primary and key secondary outcomes (change scores) in the MAU group* Table S10. Subgroup analysis for the observed parent-reported SDQ-Impact scores (n=343) References

How to access documents: Supplement 2 is uploaded for publication (additional online materials)

When available: With publication

Additional Information

Who can access the data: The pseudonymous individual participant data that underlie the results reported in this article, (text, tables, figures, and appendices) can be made available to investigators for individual participant data meta-analyses that have been approved by independent review committees. The data access will be granted on a case-by-case basis by the principal investigator (Pia Jeppesen) after further approval by the nongovernmental organization Psykiatrifonden (who was responsible for the implementation of the MMM program in the municipalities and has the legal responsibility as the data controller for the Web-based data collection). Access will be granted to the extent permissible by the General Data Protection Regulation and the Danish Data Protection Act. Making the data available may require approval from the Danish Data Protection Authority.

Types of analyses: Meta-analyses

Mechanisms of data availability: With a signed data access agreement, followed by investigator support

Any additional restrictions: Access will be granted to the extent permissible by the General Data Protection Regulation and the Danish Data Protection Act. Making the data available may require approval from the Danish Data Protection Authority.
